# Dynamic Alteration of the Gut Microbiota Associated with Obesity and Intestinal Inflammation in Ovariectomy C57BL/6 Mice

**DOI:** 10.1155/2022/6600158

**Published:** 2022-01-22

**Authors:** Hui Zhao, Qi Wang, Liqiu Hu, Shaojun Xing, Hui Gong, Zhe Liu, Panpan Qin, Jie Xu, Jihui Du, Wen Ai, Songlin Peng, Yifan Li

**Affiliations:** ^1^Department of Clinical Laboratory, Huazhong University of Science and Technology Union Shenzhen Hospital, Shenzhen 518052, China; ^2^Cuiying Biomedical Research Center, Lanzhou University Second Hospital, Lanzhou 730010, Gansu, China; ^3^Department of Spine Surgery, Shenzhen People's Hospital, Jinan University Second College of Medicine, Shenzhen 518020, China; ^4^Guangdong Provincial Key Laboratory of Regional Immunity and Diseases, Department of Pathogen Biology, Shenzhen University Health Science Center, Shenzhen 518060, China; ^5^Department of Computer Sciences, City University of Hong Kong, Hong Kong 999077, China; ^6^Qingdao-Europe Advanced Institute for Life Sciences, BGI-Shenzhen, Qingdao 266555, China; ^7^Shenzhen Key Laboratory of Human Commensal Microorganisms and Health Research, BGI-Shenzhen, Shenzhen 518083, Guangdong, China; ^8^Medical Research Center of Huazhong University of Science and Technology Union Shenzhen Hospital, Shenzhen 518102, China

## Abstract

**Objective:**

Estrogen is a critical hormone that is mainly produced by the ovary in females. Estrogen deficiency leads to various syndromes and diseases, partly due to gut microbiota alterations. Previous studies have shown that estrogen deficiency affects the gut microbiota at 6–8 weeks after ovariectomy, but the immediate effect of estrogen deficiency on the gut microbiota remains poorly understood.

**Methods:**

To investigate the short time and dynamic effects of decreased estrogen levels on the gut microbiota and their potential impact on estrogen deficiency-related diseases, we performed metagenomic sequencing of 260 fecal samples from 50 ovariectomy (OVX) and 15 control C57BL/6 female mice at four time points after surgery.

**Results:**

We found that seven gut microbiota species, including *E. coli, Parabacteroides unclassified, Lachnospiraceae bacterium* 8_1_57FAA*, Bacteroides uniformis, Veillonella unclassified, Bacteroides xylanisolvens,* and *Firmicutes bacterium* M10_2, were abundant in OVX mice. The abundance of these species increased with time after OVX surgery. The relative abundance of the opportunistic pathogen *E. coli* and the Crohn's disease-related *Veillonella spp.* was significantly correlated with mouse weight gain in the OVX group. Butyrate production and the Entner–Doudoroff pathway were significantly enriched in the control mouse group, while the degradation of glutamic acid and aspartic acid was enriched in the OVX mouse group. As the time after OVX surgery increased, the bacterial species and metabolic pathways significantly changed and tended to suggest an inflammatory environment, indicating a subhealthy state of the gut microbiota in the OVX mouse group.

**Conclusions:**

Taken together, our results show that the dynamic gut microbiota profile alteration caused by estrogen deficiency is related to obesity and inflammation, which may lead to immune and metabolic disorders. This study provides new clues for the treatment of estrogen deficiency-related diseases.

## 1. Introduction

Estrogen is the collective name for chemically similar hormones, including estrone, estradiol, estriol, and estetrol, that are mainly secreted by the ovary. The estrogen cycle in females is an essential component of the endocrine system regulating many characteristics of the body in females, such as obesity [1], the production of multiple immune cytokines [2], vascular wall cells [3, 4], and bone metabolism [5, 6].

Estrogens play a significant regulatory role in the innate and adaptive immune systems [7, 8]. Estrogens regulate gene expression by binding to their receptors. It has been reported that estrogen receptors are expressed in thymocytes and thymic epithelial cells, which are crucial lymphatic organs and places where T cells differentiate, develop, and mature [9]. Estrogens can modulate immune activities in the body and be involved in autoimmune diseases, such as rheumatoid arthritis (RA) and systemic lupus erythematosus (SLE) [8]. In summary, estrogens can directly regulate the immune system through their effects on various immune cells and cytokines during immune system development, in autoimmune diseases, and in infectious diseases.

Estrogens are also critical for modulating the metabolic system, such as obesity [10, 11]. Obesity is one of the severe problems related to estrogen deficiency in females and has become a worldwide epidemic, especially in developed countries [12]. Although obesity does not cause physical discomfort, it has become a risk factor for breast cancer, ovarian cancer, endometrial cancer, and colon cancer. Cancer patients with obesity have a higher mortality rate and worse prognosis than patients with a normal weight [13]. Second, it is increasingly recognized that obesity is also a risk factor for cardiovascular disease and hypertension [14, 15] and is correlated with the development of microvasculature [15]. Obesity can influence the microvasculature of multiple tissues, including visceral adipose tissue, skeletal muscle, and the heart, leading to changes in the release and clearance of neurohumoral factors, such as adipokines and inflammatory cytokines [14]. Therefore, obesity is a risk factor for certain diseases that needs to be better understood and properly controlled.

Along with the direct regulatory effect of estrogens on host cells, emerging evidence suggests that estrogens may also play an indirect role in diseases by regulating the gut microbiota. Previous studies have revealed that small amounts of brain estrogens can improve menopausal symptoms by decreasing serum FSH levels and maintaining the diversity of the gut microbiota in estrogen-deficient rats [16], suggesting a potential role and mechanism of how reduced estrogen levels lead to less richness of the gut microbiota. It is now becoming clear that the gut microbiota is associated with some metabolic and immune system diseases, such as obesity [17], bone mineral loss [18], Crohn's disease, and inflammatory bowel disease (IBD) [19]. In addition, the gut microbiota has been proposed to be a therapeutic target for antiobesity to regulate metabolism and the energy balance [17].

Estrogen deficiency is a consistent lack or decreased levels of estrogens and adversely affects the female body. Estrogen deficiency disrupts the menstrual cycle and leads to many unpleasant symptoms, such as menopausal symptoms, cardiovascular diseases, and osteoporosis. Since the gut microbiota is associated with metabolism, immunity, and endocrine system diseases [20, 21] and is assumed to contribute to estrogen deficiency-related diseases, alteration of the gut microbiota caused by the reduction in estrogens may be an important factor that increases the risk of the complex diseases mentioned above. Previous studies on estrogen deficiency and the gut microbiota have included the removal of both ovaries to study OVX mice and the collection of mouse fecal samples 6–8 weeks after ovariectomy [22, 23], which is a long period of time. Although factors such as diet and the environment may be controlled in experiments, measuring the gut microbiota one month after ovariectomy may affect by some cofounders, such as the exercise time of the mouse. It is important to understand the effect of estrogen deficiency on the gut microbiota in a short time and its related metabolic and immune diseases. To investigate the dynamic alterations of the gut microbiota in a short period of time after OVX surgery, an estrogen-deficient mouse model was established by performing ovariectomy surgery on C57BL/6 female mice. We collected fecal samples at four time points, once before surgery and once a week after surgery, three times. We found a dynamic process of gut microbiota profile alteration within one month after OVX surgery. We identified gut microbiota species and pathways that are related to the inflammatory gut environment and body weight regulation.

## 2. Materials and Methods

### 2.1. Mice

Sixty-five female C57BL/6 mice at the age of 8 weeks were purchased from the Medical Laboratory Animal Center of Guangdong Province (Guangdong, China). The experiment was conducted following the National Guidelines for Animal Use in Research (China). C57BL/6 female mice generally reach the maturity stage in approximately eight weeks.

All mice in this study were kept in animal rooms after purchase, and all of the 65 mice acclimatized to their new environment for three days. We used SPF mouse-breeding cages, with an average of 5 SPF mice placed in each cage. To optimize environmental conditions to reduce mouse stress and maintain the same rhythm of all the 65 mice in this study, the lights in the animal room were turned on at 8 ′am and turned off at 8 ′pm every day. After acclimatization, C57BL/6 female mice were randomly divided into three groups that received different treatments. We started the experiment (marked as Day 0), and on the Day 0 of our experiment, we first collect mice fecal samples between 2 pm and 4 pm as the light was still on and then carry out OVX surgery on 50 mice and SHAM operation on 9 mice, and the other 6 mice were kept intact. When collecting feces, the experimenter grabs the mice to defecate under stress or puts them in a separate cage and collects two feces each time. Once a week, on Day 7, Day 14, and Day 21 of the experiment between 2 pm and 4 pm, we collect mice fecal samples in the same light condition, and a total of 260 mouse fecal samples were collected. All samples were immediately frozen in dry ice and stored at -80 °C until they were used for sequence and analysis. We measured the mouse weight at Day 0, Day 7, Day 14, and Day 21, and these four time points were marked as T1, T2, T3, and T4, respectively (Supplementary Figure 1). The initial mouse weight at the T1 time point was 20 ± 2 g. Mouse weight is recorded in Supplementary Table 1. Mice were maintained in a specific pathogen-free (SPF) environment and fed a normal rodent diet. The mouse feed we purchased was the SPF-grade “growing and reproductive feed for rats and mice” from BEIJING KEAO XIELI FEED Co., Ltd. (feed introduction URL: http://www.keaoxieli.com/product/136.html). This mouse feed contains 3.44 kcal energy per gram, and the protein-energy ratio is 24.02%, the fat energy ratio (percent calories from fat) is 12.95%, and the carbohydrate energy ratio is 63.03%.

In terms of sample size selection, we found that the number of mice in most studies is more than 10 per group, and mice number of around 10 for each group is appropriate and acceptable [24–26]. In our study, the mice number in OVX and control groups is above 10. Furthermore, we chose more mice to undergo ovariectomy (*n* = 50), which is more than 15 mice in the control group. To understand whether the sample size difference affects the statistics, we used SMOTE algorithm to construct new control group samples based on the relative abundance of the gut microbiota species and generated a new control group with 50 samples. This makes the case number of each group equal. Then, we conducted Wilcoxon rank-sum test to compare the gut microbiota species difference between the OVX group (*n* = 50) and the new control group (*n* = 50) and between the OVX group (*n* = 50) and the original control group (*n* = 15) (Supplementary Table 2). From this result, we found that either using the SMOTE algorithm that constructs the new control group (*n* = 50) or using the original control group (*n* = 15) to compare with the OVX group, we see the same significant differences in most gut microbiota species. Besides, we conducted the kernel density estimation (KDE) method to compare the probability density difference between the new control group (*n* = 50), which is constructed by the SMOTE algorithm, and the OVX samples (*n* = 50) (Supplementary Figure 5). We also compared the original control group (*n* = 15) with the OVX group (*n* = 50) using the KDE method (Supplementary Figure 6). From this result, we found that the difference in the relative abundance of the gut microbiota species for 50 OVX mice compared with 50 control mice group and 50 OVX mice compared with 15 control mice was highly similar. Therefore, the difference in gut microbiota species in this study does not cause by the imbalance of the sample size.

We performed ovariectomy (OVX) surgery to generate an estrogen-deficient mouse model with fifty mice in the first group and SHAM operation with nine mice in the second group. The third group was the negative group with six mice, which were kept intact without surgery. The SHAM group (9 mice) and the no operation group (6 mice) were merged to form the control group (15 mice), which was used as the control in this work.

### 2.2. DNA Preparation and Mice Metagenomic Sequencing

Mouse fecal samples were collected from the Clinical Medicine Transformation Center of Shenzhen People's Hospital and immediately frozen in a -80 °C freezer. The samples were transported to BGI-Shenzhen on dry ice. The bacterial DNA extraction experiments were performed using MagPure Fast Stool DNA KF Kit B following the protocols of the BGI-Shenzhen [27], in accordance with the manufacturer's instructions or protocols. This method performs high-accuracy extraction of bacterial DNA and has higher extraction efficiency for Gram-positive bacteria that are difficult to lyse. The extracted DNA quality was measured by agarose gel electrophoresis and a Qubit® 3.0 Fluorometer (Thermo Fisher, Waltham, MA, USA), following procedures described in reference [28]. Single-end metagenomic sequencing was conducted on the BGISEQ-500 platform (BGISEQ, RRID : SCR_017979, read length, 100 bp) [29]. Quality control was performed, and adaptor and host contamination was filtered. The sequencing data of the 260 fecal samples of mice collected at the four time points included a total of 232.37 million reads per sample; the average Q30 of each sample was 93.64% (Supplementary Table 3). Detailed information on the data size and removal host rates is presented in Supplementary Table 3.

### 2.3. Taxonomic Annotation and Abundance Calculation

The reads were aligned to the mouse gene catalog, which comprises 2.6 million nonredundant genes, after removing low-quality reads and mouse host genome reads with an in-house pipeline [30]. Then, we obtained the gene profile, which consisted of the relative abundance of each sample for each gene. Aiming to evaluate bacterial quantitative performance in human fecal samples, we performed taxonomic annotation and quantification using MetaPhlAn2 (metagenomic phylogenetic analysis, version 2.7.0) and generated microbial profiles including bacteria, eukaryotes, archaea, and viruses for all 260 mice fecal samples [31]. The method calculates taxonomic relative abundance that inferred to the presence and read coverage in a microbiome sample.

### 2.4. *α*-Diversity and Counts

*α*-Diversity (within-sample diversity) was calculated based on the taxon profile of each sample according to the Shannon index [32]. The total taxon count in each fecal sample was determined as described in reference [33].

### 2.5. Statistical Analysis

The Shannon index was calculated with the vegan package (V 2.5–6) in *R* software (V 3.4.0). To compare the gut microbiota species distribution in the three groups of mice, we carried out principal coordinate analysis by using the ape package (V 5.4–1) in R. The first two principal components at the genus and species levels are presented in Supplementary Table 4. We used the Benjamini-Hochberg (BH) method to correct the *p* values determined by the Wilcoxon rank-sum test. To investigate the gut microbiota species contributing to the significant weight gain after OVX surgery, we conducted Spearman's correlation analysis on the mice weight and the relative abundance of gut microbiota species with cor.test function in *R* software (V 3.4.0). The enrichment of the gut microbiota species was determined by the Wilcoxon rank-sum test (*p* < 0.05) and corrected by the BH method in *R* by wilcox.test and p.adjust functions.

### 2.6. Handling of Unbalanced Datasets with the Synthetic Minority Oversampling Technique (SMOTE) Method

SMOTE is an oversampling algorithm, and it can construct new small-class samples instead of generating copies of existing small-class samples [34, 35]. The data constructed by this algorithm are a new sample that does not exist in the original dataset. We adopted the method of constructing artificial data samples of the SMOTE (synthetic minority oversampling technique) system to solve the problem of the imbalance of positive and negative sample data. The algorithm selects two or more similar samples in a small category based on the distance metric, then selects one of the samples, randomly selects a certain number of neighbor samples to add noise to an attribute of the selected sample, and processes one attribute at a time. In this way, more new data are constructed. In this study, we import the python module “imblearn” to implement the SMOTE algorithm.

### 2.7. Functional Annotation and Identification of GMMs

Gut metabolic module (GMM) determination was carried out as a functional classification method based on a set of manually curated reference modules developed by Sara Vieira-Silva [36]. The abundance of each GMM was calculated by using the median KO abundance with 66% coverage. The enrichment was determined by the Wilcoxon rank-sum test (*p* < 0.05) and corrected for multiple testing using the BH method.

### 2.8. Availability of Data

The sequencing data for the 260 mouse fecal samples were deposited in the China National GeneBank Database (CNGB) with the accession number CNP0001300.

## 3. Results

### 3.1. The Microbial Distribution in the SHAM Group Is Consistent with That in the Control Group

**Figure 1:**
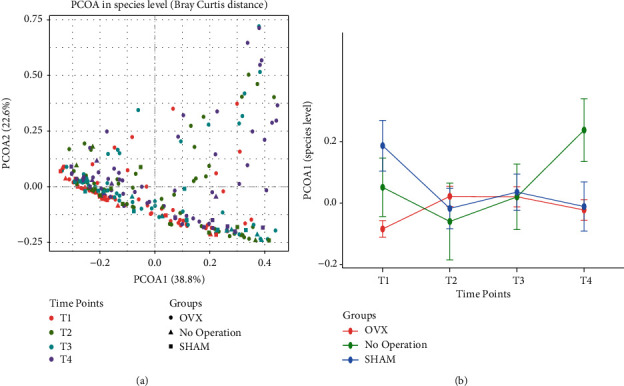
The distribution of the gut microbiota species in the SHAM group is highly consistent with that in the no operation group. The Bray–Curtis distance based on the species relative abundance was calculated. (a) The independent distribution of the gut microbiota species in the OVX, no operation, and SHAM group. The percentages of the first and second principal components are 38.8% and 22.6%, respectively. (b) Line plot of the three groups of mice at different time points.

This study collected 260 fecal samples from three groups of mice at four time points (T1-T4). Based on Bray–Curtis distance in the genus and species levels, we conducted principal coordinate analysis (PCOA) to identify gut microbiota differences among the three mouse groups at four time points. The distribution of gut microbiota at the species level (Figures 1(a) and 1(b)) and genus level (Supplementary Figure 2) according to the first two principal components with the highest proportions in SHAM group mice was similar to that in no operation group, suggesting that the composition of the gut microbiota was similar in the SHAM and no operation groups at four time points. Therefore, in the subsequent analysis, we defined a new control group that includes the SHAM group (9 mice) and the no operation group (6 mice), resulting in 15 mice in the control group. After regrouping the mice, there were a total of 15 mice in the control group and 50 mice in the ovariectomized (OVX) group.

### 3.2. The Gut Microbiota Profile in the OVX group Is Significantly Altered Compared to That in the Control Group

The difference in gut microbiota richness at the gene (Supplementary Figure 3) and species (Supplementary Figure 4) levels between the OVX and control group mice was not significant at any of the T1, T2, T3, and T4 time points.

**Figure 2:**
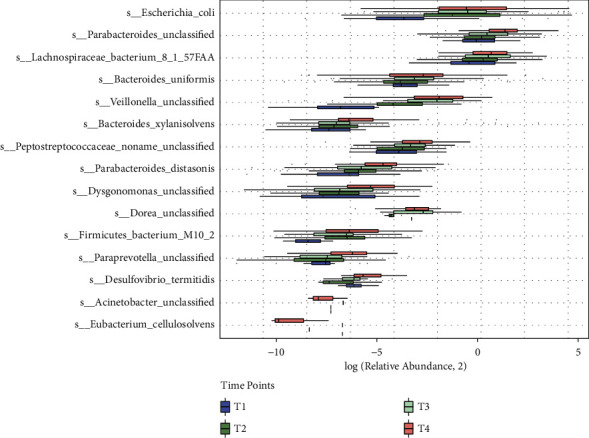
Alteration of the gut microbiota species abundance with significant differences in the OVX group at different time points after OVX surgery. This figure shows the results of the data analysis of the gut microbiota at the species level in the OVX group. The species that significantly differed in terms of abundance were determined by the Kruskal–Wallis test (*p* < 0.05). The abscissa is the relative abundance plus a minimum value (1E-10), and then, the log2 value was calculated. The ordinate is four time points. Fifteen gut microbiota species had significantly different abundance between the two groups. The icons on the right represent different time nodes. The abscissa is the relative abundance plus a minimum value (1E-10), and then, the log2 value was calculated. The ordinate is four time points. Fifteen gut microbiota species had significantly different abundance between the two groups. The icons on the right represent different time nods.

To investigate the effect of estrogen deficiency on the gut microbiota during the T1-T4 period, we performed Kruskal–Wallis (KW) tests at four time points in the OVX group. The differences in the gut microbiota species in the OVX group at the T1-T4 time points are presented in Supplementary Table 5. We also performed a comprehensive ranking of the Wilcoxon rank-sum test results of the four time points and selected 15 gut microbiota species with significant differences at the T1-T4 time points. We found that the differences in the gut microbiota species between almost every two time points were statistically significant (Kruskal–Wallis test: *p* < 0.05; Supplementary Table 5). Our results indicated that the relative abundance of the gut microbiota species, including *E. coli, Parabacteroides unclassified, Lachnospiraceae bacterium* 8_1_57FAA*, Bacteroides uniformis, Veillonella unclassified, Bacteroides xylanisolvens, Peptostreptococcaceae noname unclassified, Parabacteroides distasonis, Dysgonomonas unclassified, Firmicutes bacterium* M10_2, and *Paraprevotella unclassified*, increased over time after OVX surgery (Figure 2), suggesting that the abundance of these gut microbiota gradually increased with the reduction in the estrogen level in mice. The relative abundance of the gut microbiota species, including *Dorea unclassified, Acinetobacter unclassified,* and *Eubacterium cellulosolvens*, were not significantly altered at the T1-T4 time points. Together, our results show significant changes in the relative abundance of some gut microbiota species after estrogen levels were reduced at multiple time points.

**Figure 3:**
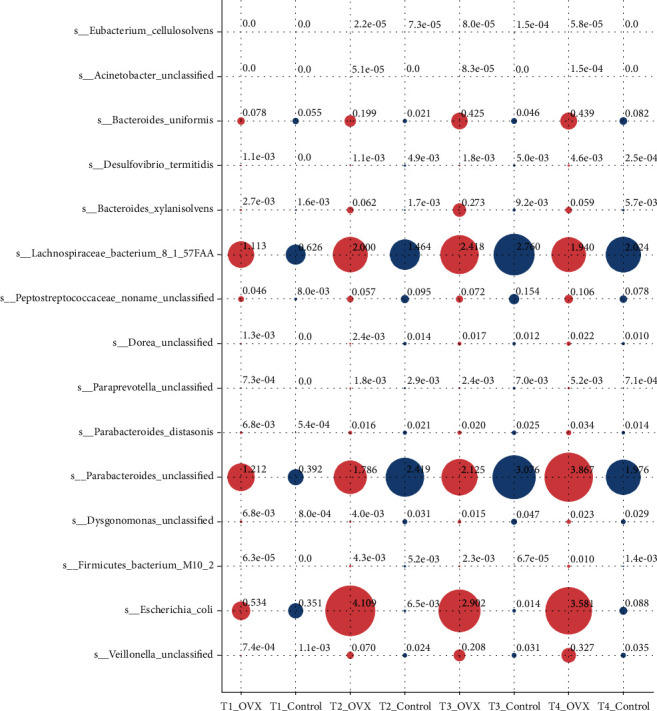
Significant differences in the gut microbiota species abundance between the OVX and control groups at different time points. This figure shows that the 15 gut microbiota species had significantly different abundance between the OVX and control groups at four time points. The abscissa shows different time points, and the ordinate shows 15 significantly different gut microbiota. This size of each point represents the relative abundance of the corresponding gut microbiota at a specific time point and by group. The numbers at the upper right corner of each point mark the relative abundance of the gut microbiota species represented by each point. The OVX group and the control group are colored red and blue, respectively.

To explore the differences in the above 15 gut microbiota species between the OVX group and the control group, we carried out Wilcoxon rank-sum tests to compare them at the T1-T4 time points (Figure 3). At the T1 time point, the relative abundance of the gut microbiota species, including *Parabacteroides unclassified, Parabacteroides distasonis,* and *Peptostreptococcaceae noname unclassified*, between the OVX group and the control group were significantly different (Wilcoxon rank-sum test *p* < 0.05; BH correction: *q* > 0.05; Supplementary Table 6). At the T2 time point, the significantly different gut microbiota species between the OVX and control groups included *Veillonella unclassified, Escherichia coli, Dysgonomonas unclassified, Peptostreptococcaceae noname unclassified, Desulfovibriotermitidis, Bacteroides uniformis, Acinetobacter unclassified, and Eubacterium cellulosolvens* (Figure 3; Supplementary Table 6). At the T3 time point, gut microbiota species including *Bacteroides uniformis, E. coli,* and *Veillonella unclassified* were abundant in the OVX group, and the relative abundance between the OVX and control groups were significantly different. In contrast, *Dysgonomonas unclassified* was depleted in the OVX group. At the T4 time point, a cluster containing *Veillonella unclassified, Escherichia coli,* and *Firmicutes bacterium* M10_2was enriched in the OVX group. The gut microbiota species between the OVX and control groups were also significantly different.

### 3.3. Estrogens Contribute to Mouse Weight Regulation, Which Is Associated with Gut Microbiota Taxonomic Signatures

**Figure 4:**
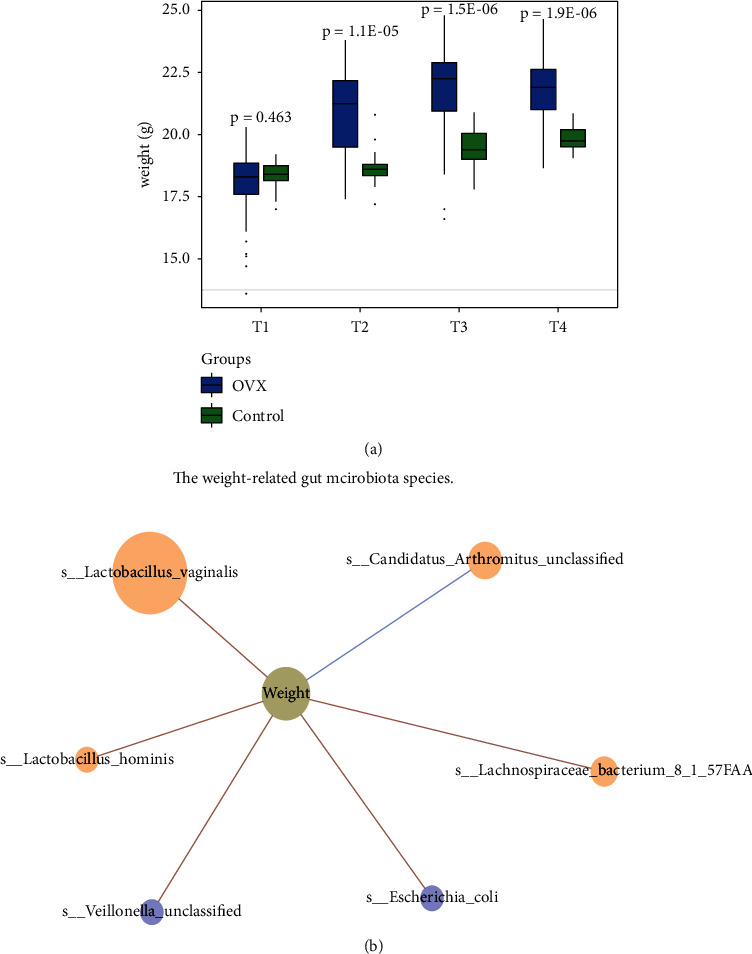
Estrogen deficiency leads to an increase in mouse weight and alters the abundance of gut microbiota species related to weight. (a) The boxplot shows the mouse weights of the model and control groups. The weight of the OVX group mice significantly increased compared to that of the control group mice. The *p* value was calculated by the Wilcoxon rank-sum test and corrected by the BH method. (b) The gut microbiota species were related to mouse weight. The red line represents the gut microbiota species that were positively correlated with mouse weight (Spearman's correlation coefficient >0.3 and Spearman's *p* value < 0.05). In contrast, the blue line represents the species that were negatively associated with mouse weight (Spearman's correlation coefficient < -0.3 and Spearman *p* value < 0.05). The blue nodes represent the species that were enriched in the OVX group mice at the T2-T4 time points, while the nodes in light orange represent the irregular enrichment of species at different time points. The node size indicates Spearman's *p* value of the gut microbiota species and mouse weight.

To evaluate the impact of estrogens on mouse weight, we measured mouse weight at the T1-T4 time points (Figure 4(a)). At the T1 time point, there was no significant difference in weight between the OVX group and the control group. However, as the time after OVX surgery increased, the body weight of the mice in the OVX group significantly increased compared with that of the mice in the control group.

Emerging evidence suggests that obesity is closely associated with the enrichment of gut microbiota species [37, 38]. To investigate whether the significant weight gain after OVX surgery is related to alterations of the gut microbiota alteration after estrogen levels are reduced, we conducted Spearman's correlation analysis on the weight-related gut microbiota. We displayed the result as a network (Figure 4(b))). We found that some species, including *Veillonella unclassified* and *E. coli*, showed a significantly positive correlation with mouse weight (Supplementary Table 7), and the abundance of these two species obviously increased as the time after OVX surgery was prolonged. Taken together, these results indicate that the reduction in the estrogen level in mice increases the abundance of *Veillonella* spp. and *E. coli*, which are associated with obesity in mice.

### 3.4. The Alteration of GMMs in the OVX Mice

Similar to the functional classification method of human gut samples, the GMM method is also applicable to other mammals, such as mice. To investigate the impact of estrogen deficiency on gut microbiota function in the OVX and control group mice, we analyzed gut microbiota function using the gut metabolic module (GMM) method. We found no significant difference in gut microbiota function between the OVX and control group mice at the T1 time point (Wilcoxon rank-sum test: *p* < 0.05; BH correction: *q* > 0.05; Supplementary Table 8). However, at the T2 time point, which was after OVX surgery, there were four significantly different GMMs between the two groups: the aspartic acid degradation II, arabinoxylan degradation, Entner–Doudoroff pathway, and butyrate production pathways (Supplementary Table 8). Among them, the Entner–Doudoroff and butyrate production pathways were significantly enriched in the control group mice, while the aspartic acid degradation II and arabinoxylan degradation pathways were enriched in the OVX group mice. At the T3 time point, the difference in gut microbiota function became obvious. There were 22 significantly different metabolic functions between the mice in the OVX and control groups at the T3 time point. In total, 11 biological functions, including arginine degradation III, butyrate production, the pyruvate dehydrogenase complex, galactose degradation, bifidobacterium shunt, triacylglycerol degradation, ectopic sugar degradation, and glycerol degradation I, were enriched in the control group mice. Eleven biological functions, including pectin degradation I, arabinoxylan degradation, fructan degradation, mucin degradation, starch degradation, sulfate reduction (alienation), glutamine amide degradation II, 4-aminobutyrate degradation, aspartic acid degradation II, and lactose degradation, were enriched in the OVX group mice. The butyrate production pathway was significantly enriched in the control group mice. Taken together, our results showed that as the time after OVX surgery increased, the gut microbiota metabolic pathways of the OVX group mice and the control group mice at the T2-T4 time points were significantly different from those at the T1 time point, which did not progress OVX surgery.

## 4. Discussion

Here, we characterized the alterations of the gut microbiota profile and metabolic pathways after the estrogen level was reduced and confirmed that estrogens contribute to C57BL/6 female mouse weight regulation. We identified that the abundance of the gut microbiota species, including *E. coli*, *Parabacteroides unclassified*, *Lachnospiraceae bacterium 8_1_57FAA*, *Bacteroides uniformis, Veillonella unclassified, Bacteroides xylanisolvens, Peptostreptococcaceae noname unclassified, Parabacteroides distasonis, Dysgonomonas unclassified, Firmicutes bacterium M10_2,* and *Paraprevotella unclassified,* increased over time after OVX surgery. *Bacteroides uniformis, E. coli,* and *Veillonella unclassified* were abundant in the OVX group. Unclassified *Dysgonomonas* were enriched in the control group. *Veillonella* spp. and *E. coli* were positively correlated with mouse weight. For metabolic pathways, we identified that the gut microbiota species involved in butyrate production in the OVX group were depleted, suggesting that estrogens may reduce intestinal inflammation by regulating the abundance of the butyric acid-producing gut microbiota species and may play a critical role in maintaining intestinal health.

In this study, we have generated the estrogen-deficient mouse model by removing bilateral ovaries, following the methods previously described [39–42]. This method has previously been used to study the effect of estrogen level reduction on bone mineral density and the immune system [39–41]. OVX surgery has also been used to establish a postmenopausal obesity mouse model, followed by feeding high-fat food [42]. The mice underwent ovariectomy in our study, and the main hormones secreted by the ovaries include estrogens, progesterone, and small amounts of androgens. Endogenous estrogens include estradiol, estrone, and estriol, among which the most important and biologically active estrogen is estradiol, which is mainly secreted by follicular cells [26]. After the mice undergo OVX surgery, their endogenous estrogen content and biological activity of estrogens are significantly reduced, which may be a major contributor to gut microbiota alterations.

The sex hormone factors have an impact on the gut microbiota in mice. Researchers previously found that sex differences exist in the gut microbiota composition in 89 different inbred strains of mice, especially in C57BL/6J and C3H/HeJ strains [43]. In female mice, OVX could alter the gut microbiota diversity [44]. E2 also had an impact on the gut microbiota taxon. A previous study reported that 17 *β*-estradiol (E2) has an impact on the gut microbiota taxa and described the alterations of the gut microbiome species in female, 17 *β*-estradiol-treated male, and ovariectomized mice; this study showed that *Proteobacteria* decreased and were associated with lower susceptibility to metabolic syndrome [45].

The *α*-diversity of the mouse gut microbiota in our study did not significantly change. This finding is different from a previous study showing that the *α*-diversity of the gut microbiota was reduced, with significant gut dysbiosis in reproductively senescent females (naturally estrogen deficient) [46]. This difference might be related to the reduction in estrogen levels. We performed OVX surgery to remove the ovary instead of inducing a natural estrogen deficiency. Our study design was closer to the reduced estrogen levels caused by unnatural menopause, which may be relevant in some clinical situations, such as ovarian removal in ovarian tumors, ovarian corpus luteum cysts or flavin cysts, and ovarian endometriosis [47, 48]. Intriguingly, it has been proposed that gut microbiota modification could be a therapeutic target for modulating the pathophysiology of ovarian cancer [49].

We showed that the abundance of *Bacteroides* spp. and *Firmicutes bacterium* were increased in the OVX group mice at the age of 8 weeks to 12 weeks, leading to no significant change in the F/B ratio. This finding is different from a previous phylum-level analysis showing that compared to SHAM group mice, 16-week-old OVX group mice have a reduced population of *Bacteroidetes* and an increased population of *Firmicutes*, resulting in a higher ratio of *Firmicutes* to *Bacteroidetes* in the gut [23]. The different F/B ratios between the two studies may be related to the sampling time points after OVX surgery, suggesting a continuous change in the gut microbiota species 12–16 weeks after OVX surgery. In addition, we showed that the relative abundance of *Parabacteroides unclassified* increased in the OVX group mice. This finding is consistent with a previous report [50].

At the T1 time point, there was no significant difference in the relative abundance of *E. coli* between the two mouse groups. However, as the time of ovarian removal was prolonged, the relative abundance of *E. coli* in the OVX group mice significantly increased. In contrast, *E. coli* was gradually depleted in the control group mice. *E. coli* is an opportunistic pathogen that can cause infection and inflammatory bowel disease when host body resistance is decreased [51]. Therefore, estrogen reduction leading to an increase in *E. coli* abundance may increase the chance of opportunistic infection.

The difference in the species *Veillonella unclassified* between the OVX and control groups gradually became apparent over time. The reduction in estrogen levels leads to an increase in the relative abundance of *Veillonella* spp. Previous studies have shown that the relative abundance of *Veillonella spp.* is significantly increased in the upper gastrointestinal tract of patients with Crohn's disease [52–54]. The abundance of *Veillonella* spp. in our OVX group mice indicated that estrogen reduction leading to an increase in this species might be related to an inflammatory environment and a subhealthy condition of the gut. It is interesting to note that, in most autoimmune diseases, there is a gender bias and the impact of hormones factor, and research found that germ-free mice lost the gender bias for the incidence of T1D, indicating that sex hormones and microbes can work together to trigger the protective pathways of autoimmune diseases [55]. While autoimmune diseases could cause systemic self-inflammation [56], translocation of a gut pathobiont triggers autoimmune responses [57]. Therefore, *Veillonella* spp. and *E. coli* might act as a gut pathobiont that influenced the immunity system and caused the inflammation response.

In this study, we fed all 65 female mice with a normal rodent diet. Interestingly, research showed that diet could interact with sex hormones to influence the gut microbiota composition. For example, the effect of a high-fat diet on gut microbiota composition changes is more pronounced in female mice than in male mice [43]. 17 *β*-Estradiol (E2) was associated with reduced gut microbial diversity in high-fat diet-fed female mice [44]. Thus, there are sex differences in gut microbiota in regard to HFD.

We showed that mouse weight in the OVX group significantly increased. It was shown that E2 attenuated weight gain in high-fat diet-fed female mice [44]. Studies have shown that estrogens contribute to metabolic modulation through estrogen receptor-*α* (ER-*α*) [11]. The body weight of estrogen receptor knockout mice is significantly increased compared to that of wild-type mice [58]. Female mice lacking estrogen receptor-*α* (ER-*α*) show a significant increase in white adipose tissue, but brown adipose tissue is not affected. Therefore, estrogens affect the weight of ER-*α* by regulating white adipose tissue in female mice [59]. Besides, estrogens also play a critical role in fat tissue metabolism [60] by controlling triglyceride and VLDL-TG content in plasma [61]. Regarding the other metabolic pathways, body weight regulation by E2 might be related to energy metabolism; for instance, OVX increases energy intake and obesity [44]. Obesity is a risk factor for hypertension, hyperlipidemia, and diabetes [62]. Therefore, the regulatory effect of estrogens on body weight is vital to health.

We found that OVX leads to two results: the first one is the changes in the gut microbiota, and the second one is the increase in the mouse weight. At the T2 time point, the earliest time point when we can observe the changes, we found both relative abundance of gut microbiota taxon alteration and a significant increase in the weight of the OVX group compared to the control group. Therefore, we can be sure that estrogens directly or indirectly lead to changes in the gut microbiota and an increase in weight. Although we found that *Veillonella* spp. and *E. coli* are positively associated with the increase in mouse weight (Figure 4), we do not have evidence to show whether obesity is involved in the process of the gut microbiota alteration by estrogens. The mice feed contains 12.95% calories from fat, which is not a high-fat diet. It is unlikely that the gut microbiota alteration is due to obesity caused by a high-fat diet. We are also not sure whether the gut microbiota alteration is involved in regulating obesity by estrogens. In future experiments, it is necessary to conduct a fecal transplantation experiment of *Veillonella* spp. and *E. coli* to control mice to explore whether estrogens contribute to obesity via changes in the gut microbiota. To explore whether estrogens lead to changes in the gut microbiota through obesity, it is possible to perform an experiment by intervening obesity with a high-fat diet of E2-rescued OVX mice, whose body weight is predicted to be rescued by E2.

We found that butyrate production was enriched in the control group mice but was not abundant in the OVX group mice. Butyrate, which is a kind of SCFA, can induce Treg-cell differentiation and regulate intestinal immunity, maintaining intestinal mucosa integrity and alleviating the development of inflammatory bowel diseases, such as colitis [63]. The butyrate-producing gut microbiota provides energy for intestinal mucosal cells, increases the absorption area, regulates the intestinal mucosal epigenetic and mineral absorption signal pathways, and promotes intestinal calcium absorption [64]. Therefore, the anti-inflammatory properties of the gut microbiota in the OVX group mice were inhibited by reduced butyrate production, which is likely to increase the chance of infection and related symptoms, suggesting a subhealthy state of the mouse gut in the OVX group.

Previous studies on mouse gut microbiota function are usually based on the GO or KEGG databases, which do not provide information on the microbiome involved in metabolic pathways. We carried out GMMs to classify the gut microbiota metabolic pathways and describe the functional changes within a short period of time after OVX surgery. The method has been previously used to study the species-function relationships in gut microbial genomes and microbiomes [36]. The GMM classification method that we used could supplement the metabolic function analysis of the mouse gut microbiota.

To the best of our knowledge, the time at which the mouse gut microbiota stabilizes after OVX surgery has not been previously reported, and alteration of the gut microbiota immediately after ovarian removal has not yet been revealed. We collected fecal samples once before OVX surgery and once a week three times within one month after OVX surgery. We showed the dynamic alteration of the gut microbiota in a short period of time after estrogen depletion. As the samples we collected were closer to the ovarian removal time than those examined in other studies, our results should better represent the impact of reduced estrogen levels on the gut microbiota, less affected by other irrelevant factors, such as the environment, the diet, or the exercise times of the mice.

In contrast, in previous relevant studies, fecal samples were collected a long time after various treatments. For example, in a study about the impact of resistant starch on bone loss in OVX mice, they used fecal samples collected at approximately 40 days after different treatments [22]. Similarly, in research showing that the proanthocyanidin-rich grape seed extract regulates the gut microbiota in OVX mice, the authors collected fecal samples eight weeks after the OVX and SHAM operations [23]. In addition, some studies on estrogen deficiency and the gut microbiota have established an OVX mouse model by removing both ovaries and collecting mouse fecal samples 6–8 weeks after experiments. The mouse gut microbiota can also be affected by factors such as diet and the environment [38]. For 6–8 weeks, external environmental factors may have affected the gut microbiota profile rather than estrogen reduction alone. Thus, our results showing the alteration of the gut microbiota within a short period of time after OVX surgery are more likely to represent the effect of the estrogen level reduction rather than other irrelevant environmental factors.

This work has limitations. First, although butyrate has been shown to exhibit anti-inflammatory properties and play a critical role in maintaining intestinal health by combining with the butyrate receptor GPR109 A in the colon [65], ovariectomy can drive inflammation based on microbial profiles, which needs to be further confirmed with inflammation markers and histology data in the future. Second, to support the idea that the main cause of the phenotype observed in ovariectomized mice is lack of estrogens, a rescue experiment by giving the operated mice with ectopic estrogens (for example, with a subcutaneous pellet) is necessary in the future.

Taken together, the results of our study revealed a dynamic change in the gut microbiota and its metabolic pathways in mice within one month after OVX surgery. We showed that the gut microbiota might produce less butyrate and shape an inflammatory gut environment in the OVX group mice than in the control group mice. We also showed that mouse weight was significantly and positively correlated with the estrogen level reduction and was strongly associated with the increased abundance of some gut microbiota species, suggesting that estrogens might contribute to weight regulation by affecting the gut microbiota. Our results provide new insights into potential targeted therapies via microbiota to relieve the intestinal inflammatory environment and relevant diseases caused by reduced estrogens.

## Data Availability

The sequencing data for the 260 mouse fecal samples were publicly available from https://ftp.cngb.org/pub/CNSA/data3/CNP0001300/.

## Supplementary Materials

Supplementary Materials Supplementary Figure 1: the timeline of different treatment and sample collection in this study. At the T1 time point, three days after the eight-week-old mice were acclimatized, we first collected the fecal samples. Then, the mice underwent SHAM operation and OVX surgery. We collected mice fecal samples once a week since the T1 time point within one month. Supplementary Figure 2: the gut microbiota at the genus level in the SHAM group is consistent with that in the no operation group. This figure shows the independent distribution of the gut microbiota genera in the OVX, no operation, and SHAM group. The percentages of the first and second principal components were 45% and 25.4%, respectively. The colors represent the different time points, and the node shape represents the group information of mice. Supplementary Figure 3: *α*-Diversity of the OVX and control group mice at four time points at the gene level. We calculated the gene Shannon index of the OVX and the control group based on gene relative abundance. The left panel represents the OVX group; the right panel shows the control group. The *p* values were derived by Wilcoxon rank-sum test. Supplementary Figure 4: *α*-diversity of the OVX and control group mice at four time points at the species level. The Shannon index of the OVX and the control group is based on the relative abundance at the species level. The *p* values were derived by Wilcoxon rank-sum test. Supplementary Figure 5: the probability density of four gut microbiota species with significant differences between the OVX (*n* = 50) and control (*n* = 50) groups. The blue curve represents the control group; the orange curve represents the OVX group; and the area under the curve is 1. The abscissa represents the relative abundance of each sample in the two groups, and the ordinate represents the probability density. Supplementary Figure 6: the probability density of four gut microbiota species with significant differences between the OVX (*n* = 50) and control (*n* = 15) groups. The blue curve represents the control group; the orange curve represents the OVX group; and the area under the curve is 1. The abscissa represents the relative abundance of each sample in the two groups, and the ordinate represents the probability density. Supplementary Table 1: the weight of the mice in this project at T1-T4 time points. Supplementary Table 2: the Wilcoxon rank-sum test results for the SMOTE algorithm construct the new control group (*n* = 50) and the original control group (*n* = 15) compared with the OVX group. Supplementary Table 3: the summary of data size and removal of host rates. Supplementary Table 4: the PCOA results of the two highest proportion principal components. Supplementary Table 5: the taxonomy of the gut microbiota and Kruskal–Wallis (KW) test for the gut microbiota species in OVX group mice at different time points. Supplementary Table 6: the Wilcoxon rank-sum test for significantly changed gut microbiota species between OVX and control group mice. Supplementary Table 7: the mice's weight is strongly associated with gut microbiota species. Supplementary Table 8: the results of the GMM classification..
