# Supplementary material for: Dynamic Alteration of the Gut Microbiota Associated with Obesity and Intestinal Inflammation in Ovariectomy C57BL/6 Mice
Source: Int J Endocrinol. 2022 Jan 22;2022:6600158. doi: 10.1155/2022/6600158 (PMC8800624; doi:10.1155/2022/6600158)

Supplementary Figures For Dynamic alteration of the gut microbiota associated with obesity and intestinal inflammation in ovariectomy C57BL/6 mice

Supplementary Figure 1. The timeline of different treatment and sample collection in this study. At the T1 time point, three days after the eight-week-old mice were acclimatized, we first collected the fecal samples. Then the mice underwent SHAM operation and OVX surgery. We collected mice fecal samples once a week since the T1 time point within one month.

Supplementary Figure 2. The gut microbiota at the genus level in the SHAM group is consistent with that in the no operation group.

This figure shows the independent distribution of the gut microbiota genera in the OVX, no operation, and SHAM group. The percentage of the first and second principal components were 45% and 25.4%, respectively. The colors represent the different time points, and the node shape represents the group information of mice.

Supplementary Figure 3. α-Diversity of the OVX and control group mice at four time points at the gene level. We calculated the gene Shannon index of the OVX and the control group based on gene relative abundance. The left panel represents the OVX group; the right panel shows the control group. The p-values were derived by Wilcoxon rank-sum test.

Supplementary Figure 4. α-Diversity of the OVX and control group mice at four time points at the species level. The Shannon index of the OVX and the control group is based on the relative abundance at the species level. The p-values were derived by Wilcoxon rank-sum test.

Supplementary Figure 5. The probability density of four gut microbiota species with significant differences between the OVX (n=50) and control (n = 50) groups. The blue curve represents the control group; the orange curve represents the OVX group, the area under the curve is 1. The abscissa represents the relative abundance of each sample in the two groups, and the ordinate represents the probability density.

Supplementary Figure 6. The probability density of four gut microbiota species with significant differences between the OVX (n=50) and control (n = 15) groups. The blue curve represents the control group; the orange curve represents the OVX group, the area under the curve is 1. The abscissa represents the relative abundance of each sample in the two groups, and the ordinate represents the probability density.

Supplementary Figure 1

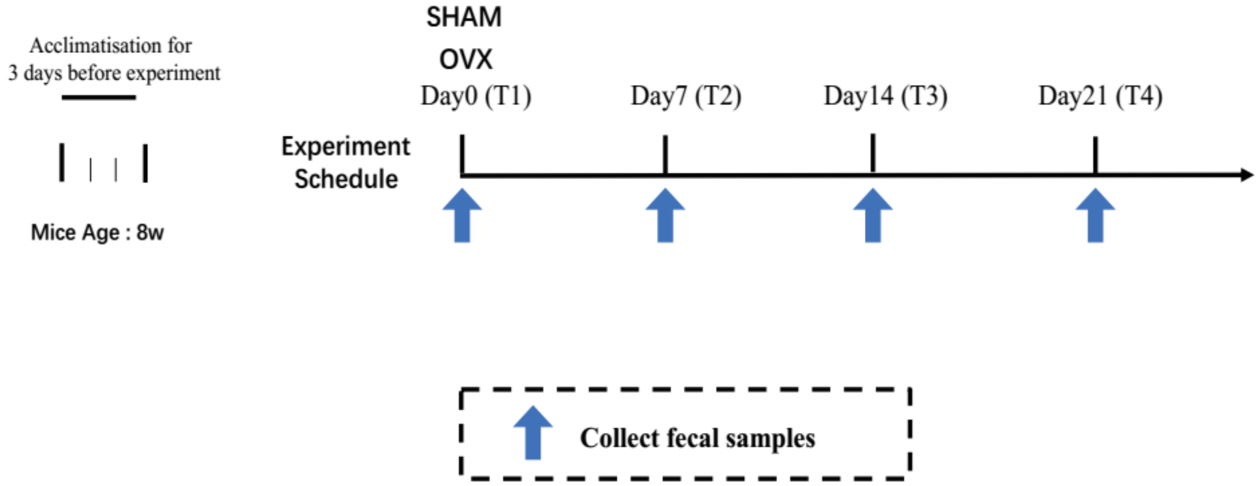

Supplementary Figure 2

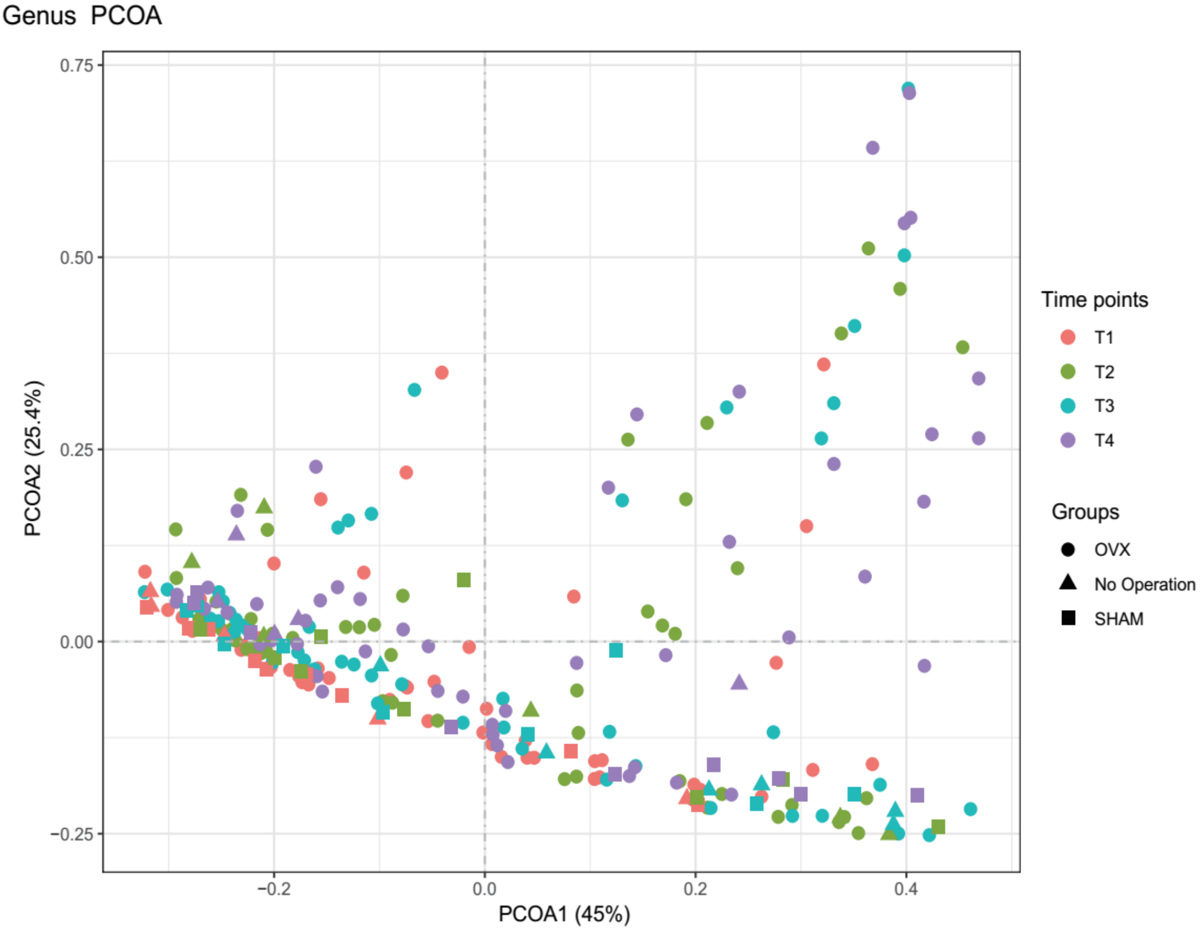

Supplementary Figure 3

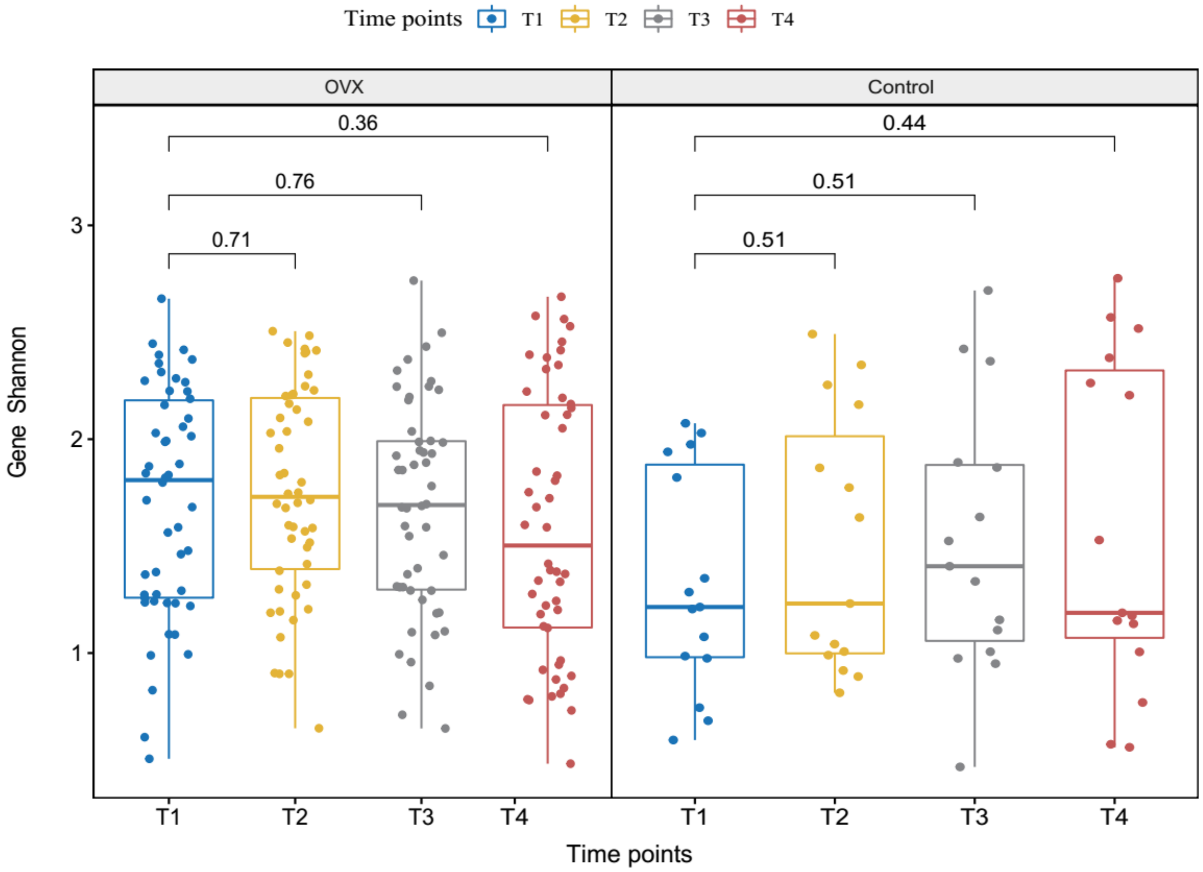

Supplementary Figure 4

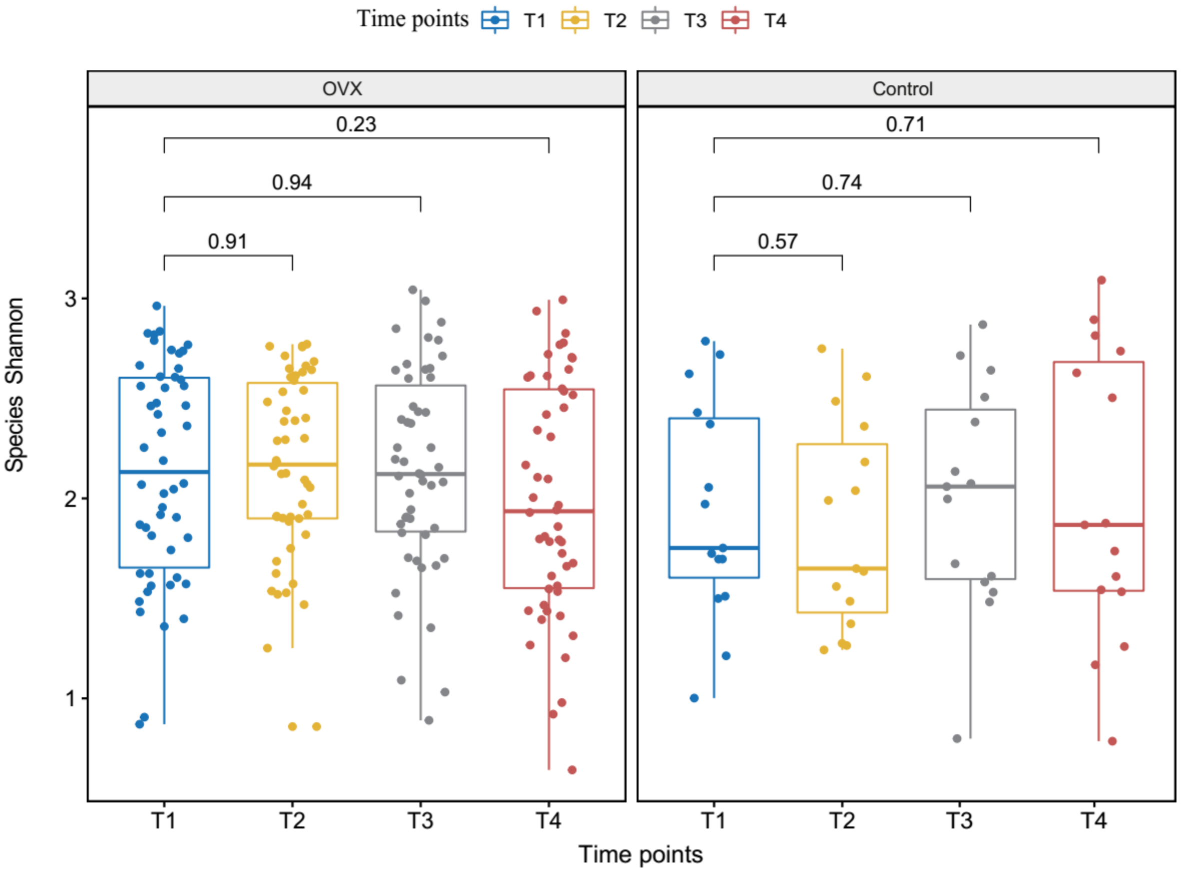

Supplementary Figure 5

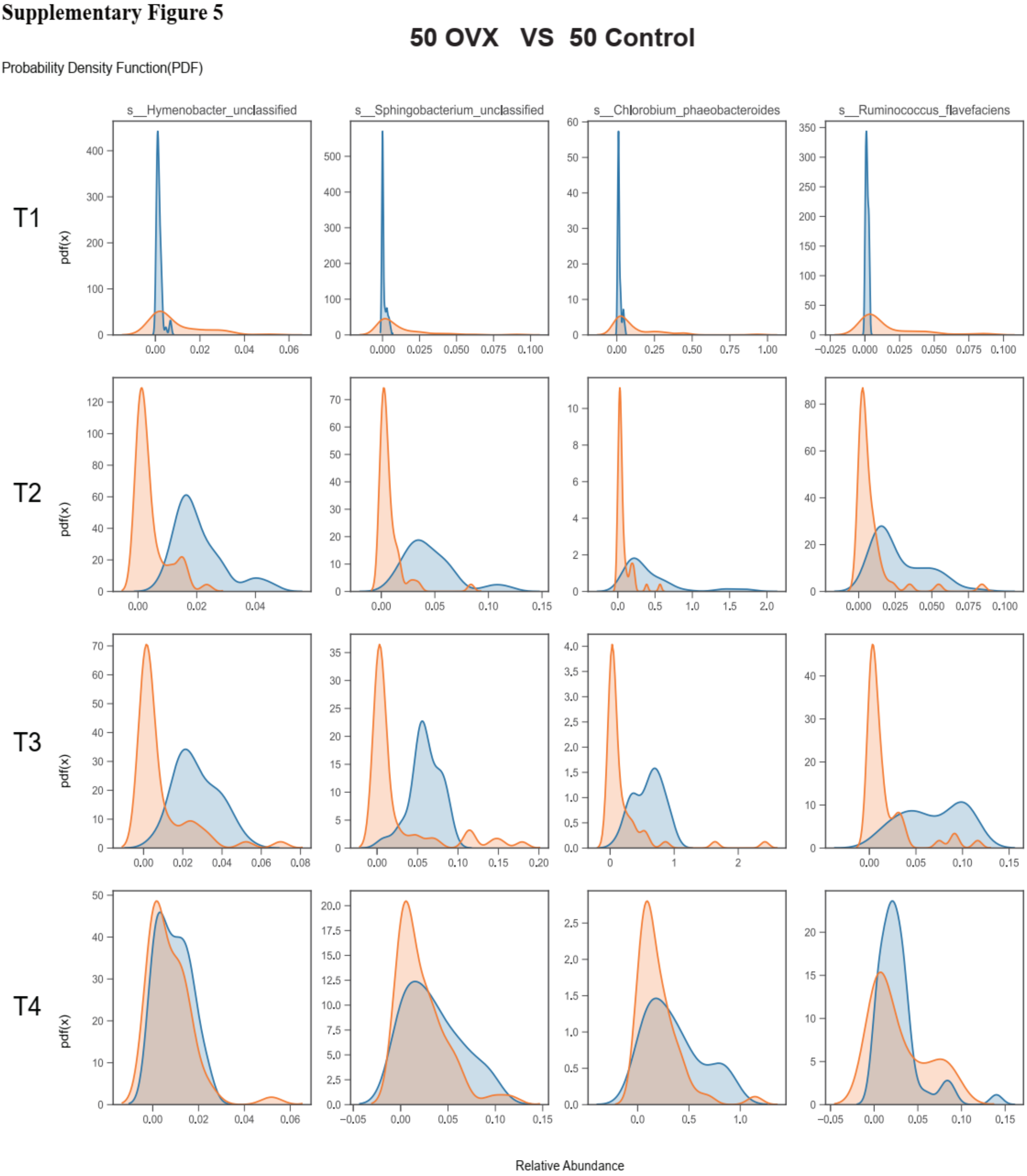

Supplementary Figure 6

Probability Density Function(PDF)

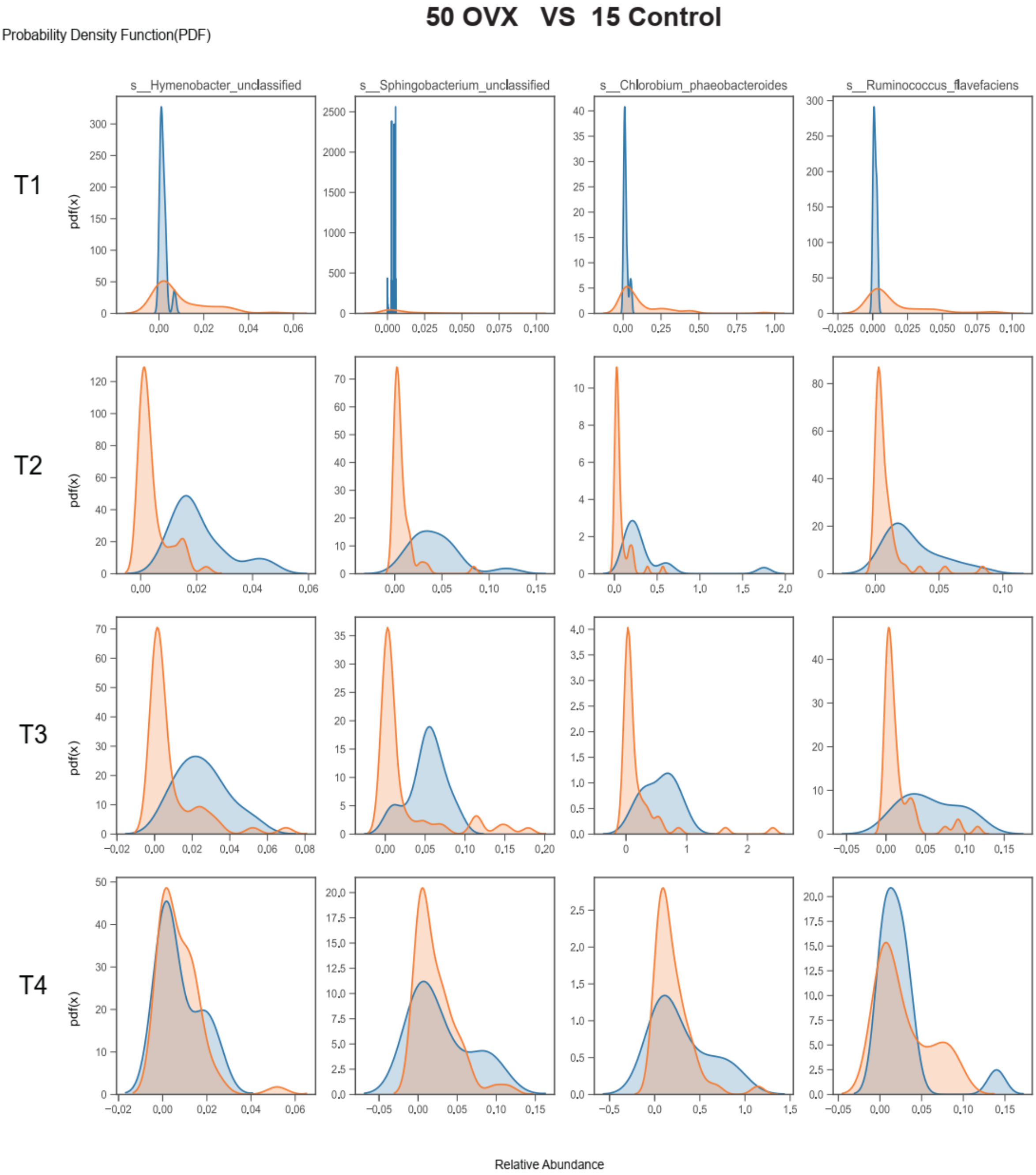

Supplement: Supplementary Materials — Supplementary Figure 1: the timeline of different treatment and sample collection in this study. At the T1 time point, three days after the eight-week-old mice were acclimatized, we first collected the fecal samples. Then, the mice underwent SHAM operation and OVX surgery. We collected mice fecal samples once a week since the T1 time point within one month. Supplementary Figure 2: the gut microbiota at the genus level in the SHAM group is consistent with that in the no operation group. This figure shows the independent distribution of the gut microbiota genera in the OVX, no operation, and SHAM group. The percentages of the first and second principal components were 45% and 25.4%, respectively. The colors represent the different time points, and the node shape represents the group information of mice. Supplementary Figure 3: α-Diversity of the OVX and control group mice at four time points at the gene level. We calculated the gene Shannon index of the OVX and the control group based on gene relative abundance. The left panel represents the OVX group; the right panel shows the control group. The p values were derived by Wilcoxon rank-sum test. Supplementary Figure 4: α-diversity of the OVX and control group mice at four time points at the species level. The Shannon index of the OVX and the control group is based on the relative abundance at the species level. The p values were derived by Wilcoxon rank-sum test. Supplementary Figure 5: the probability density of four gut microbiota species with significant differences between the OVX (n = 50) and control (n = 50) groups. The blue curve represents the control group; the orange curve represents the OVX group; and the area under the curve is 1. The abscissa represents the relative abundance of each sample in the two groups, and the ordinate represents the probability density. Supplementary Figure 6: the probability density of four gut microbiota species with significant differences between the OVX (n = 50) and control (n [file 6600158.f1.zip › 6600158.f1/Supplementary Figures For the paper.pdf]
