# Supplementary material for: Dynamic Alteration of the Gut Microbiota Associated with Obesity and Intestinal Inflammation in Ovariectomy C57BL/6 Mice
Source: Int J Endocrinol. 2022 Jan 22;2022:6600158. doi: 10.1155/2022/6600158 (PMC8800624; doi:10.1155/2022/6600158)
Supplement: Supplementary Materials — Supplementary Figure 1: the timeline of different treatment and sample collection in this study. At the T1 time point, three days after the eight-week-old mice were acclimatized, we first collected the fecal samples. Then, the mice underwent SHAM operation and OVX surgery. We collected mice fecal samples once a week since the T1 time point within one month. Supplementary Figure 2: the gut microbiota at the genus level in the SHAM group is consistent with that in the no operation group. This figure shows the independent distribution of the gut microbiota genera in the OVX, no operation, and SHAM group. The percentages of the first and second principal components were 45% and 25.4%, respectively. The colors represent the different time points, and the node shape represents the group information of mice. Supplementary Figure 3: α-Diversity of the OVX and control group mice at four time points at the gene level. We calculated the gene Shannon index of the OVX and the control group based on gene relative abundance. The left panel represents the OVX group; the right panel shows the control group. The p values were derived by Wilcoxon rank-sum test. Supplementary Figure 4: α-diversity of the OVX and control group mice at four time points at the species level. The Shannon index of the OVX and the control group is based on the relative abundance at the species level. The p values were derived by Wilcoxon rank-sum test. Supplementary Figure 5: the probability density of four gut microbiota species with significant differences between the OVX (n = 50) and control (n = 50) groups. The blue curve represents the control group; the orange curve represents the OVX group; and the area under the curve is 1. The abscissa represents the relative abundance of each sample in the two groups, and the ordinate represents the probability density. Supplementary Figure 6: the probability density of four gut microbiota species with significant differences between the OVX (n = 50) and control (n [file 6600158.f1.zip › 6600158.f1/Supplementary Tables For the paper.pdf]

Supplementary Table 1. The weight of the mice in this project at T1-T4 time points.

Supplementary Table 2. The Wilcoxon rank-sum test results for the SMOTE algorithm construct the new control group (n = 50) and the original control group (n = 15) compared with the OVX group.

Supplementary Table 3. The summary of data size and remove host rates.

Supplementary Table 4. The PCOA results of the two highest proportion principal components.

Supplementary Table 5. The taxonomic of the gut microbiota and KS-test for the gut microbiota species in OVX group mice at different time point .

Supplementary Table 6. The Wilcoxon rank-sum test for significantly changed gut microbiota species between OVX and control group mice.

Supplementary Table 7. The mice's weight is strongly associated with gut microbiota species.

Supplementary Table 8. The results of the GMM classification.

Supplementary Table 1 | The weight of the mice in the experiment at T1-T4 time points.

Sample_ID	group	T1	T2	T3	T4
20801	OVX	17.4	18.5	20.5	20.3
20865	OVX	20.3	22.1	22.5	24.2
20888	OVX	19.5	23.8	24.3	23.1
20884	OVX	19.4	23.2	23.5	22.1
20859	OVX	18.1	21	22.9	21.4
20880	OVX	18.2	23	22.8	22.5
20863	OVX	19.6	20.7	21.3	21.8
20879	OVX	18.5	19.8	20.9	20.5
20842	OVX	17.9	21.4	23.2	22.2
20846	OVX	16.1	19.3	20.7	20.7
20808	OVX	18.7	18.6	22.1	22.1
20867	OVX	19.8	23.8	24.8	24.6
20877	OVX	19.5	21.5	22.5	23.4
20847	OVX	18.5	22.4	24.4	22.7
20875	OVX	18.4	21.5	22.3	21.5
20887	OVX	18.2	19.4	19.8	21.1
20850	OVX	17.8	19.9	21.1	20.5
20898	OVX	17.7	22.1	23.2	22.1
20876	OVX	14.7	20.1	23.2	21.8
20862	OVX	16.7	20.2	20.4	19.8
20864	OVX	20.2	21.4	22.3	22.8
20874	OVX	19.1	23.8	23.7	22.4
20845	OVX	18.5	22.2	24.5	22.6
20855	OVX	18.5	22.3	22.4	21.6
20899	OVX	18.7	23.2	22.5	21.3
20857	OVX	17.7	21.2	22.6	22.1
20872	OVX	18.4	20.1	21.5	23.5
20853	OVX	18.3	18.8	20	20.6
20868	OVX	17.8	21.7	21.7	20.9
20866	OVX	15.2	18.6	19.3	18.6
20800	OVX	18.5	18.6	21.7	23.5
20885	OVX	18.9	21.3	22.6	21.9
20871	OVX	20.1	20.8	22.1	23.1
20861	OVX	18.7	19.9	20.6	21.1
20881	OVX	17.6	21.5	23.6	21.7
20878	OVX	18.2	21.8	22.9	22.5
20896	OVX	18.3	21.9	21.3	20.6
20849	OVX	15.7	20	22.2	21.4
20882	OVX	17	18.5	20.2	22.6
20851	OVX	17.1	18.8	16.6	17
20803	OVX	17.1	17.4	21.1	21.1
20854	OVX	18.9	22.3	21.9	22.2
20895	OVX	18.3	22.4	23.4	22.5
20860	OVX	19	23.5	22.5	24
20832	OVX	13.6	17.6	18.4	21.8
20873	OVX	18.9	22.2	23.5	22.1
20869	OVX	18.2	21.4	22.3	19.1
20843	OVX	15.1	19.1	19.8	22.8
20897	OVX	17.6	19.3	19.9	20.5
20870	OVX	17.4	20.7	21.6	20.8
20804	SHAM	18.7	19.3	20.9	20.8
20806	SHAM	18.6	18.6	19.4	19.7
20809	SHAM	17.3	17.9	18.8	19.7
20811	SHAM	18.4	18.1	19.1	19.4
20813	SHAM	18.8	18.5	20	20.1
20814	SHAM	17	17.2	17.8	19
20816	SHAM	18	18.4	19.5	19.7
20817	SHAM	18.6	18.4	19.9	19.2
20818	SHAM	19	18.6	20.1	20.2
20802	Control	19.2	19.8	20.1	20.6
20810	Control	18.3	18.6	18.7	19.5
20812	Control	18.8	20.8	20.2	20.7
20815	Control	18	18.9	19.4	19.2
20819	Control	18.3	18.3	18.9	19.6
20820	Control	18.4	18.7	19.2	19.9

Supplementary Table 2 | The Wilcoxon rank-sum test results for the SMOTE algorithm construct the new control group (n = 50) and the original control group (n = 15) compared with the OVX group (n = 50).

50 Control mice VS 50 OVX mice

Species	p-value(T1)	p-value(T2)	p-value(T3)	p-value(T4)
s__Acetobacter_unclassified	1	1	1	0.863165918
s__Acinetobacter_pittii_calcoaceticus_no	0.863165918	1	1	1
s__Acinetobacter_unclassified	1	0.863165918	0.863165918	0.605130686
s__Actinobacillus_unclassified	0.950527491	0.198548687	0.994499571	0.301102046
s__Actinoplanes_unclassified	1	1	0.863165918	1
s__Adlercreutzia_equolifaciens	0.117607036	0.057986434	0.709694607	0.501489839
s__Aerococcus_viridans	0.863165918	0.252468525	0.863165918	0.025059035
s__Aggregatibacter_segnis	0.863165918	1	1	1
s__Aggregatibacter_unclassified	0.73032669	0.01075027	0.863165918	0.190254781
s__Agrobacterium_tumefaciens	1	1	1	1
s__Agrobacterium_unclassified	1	1	1	1
s__Akkermansia_muciniphila	3.29E-08	0.053573446	0.000427107	0.634306952
s__Alicyclobacillus_unclassified	0.505888291	6.32E-06	4.41E-10	0.019438927
s__Alistipes_indistinctus	1.23E-09	0.099430068	0.007325001	0.021111596
s__Alistipes_onderdonkii	1	0.863165918	1	1
s__Alistipes_putredinis	0.059834274	1.92E-09	2.65E-08	0.438011935
s__Alistipes_senegalensis	4.90E-05	0.000197133	0.032872486	0.014392856
s__Alistipes_shahii	1	1	0.73032669	0.863165918
s__Alistipes_unclassified	0.06876381	0.066690209	0.033729012	0.230324003
s__Alloprevotella_rava	0.227656175	1	0.73032669	1
s__Alloprevotella_tannerae	0.227656175	1	0.73032669	1
s__Alloprevotella_unclassified	5.04E-05	3.05E-09	2.59E-10	0.764267802
s__alpha_proteobacterium_SCGC_AAA02	0.863165918	1	1	1
s__Anaerococcus_prevotii	0.490582916	0.00089112	6.63E-06	0.490582916
s__Anaerococcus_vaginalis	0.863165918	0.120875452	1	0.490582916
s__Anaerostipes_unclassified	0.73032669	0.219785031	0.015828899	0.605130686
s__Anaerotruncus_colihominis	0.486256121	0.546369502	0.176634995	0.059367796
s__Anaerotruncus_sp_G3_2012	0.243996504	0.863165918	2.17E-05	0.576571676
s__Anaerotruncus_unclassified	0.120875452	0.120875452	0.381293685	0.766898469
s__Arcobacter_unclassified	0.490582916	0.086059386	2.69E-05	0.37014778
s__Asticcacaulis_unclassified	1	1	0.863165918	1
s__Bacillus_cereus_thuringiensis	1	1	0.003391001	0.863165918
s__Bacteriovorax_unclassified	0.271522017	2.75E-08	1.41E-10	0.192597993
s__Bacteroidales_bacterium_ph8	0.000130205	5.20E-05	0.000467684	0.172260488
s__Bacteroides_caccae	0.073070008	0.003466992	0.002590207	0.772167842
s__Bacteroides_cellulosilyticus	0.057986434	0.185631467	0.809335247	0.442094633
s__Bacteroides_coprocola	0.490582916	1.55E-06	0.000179111	0.830774448
s__Bacteroides_coprophilus	1	0.227656175	1	1
s__Bacteroides_dorei	2.17E-05	0.00811549	0.023325139	0.012822229
s__Bacteroides_eggerthii	1	0.490582916	1	1
s__Bacteroides_finegoldii	0.025059035	0.000280098	0.002531981	0.467043425
s__Bacteroides_fluxus	0.73032669	0.177741447	0.301102046	0.324223294
s__Bacteroides_fragilis	1	0.490582916	1	1
s__Bacteroides_helcogenes	1	0.863165918	1	0.863165918
s__Bacteroides_intestinalis	0.388837498	0.817359111	0.817359111	0.388837498
s__Bacteroides_massiliensis	4.90E-05	4.41E-08	1.78E-08	0.12591082
s__Bacteroides_oleiciplenus	0.047482239	0.383171165	0.793348306	0.448259326
s__Bacteroides_ovatus	0.01075027	0.006203123	0.110506812	0.014392856
s__Bacteroides_pectinophilus	1	0.73032669	1	1
s__Bacteroides_plebeius	0.863165918	0.057986434	1	1
s__Bacteroides_pyogenes	1	0.120875452	1	1
s__Bacteroides_salanitronis	1	0.863165918	1	1
s__Bacteroides_salyersiae	1	1	0.863165918	1
s__Bacteroides_stercoris	0.388837498	0.798668003	0.863165918	0.73032669
s__Bacteroides_thetaiotaomicron	0.025059035	1.28E-05	0.000319967	0.310873187
s__Bacteroides_uniformis	0.252468525	1.26E-05	0.007325001	0.157587205
s__Bacteroides_vulgatus	0.841542541	0.022093987	0.41200831	0.002107542
s__Bacteroides_xylanisolvens	0.863165918	0.00811549	0.562534608	0.777447743
s__Bacteroidetes_bacterium_oral_taxon_	1	1	0.863165918	1
s__Bacteroidetes_oral_taxon_274	1	1	0.863165918	1
s__Bartonella_unclassified	0.011293657	1.02E-06	2.25E-07	0.681672237
s__Bifidobacterium_animalis	1	1	1	1
s__Bifidobacterium_dentium	1	1	1	1
s__Bifidobacterium_pseudolongum	0.396471473	0.732919839	0.007325001	0.793348306
s__Bilophila_wadsworthia	1	1	0.863165918	1
s__Blattabacterium_unclassified	0.057986434	2.14E-12	1.94E-07	0.433951007
s__Blautia_hydrogenotrophica	0.301102046	0.119232468	0.002391614	0.950527491
s__Blautia_producta	0.120875452	0.006673385	2.92E-08	0.227656175
s__Borrelia_bavariensis	0.863165918	0.219785031	0.490582916	1
s__Borrelia_duttonii	1	1	0.863165918	1
s__Borrelia_recurrentis	0.301102046	1	0.057986434	0.605130686
s__Brachyspira_unclassified	0.025059035	1.97E-08	3.90E-11	0.273027418
s__Brevibacterium_unclassified	0.782737942	0.246798039	0.030946059	0.057986434
s__Brevundimonas_unclassified	1	1	1	0.605130686
s__Brucella_microti	0.863165918	0.293117369	0.73032669	0.042684605
s__Brucella_pinnipedialis	0.863165918	0.227656175	0.863165918	1
s__Buchnera_aphidicola	0.863165918	0.863165918	0.408090626	0.301102046
s__Bulleidia_extructa	1	1	0.863165918	1
s__Burkholderia_unclassified	1	0.863165918	1	1
s__Burkholderiales_bacterium_1_1_47	1.10E-09	0.056186268	0.388837498	0.291537646
s__Butyricicoccus_pullicaecorum	0.227656175	0.042684605	9.96E-05	0.809335247
s__Butyricimonas_synergistica	0.000869349	0.033729012	0.006535857	0.24121738
s__Butyrivibrio_crossotus	1	0.120875452	1	1
s__Butyrivibrio_fibrisolvens	1	0.227656175	1	0.863165918
s__Butyrivibrio_unclassified	3.49E-06	3.64E-05	5.53E-11	0.285276059
s__Campylobacter_rectus	1	1	0.863165918	1
s__Campylobacter_showae	1	1	0.863165918	1
s__Candidatus_Arthromitus_sp_SFB_1	0.863165918	0.719985155	1	0.906705578
s__Candidatus_Arthromitus_sp_SFB_5	1	1	1	0.863165918
s__Candidatus_Arthromitus_unclassified	0.000319967	0.243996504	0.238460619	0.002590207
s__Candidatus_Liberibacter_unclassified	0.388837498	0.0758717	0.040275575	0.025283429
s__Candidatus_Moranella_endobia	1	1	1	0.863165918
s__Candidatus_Pelagibacter_ubique	0.863165918	0.117607036	0.230324003	0.605130686
s__Candidatus_Phytoplasma_unclassified	0.009732748	0.000902194	0.037664555	0.400322151
s__Candidatus_Portiera_aleyrodidarum	1	0.73032669	1	1
s__Candidatus_Sulcia_muelleri	0.490582916	0.001347689	0.003870711	0.301102046
s__Candidatus_Uzinura_diaspidicola	0.863165918	0.252468525	0.73032669	0.863165918
s__Candidatus_Zinderia_insecticola	0.127625113	2.21E-10	9.33E-09	0.4523961
s__Capnocytophaga_gingivalis	0.863165918	1	1	1
s__Capnocytophaga_unclassified	0.003172037	1.94E-12	5.28E-13	0.006813508
s__Caulobacter_unclassified	0.089257663	0.000721126	1.15E-10	0.304336109
s__Cellulophaga_unclassified	0.01075027	6.72E-09	3.13E-07	0.785386832
s__Cellvibrio_unclassified	0.73032669	0.73032669	0.016283603	0.510306962
s__Chlamydophila_unclassified	0.764267802	0.025967075	0.000538778	0.460734016
s__Chlorobium_phaeobacteroides	0.000119694	1.39E-13	5.17E-12	0.001728873
s__Clostridiales_bacterium_1_7_47FAA	1	0.863165918	0.167967537	0.863165918
s__Clostridium_asparagiforme	0.301102046	0.047482239	0.000197133	0.167967537
s__Clostridium_citroniae	1	0.490582916	1	1
s__Clostridium_clostridioforme	0.009539862	2.35E-08	1.07E-11	0.46283184
s__Clostridium_hathewayi	0.000389836	7.71E-08	2.81E-12	0.967006574
s__Clostridium_hylemonae	0.490582916	0.751155802	0.738115265	0.73032669
s__Clostridium_leptum	0.863165918	0.863165918	1	1
s__Clostridium_nexile	1	0.490582916	1	1
s__Clostridium_pasteurianum	0.863165918	0.023962747	0.000596673	0.863165918
s__Clostridium_phytofermentans	1	0.863165918	1	1
s__Clostridium_ramosum	1	0.120875452	1	1
s__Clostridium_scindens	0.001138392	2.81E-05	3.90E-11	0.801331377
s__Clostridium_sp_ASF356	0.011183068	0.160648944	0.000936186	0.011405216
s__Clostridium_sp_ASF502	0.106711087	0.544079129	0.000328553	0.994499571
s__Clostridium_symbiosum	0.167967537	5.28E-06	3.31E-07	0.782737942
s__Collinsella_unclassified	1.45E-05	5.19E-06	9.68E-07	0.065169032
s__Comamonas_unclassified	0.674103422	0.619643946	0.000205366	0.167967537
s__Coprobacillus_unclassified	1.05E-07	0.331036319	0.106711087	0.812007686
s__Coprococcus_catus	0.863165918	0.095255313	0.605130686	0.73032669
s__Corynebacterium_crenatum	1	0.863165918	1	1
s__Corynebacterium_mastitidis	1	1	1	1
s__Crenarchaeota_archaeon_SCGC_AAA	0.490582916	0.001007757	0.046336623	0.674103422
s__Crenarchaeota_archaeon_SCGC_AAA	1	0.73032669	0.04163764	1
s__Cyanobium_unclassified	1	0.863165918	1	1
s__Cytophaga_unclassified	0.0386263	4.41E-10	0.008198594	0.140137966
s__Deinococcus_unclassified	0.001032718	1.29E-08	8.75E-11	0.03580208
s__Desulfobulbus_sp_oral_taxon_041	1	1	0.863165918	0.863165918
s__Desulfovibrio_desulfuricans	0.093895345	0.56719526	0.007477337	2.86E-06
s__Desulfovibrio_termitidis	0.227656175	2.41E-06	0.000319967	0.11283494
s__Dorea_formicigenerans	0.120875452	5.46E-06	0.000272705	0.508095107
s__Dorea_longicatena	0.227656175	0.000427107	5.79E-07	0.694358086
s__Dorea_sp_5_2	0.000627745	0.138294728	3.38E-06	0.274538518
s__Dorea_unclassified	0.863165918	0.002835561	0.874017234	0.368310003
s__Dysgonomonas_unclassified	0.279106044	5.72E-14	1.65E-10	0.010644425
s__Echinicola_unclassified	0.009732748	2.53E-10	2.54E-06	0.375695207
s__Eggerthella_unclassified	1	0.005073372	0.868588482	0.73032669
s__Ehrlichia_unclassified	0.057986434	8.73E-06	3.18E-05	0.301102046
s__Elizabethkingia_unclassified	1	1	1	1
s__Enhydrobacter_aerosaccus	1	1	1	1
s__Enterobacter_cloacae	1	1	1	1
s__Enterococcus_faecalis	0.82540216	0.388837498	0.66406287	0.000324233
s__Enterococcus_gallinarum	0.135564973	0.18109176	0.388837498	0.388837498
s__Enterococcus_gilvus	1	1	1	1
s__Enterorhabdus_caecimuris	0.661562037	0.782737942	0.249622031	0.279106044
s__Erysipelotrichaceae_bacterium_2_2_4	0.863165918	1	1	1
s__Erysipelotrichaceae_bacterium_21_3	0.002966034	0.000191817	6.01E-08	0.950527491
s__Erysipelotrichaceae_bacterium_5_2_5	0.863165918	1	0.73032669	0.863165918
s__Erysipelotrichaceae_bacterium_6_1_4	0.00354453	0.011183068	3.31E-10	0.475530006
s__Escherichia_albertii	1	1	1	0.863165918
s__Escherichia_coli	0.136470229	1.67E-12	2.00E-13	3.16E-11
s__Escherichia_unclassified	0.025737438	0.000827279	0.03148632	0.994499571
s__Eubacteriaceae_bacterium_CM2	0.863165918	0.988999404	0.025059035	0.73032669
s__Eubacterium_cellulosolvens	1	0.063676587	0.006956271	0.605130686
s__Eubacterium_eligens	0.605130686	0.956018517	0.222386748	0.863165918
s__Eubacterium_hallii	0.73032669	0.0386263	0.006400884	0.73032669
s__Eubacterium_plexicaudatum	0.115999045	0.431928731	7.98E-07	0.983499759
s__Eubacterium_ramulus	1	0.053573446	1	1
s__Eubacterium_rectale	0.994499571	0.034604552	0.095941189	0.388837498
s__Eubacterium_saphenum	1	1	0.863165918	1
s__Eubacterium_sp_14_2	0.619643946	0.863165918	0.082954339	0.227656175
s__Eubacterium_ventriosum	0.863165918	0.057986434	0.222386748	0.73032669
s__Exiguobacterium_unclassified	0.490582916	0.160648944	0.001194897	0.227656175
s__Facklamia_unclassified	1	1	0.863165918	1
s__Faecalibacterium_prausnitzii	0.003391001	8.12E-07	3.09E-10	0.041984186
s__Fervidobacterium_unclassified	0.605130686	0.863165918	3.27E-06	0.490582916
s__Filifactor_alocis	1	1	0.863165918	1
s__Firmicutes_bacterium_M10_2	0.73032669	0.252468525	0.082344357	0.004913074
s__Flavonifractor_plautii	0.490582916	0.087327407	0.227656175	0.490582916
s__Fretibacterium_fastidiosum	1	1	0.863165918	1
s__Fusobacterium_nucleatum	1	1	0.863165918	1
s__Fusobacterium_periodonticum	1	1	1	1
s__Gardnerella_vaginalis	0.490582916	0.120875452	0.605130686	0.73032669
s__Gemella_unclassified	0.863165918	0.025059035	1	0.73032669
s__Geobacillus_unclassified	0.167967537	4.83E-09	2.61E-05	0.510306962
s__Gillisia_unclassified	0.00441129	2.61E-12	6.26E-10	0.134664362
s__Granulicatella_unclassified	0.227656175	0.11205464	0.100853655	0.782737942
s__Granulicella_unclassified	0.051888553	0.010539508	4.48E-05	0.0799407
s__Haemophilus_haemolyticus	1	0.863165918	1	1
s__Haemophilus_influenzae	1	0.863165918	1	1
s__Haemophilus_parainfluenzae	0.392643238	0.605130686	0.605130686	0.028362563
s__Halomonas_unclassified	0.490582916	0.047869364	7.19E-06	0.884892298
s__Helicobacter_canadensis	0.015095844	1.61E-08	1.55E-12	0.022497939
s__Helicobacter_cinaedi	0.000405421	1.07E-12	3.46E-09	0.01269846
s__Helicobacter_hepaticus	0.836154656	0.004808769	0.01075027	0.000265495
s__Helicobacter_pullorum	0.388837498	0.165851421	0.000432695	0.605130686
s__Helicobacter_winghamensis	7.27E-05	5.59E-07	8.11E-13	0.026431668
s__Hippea_unclassified	0.025059035	2.02E-11	0.000184095	0.084185287
s__Hymenobacter_unclassified	0.000492457	3.47E-15	6.35E-11	0.074180357
s__Johnsonella_ignava	1	0.227656175	1	0.863165918
s__Kingella_unclassified	0.227656175	0.73032669	0.258229189	0.666567448
s__Klebsiella_pneumoniae	1	1	1	1
s__Klebsiella_unclassified	0.73032669	0.73032669	0.388837498	0.227656175
s__Lachnobacterium_bovis	1	0.289963656	1	0.863165918
s__Lachnospiraceae_bacterium_1_1_57F	0.010964772	0.017066977	0.017227561	0.186779432
s__Lachnospiraceae_bacterium_1_4_56F	0.167967537	0.007554566	0.000244922	0.91763972
s__Lachnospiraceae_bacterium_2_1_46F	1	0.132877024	0.057986434	0.605130686
s__Lachnospiraceae_bacterium_2_1_58F	1	1	1	0.863165918
s__Lachnospiraceae_bacterium_3_1_46F	0.026431668	0.285276059	2.17E-08	0.010332428
s__Lachnospiraceae_bacterium_3_1_57F	0.167967537	0.011861295	1.98E-07	0.868588482
s__Lachnospiraceae_bacterium_4_1_37F	0.301102046	0.007790561	0.000567038	0.553269154
s__Lachnospiraceae_bacterium_5_1_57F	0.301102046	0.227656175	0.000295452	0.714833401
s__Lachnospiraceae_bacterium_5_1_63F	1	0.314176217	0.167967537	1
s__Lachnospiraceae_bacterium_6_1_63F	1	0.863165918	1	1
s__Lachnospiraceae_bacterium_7_1_58F	0.355605107	4.09E-09	7.26E-12	0.110506812
s__Lachnospiraceae_bacterium_8_1_57F	0.032591147	0.442094633	0.004606148	0.233013983
s__Lachnospiraceae_bacterium_9_1_43B	0.863165918	0.060776353	0.000238403	0.490582916
s__Lactobacillus_amylovorus	1	0.289963656	1	1
s__Lactobacillus_animalis	0.586020558	0.037348535	0.004606148	0.983499759
s__Lactobacillus_casei_paracasei	1	1	1	1
s__Lactobacillus_crispatus	0.863165918	1	0.73032669	1
s__Lactobacillus_delbrueckii	1	1	0.863165918	1
s__Lactobacillus_gasseri	8.60E-09	2.86E-06	0.001649387	4.51E-07
s__Lactobacillus_hominis	2.16E-10	3.32E-09	1.86E-05	8.32E-13
s__Lactobacillus_iners	1	0.167967537	0.301102046	0.863165918
s__Lactobacillus_jensenii	0.863165918	0.73032669	0.868588482	1
s__Lactobacillus_johnsonii	0.000137689	7.19E-06	2.88E-11	0.000913396
s__Lactobacillus_murinus	5.19E-06	0.264080354	0.002310851	0.002059345
s__Lactobacillus_reuteri	0.000171872	0.252468525	0.027382434	0.00058168
s__Lactobacillus_rhamnosus	1	1	1	1
s__Lactobacillus_sp_7_1_47FAA	1	1	0.73032669	1
s__Lactobacillus_sp_ASF360	1	1	0.863165918	1
s__Lactobacillus_vaginalis	9.01E-06	0.008981494	2.69E-05	0.001347689
s__Lactococcus_lactis	0.388837498	1	1	1
s__Leifsonia_unclassified	1	1	1	0.863165918
s__Leptotrichia_unclassified	0.863165918	1	1	1
s__Leuconostoc_pseudomesenteroides	1	1	1	1
s__Leuconostoc_unclassified	0.207103903	0.00058168	0.009732748	0.605130686
s__Listeria_unclassified	0.863165918	0.103749525	1	0.490582916
s__Lysinibacillus_sphaericus	1	0.000567038	0.863165918	1
s__Marinobacter_unclassified	0.863165918	0.863165918	0.863165918	1
s__Marvinbryantia_formatexigens	0.184488728	4.55E-06	7.14E-08	0.583651611
s__Megamonas_unclassified	1	0.793348306	1	1
s__Megasphaera_unclassified	0.003391001	2.38E-05	3.08E-07	0.544079129
s__Meiothermus_unclassified	0.863165918	0.227656175	1	1
s__Methanocaldococcus_unclassified	0.167967537	1	1	1
s__Methanothermococcus_unclassified	0.863165918	0.090563487	1	1
s__Methylotenera_versatilis	0.863165918	0.490582916	0.863165918	1
s__Micrococcus_luteus	1	1	0.863165918	1
s__Microcoleus_unclassified	0.863165918	1	1	1
s__Mitsuokella_unclassified	1.80E-07	1.29E-11	2.07E-11	0.516972617
s__Mobiluncus_unclassified	1	0.043038504	1	1
s__Mucispirillum_schaedleri	0.21720523	0.246798039	0.000299412	0.09457337
s__Mycoplasma_capricolum	1	1	0.863165918	0.863165918
s__Mycoplasma_hyorhinis	1	1	1	1
s__Myroides_unclassified	0.025059035	1.29E-07	5.35E-05	0.301102046
s__Neisseria_elongata	0.863165918	1	1	1
s__Neisseria_flavescens	0.312521827	1	0.863165918	0.73032669
s__Neisseria_macacae	0.863165918	1	1	1
s__Neisseria_meningitidis	0.863165918	0.167967537	1	0.73032669
s__Neisseria_sicca	0.312521827	1	1	1
s__Neisseria_subflava	0.227656175	1	1	0.863165918
s__Neisseria_unclassified	0.157587205	1.23E-09	2.99E-05	0.288395396
s__Niabella_unclassified	0.057986434	4.06E-12	4.78E-06	0.291537646
s__Nitrosospira_unclassified	1	0.293117369	1	1
s__Ochrobactrum_sp_CDB2	1	1	1	1
s__Odoribacter_laneus	0.003391001	3.90E-07	8.32E-06	0.03490068
s__Odoribacter_unclassified	0.057986434	0.836154656	0.111278602	0.227656175
s__Olsenella_unclassified	7.99E-11	7.92E-09	9.67E-10	0.001194897
s__Oribacterium_sp_oral_taxon_078	0.73032669	0.863165918	1	0.73032669
s__Oscillibacter_sp_1_3	0.753772596	0.058904339	0.022497939	0.876733852
s__Oscillibacter_unclassified	0.331036319	0.000827279	0.043394879	0.145781141
s__Oscillibacter_valericigenes	0.0386263	0.000158211	1.58E-09	0.101571514
s__Oxalobacter_formigenes	0.863165918	1	0.057986434	0.863165918
s__Pantoea_dispersa	1	0.863165918	1	1
s__Pantoea_unclassified	0.874017234	0.015828899	0.167967537	0.120875452
s__Parabacteroides_distasonis	2.64E-09	0.086059386	0.011745784	0.089908667
s__Parabacteroides_goldsteinii	3.32E-06	0.448259326	0.024615415	0.021894387
s__Parabacteroides_johnsonii	0.000400163	5.20E-05	2.50E-11	0.22633055
s__Parabacteroides_merdae	0.000416132	0.0001672	1.54E-05	0.978000898
s__Parabacteroides_unclassified	3.77E-08	0.024178615	0.00151833	0.096631012
s__Paraprevotella_unclassified	0.025059035	0.008891325	0.000328553	0.024178615
s__Parasutterella_excrementihominis	2.21E-10	0.071430016	0.029372768	0.155570742
s__Pasteurella_pneumotropica	0.841542541	0.000869349	0.967006574	0.12170356
s__Pediococcus_pentosaceus	0.73032669	1	1	1
s__Pediococcus_unclassified	0.050248222	8.49E-10	8.26E-07	0.047097749
s__Pedobacter_unclassified	1.34E-08	7.63E-11	1.19E-07	0.218492395
s__Peptostreptococcaceae_noname_unc	1.72E-05	0.000473766	2.09E-09	0.967006574
s__Peptostreptococcus_stomatis	1	1	0.863165918	1
s__Peptostreptococcus_unclassified	0.339681856	2.41E-06	1.10E-09	0.4523961
s__Porphyromonas_asaccharolytica	1	1	0.863165918	1
s__Porphyromonas_endodontalis	1	1	0.73032669	1
s__Porphyromonas_gingivalis	1	1	0.73032669	1
s__Prevotella_amnii	0.863165918	1	1	0.863165918
s__Prevotella_bivia	1	0.605130686	1	1
s__Prevotella_copri	0.73032669	0.12591082	0.279106044	1
s__Prevotella_disiens	1	1	0.863165918	1
s__Prevotella_histicola	0.863165918	1	1	0.167967537
s__Prevotella_intermedia	0.227656175	1	0.73032669	1
s__Prevotella_melaninogenica	0.230324003	0.605130686	0.490582916	0.419910272
s__Prevotella_nanceiensis	0.312521827	0.490582916	0.863165918	0.291537646
s__Prevotella_pallens	1	1	1	1
s__Prevotella_pleuritidis	1	1	0.73032669	1
s__Prevotella_salivae	1	1	1	1
s__Prevotella_stercorea	1	1	0.863165918	1
s__Prevotella_timonensis	0.863165918	1	1	1
s__Prevotella_veroralis	0.863165918	1	1	1
s__Propionibacterium_acnes	1	1	0.863165918	1
s__Proteus_mirabilis	3.54E-09	0.000295452	0.015828899	0.025059035
s__Proteus_penneri	1	1	1	0.73032669
s__Proteus_unclassified	0.73032669	0.863165918	0.73032669	1
s__Providencia_unclassified	0.863165918	1	0.863165918	0.73032669
s__Pseudoflavonifractor_capillosus	0.868588482	2.76E-06	1.71E-08	0.408090626
s__Pseudomonas_aeruginosa	1	1	1	0.007790561
s__Pseudomonas_stutzeri	0.863165918	1	1	1
s__Pseudomonas_unclassified	0.087327407	0.214647282	0.219785031	0.297890956
s__Pusillimonas_unclassified	0.227656175	0.021304973	0.164800913	0.605130686
s__Rhodopseudomonas_palustris	1	0.863165918	1	1
s__Rhodospirillum_unclassified	0.863165918	0.73032669	0.086691525	0.605130686
s__Rickettsia_japonica	1	0.863165918	0.863165918	1
s__Riemerella_unclassified	0.863165918	0.002391614	0.047869364	0.490582916
s__Roseburia_hominis	0.084806282	0.009256922	1.03E-10	0.634306952
s__Roseburia_intestinalis	0.084806282	0.000467684	6.26E-10	0.530437956
s__Roseburia_inulinivorans	0.73032669	0.595540657	1	0.73032669
s__Roseburia_unclassified	0.000531921	0.001224112	5.92E-11	0.243996504
s__Rothia_mucilaginosa	1	1	0.863165918	1
s__Rothia_unclassified	1	1	1	1
s__Ruminococcaceae_bacterium_D16	0.003466992	8.89E-05	1.15E-10	0.569532503
s__Ruminococcus_albus	0.015532002	3.25E-07	2.12E-11	0.464934981
s__Ruminococcus_bromii	1	0.490582916	1	1
s__Ruminococcus_flavefaciens	2.09E-07	2.04E-12	1.18E-12	0.258229189
s__Ruminococcus_gnavus	0.057986434	0.030946059	9.88E-08	0.988999404
s__Ruminococcus_lactaris	0.73032669	0.73032669	0.057986434	0.605130686
s__Ruminococcus_obeum	0.301102046	0.004507766	0.000133898	0.950527491
s__Ruminococcus_torques	0.000449872	4.41E-08	1.64E-11	0.435978745
s__Saccharomonospora_unclassified	0.863165918	0.00105825	0.227656175	1
s__Salinicoccus_unclassified	0.863165918	0.73032669	1	0.863165918
s__Scardovia_unclassified	0.460734016	0.022702312	3.73E-06	0.857749793
s__Shewanella_unclassified	1	0.863165918	1	1
s__Shigella_flexneri	1	1	1	1
s__Shigella_sonnei	0.73032669	0.388837498	0.388837498	0.490582916
s__Sphaerochaeta_unclassified	3.38E-06	4.24E-08	6.13E-12	0.076442425
s__Sphingobacterium_unclassified	2.53E-05	3.87E-15	6.19E-09	0.052725374
s__Sphingobium_unclassified	1	1	1	1
s__Spiribacter_unclassified	0.400322151	7.43E-07	2.09E-07	0.636765011
s__Spiroplasma_unclassified	0.005824023	2.52E-07	6.19E-05	0.039941024
s__Sporolactobacillus_unclassified	0.227656175	0.00075817	0.562534608	0.084806282
s__Staphylococcus_lentus	0.009732748	0.694358086	0.73032669	0.863165918
s__Staphylococcus_xylosus	0.605130686	0.001152282	0.544079129	0.629402937
s__Stenotrophomonas_maltophilia	1	1	1	1
s__Stenotrophomonas_unclassified	1	1	1	1
s__Streptococcus_mitis_oralis_pneumoni	0.863165918	1	0.863165918	1
s__Subdoligranulum_unclassified	0.012095342	0.440050567	1.69E-05	0.961511632
s__Sulfurimonas_unclassified	1	0.73032669	1	1
s__Tannerella_forsythia	1	1	0.863165918	1
s__Terriglobus_unclassified	0.115999045	6.57E-05	9.85E-07	0.117607036
s__Thermanaerovibrio_unclassified	0.227656175	0.082954339	0.000124846	0.530437956
s__Thermoanaerobacter_ethanolicus	0.863165918	0.293117369	0.73032669	0.301102046
s__Thermodesulfatator_unclassified	0.863165918	0.863165918	1	0.73032669
s__Thermodesulfobacterium_unclassified	0.448259326	4.29E-05	0.001380332	0.230324003
s__Thermosipho_unclassified	0.167967537	3.31E-07	7.43E-07	0.868588482
s__Thermus_scotoductus	1	1	0.863165918	1
s__Thermus_unclassified	0.0386263	2.17E-05	3.92E-08	0.868588482
s__Thiomonas_unclassified	0.863165918	1	1	1
s__Thiothrix_unclassified	1	0.73032669	1	0.863165918
s__Treponema_denticola	1	1	0.863165918	1
s__Treponema_lecithinolyticum	1	1	0.863165918	1
s__Treponema_maltophilum	1	1	0.863165918	1
s__Treponema_medium	1	1	0.863165918	1
s__Treponema_primitia	0.863165918	0.863165918	1	1
s__Treponema_socranskii	1	1	0.863165918	1
s__Treponema_vincentii	1	1	0.863165918	1
s__Ureaplasma_parvum	1	1	0.73032669	1
s__Ureaplasma_unclassified	1	0.166906959	0.73032669	0.863165918
s__Veillonella_atypica	0.863165918	0.73032669	0.227656175	0.388837498
s__Veillonella_dispar	0.863165918	1	1	1
s__Veillonella_parvula	0.863165918	0.863165918	0.73032669	0.553269154
s__Veillonella_unclassified	0.000283865	0.023748551	3.38E-06	3.09E-11
s__Weissella_unclassified	0.41200831	0.06221249	4.55E-06	0.793348306
s__Wolbachia_pipientis	1	1	0.863165918	0.312521827
s__Wolbachia_unclassified	1	1	1	0.863165918
s__Yersinia_unclassified	1	0.73032669	0.863165918	0.73032669
s__zeta_proteobacterium_SCGC_AB_602	0.863165918	1	1	1

15 Control mice VS 50 OVX mice

Species	p-value(T1)	p-value(T2)	p-value(T3)	p-value(T4)
s__Acetobacter_unclassified	1	1	1	0.907038481
s__Acinetobacter_pittii_calcoaceticus_no	0.907038481	1	1	1
s__Acinetobacter_unclassified	1	0.907038481	0.907038481	0.726095162
s__Actinobacillus_unclassified	0.755496875	0.679896447	0.618315368	0.48352206
s__Actinoplanes_unclassified	1	1	0.907038481	1
s__Adlercreutzia_equolifaciens	0.533416513	0.554078824	0.564554878	0.791248558
s__Aerococcus_viridans	0.907038481	0.888557428	0.907038481	0.296860959
s__Aggregatibacter_segnis	0.907038481	1	1	1
s__Aggregatibacter_unclassified	0.815334594	0.350201389	0.907038481	0.888557428
s__Agrobacterium_tumefaciens	1	1	1	1
s__Agrobacterium_unclassified	1	1	1	1
s__Akkermansia_muciniphila	4.37E-05	0.99378854	0.175548807	0.607385323
s__Alicyclobacillus_unclassified	0.227557961	0.01992741	0.002275374	0.493294026
s__Alistipes_indistinctus	1.79E-05	0.888557428	0.374815795	0.233613001
s__Alistipes_onderdonkii	1	0.907038481	1	1
s__Alistipes_putredinis	0.165829938	7.66E-05	0.005580617	0.569828408
s__Alistipes_senegalensis	0.002050245	0.119470987	0.679896447	0.322813046
s__Alistipes_shahii	1	1	0.69709161	0.907038481
s__Alistipes_unclassified	0.18568637	0.130969965	0.134988062	0.720261359
s__Alloprevotella_rava	0.69709161	1	0.815334594	1
s__Alloprevotella_tannerae	0.69709161	1	0.815334594	1
s__Alloprevotella_unclassified	0.004171724	0.000770681	5.16E-05	0.37064075
s__alpha_proteobacterium_SCGC_AAA02	0.907038481	1	1	1
s__Anaerococcus_prevotii	0.640428787	0.086768173	0.063907089	0.640428787
s__Anaerococcus_vaginalis	0.907038481	0.436274929	1	0.640428787
s__Anaerostipes_unclassified	0.815334594	0.498219385	0.436274929	0.726095162
s__Anaerotruncus_colihominis	0.366494702	0.668524139	0.57512532	0.279202083
s__Anaerotruncus_sp_G3_2012	0.374815795	0.731944896	0.059569379	0.839596426
s__Anaerotruncus_unclassified	0.293270727	0.876269664	0.99378854	0.473856115
s__Arcobacter_unclassified	0.640428787	0.503170783	0.089672521	0.913210539
s__Asticcacaulis_unclassified	1	1	0.907038481	1
s__Bacillus_cereus_thuringiensis	1	1	0.436274929	0.907038481
s__Bacteriovorax_unclassified	0.190914616	0.00113742	0.001945517	0.937947457
s__Bacteroidales_bacterium_ph8	0.006435091	0.013300258	0.114026081	0.618315368
s__Bacteroides_caccae	0.180564753	0.275757848	0.102080961	0.391804855
s__Bacteroides_cellulosilyticus	0.198958931	0.143309283	0.445505761	0.513151034
s__Bacteroides_coprocola	0.640428787	0.019517481	0.130969965	0.779278328
s__Bacteroides_coprophilus	1	0.69709161	1	1
s__Bacteroides_dorei	0.006435091	0.095715856	0.564554878	0.152018494
s__Bacteroides_eggerthii	1	0.69709161	1	1
s__Bacteroides_finegoldii	0.128996012	0.242908261	0.156521501	0.944142
s__Bacteroides_fluxus	0.815334594	0.791248558	0.69709161	0.876269664
s__Bacteroides_fragilis	1	0.69709161	1	1
s__Bacteroides_helcogenes	1	0.907038481	1	0.907038481
s__Bacteroides_intestinalis	0.559304947	0.48352206	0.651608049	0.559304947
s__Bacteroides_massiliensis	0.00279493	0.001339333	0.002655757	0.043767333
s__Bacteroides_oleiciplenus	0.139100765	0.193569015	0.755496875	0.464297359
s__Bacteroides_ovatus	0.063907089	0.230571353	0.714443798	0.161124949
s__Bacteroides_pectinophilus	1	0.815334594	1	1
s__Bacteroides_plebeius	0.907038481	0.436274929	1	1
s__Bacteroides_pyogenes	1	0.69709161	1	1
s__Bacteroides_salanitronis	1	0.907038481	1	1
s__Bacteroides_salyersiae	1	1	0.907038481	1
s__Bacteroides_stercoris	0.559304947	0.596542367	0.907038481	0.815334594
s__Bacteroides_thetaiotaomicron	0.128996012	0.024956399	0.038376818	0.400471832
s__Bacteroides_uniformis	0.473856115	0.001573874	0.007757032	0.319017636
s__Bacteroides_vulgatus	0.48352206	0.089672521	0.803268221	0.039856098
s__Bacteroides_xylanisolvens	0.513151034	0.047997866	0.493294026	0.815334594
s__Bacteroidetes_bacterium_oral_taxon_	1	1	0.907038481	1
s__Bacteroidetes_oral_taxon_274	1	1	0.907038481	1
s__Bartonella_unclassified	0.06063023	0.006745048	0.00850523	0.773312592
s__Bifidobacterium_animalis	1	1	1	1
s__Bifidobacterium_dentium	1	1	1	1
s__Bifidobacterium_pseudolongum	0.180564753	0.326637782	0.10877847	0.319017636
s__Bilophila_wadsworthia	1	1	0.907038481	1
s__Blattabacterium_unclassified	0.198958931	0.00011276	0.00460073	0.596542367
s__Blautia_hydrogenotrophica	0.48352206	0.282675457	0.304129268	0.629330533
s__Blautia_producta	0.293270727	0.227557961	0.009317312	0.413686227
s__Borrelia_bavariensis	0.907038481	0.564554878	0.69709161	1
s__Borrelia_duttonii	1	1	0.907038481	1
s__Borrelia_recurrentis	0.69709161	1	0.69709161	0.726095162
s__Brachyspira_unclassified	0.128996012	0.000728333	0.000563187	0.803268221
s__Brevibacterium_unclassified	0.925570003	0.657227392	0.755496875	0.436274929
s__Brevundimonas_unclassified	1	1	1	0.69709161
s__Brucella_microti	0.907038481	0.779278328	0.815334594	0.585788415
s__Brucella_pinnipedialis	0.907038481	0.69709161	0.907038481	1
s__Buchnera_aphidicola	0.907038481	0.907038481	0.876269664	0.48352206
s__Bulleidia_extructa	1	1	0.907038481	1
s__Burkholderia_unclassified	1	0.907038481	1	1
s__Burkholderiales_bacterium_1_1_47	7.66E-05	0.06617189	0.358289787	0.268956636
s__Butyricicoccus_pullicaecorum	0.413686227	0.139100765	0.095715856	0.755496875
s__Butyricimonas_synergistica	0.00460073	0.523233418	0.518179547	0.651608049
s__Butyrivibrio_crossotus	1	0.436274929	1	1
s__Butyrivibrio_fibrisolvens	1	0.69709161	1	0.907038481
s__Butyrivibrio_unclassified	0.002655757	0.005580617	0.000154635	0.851786282
s__Campylobacter_rectus	1	1	0.907038481	1
s__Campylobacter_showae	1	1	0.907038481	1
s__Candidatus_Arthromitus_sp_SFB_1	0.907038481	0.864011651	1	0.779278328
s__Candidatus_Arthromitus_sp_SFB_5	1	1	1	0.907038481
s__Candidatus_Arthromitus_unclassified	0.063907089	0.585788415	0.268956636	0.255702002
s__Candidatus_Liberibacter_unclassified	0.559304947	0.210066246	0.418147743	0.845686737
s__Candidatus_Moranella_endobia	1	1	1	0.907038481
s__Candidatus_Pelagibacter_ubique	0.907038481	0.564554878	0.498219385	0.726095162
s__Candidatus_Phytoplasma_unclassified	0.07983872	0.105387504	0.629330533	0.518179547
s__Candidatus_Portiera_aleyrodidarum	1	0.69709161	1	1
s__Candidatus_Sulcia_muelleri	0.640428787	0.10877847	0.358289787	0.48352206
s__Candidatus_Uzinura_diaspidicola	0.907038481	0.888557428	0.815334594	0.907038481
s__Candidatus_Zinderia_insecticola	0.165829938	8.44E-05	0.006745048	0.255702002
s__Capnocytophaga_gingivalis	0.907038481	1	1	1
s__Capnocytophaga_unclassified	0.019114938	3.76E-06	1.85E-05	0.028700062
s__Caulobacter_unclassified	0.198958931	0.095715856	0.001491791	0.708642784
s__Cellulophaga_unclassified	0.042960146	0.000238302	0.004070305	0.218686496
s__Cellvibrio_unclassified	0.815334594	0.815334594	0.498219385	0.962744411
s__Chlamydophila_unclassified	0.691342048	0.679896447	0.409253012	0.282675457
s__Chlorobium_phaeobacteroides	0.007757032	7.37E-07	1.05E-05	0.559304947
s__Clostridiales_bacterium_1_7_47FAA	1	0.779278328	0.69709161	0.907038481
s__Clostridium_asparagiforme	0.48352206	0.418147743	0.089672521	0.350201389
s__Clostridium_citroniae	1	0.69709161	1	1
s__Clostridium_clostridioforme	0.038376818	0.000408725	4.83E-05	0.330491836
s__Clostridium_hathewayi	0.004489887	0.000217416	1.92E-05	0.839596426
s__Clostridium_hylemonae	0.640428787	0.888557428	0.791248558	0.815334594
s__Clostridium_leptum	0.907038481	0.907038481	1	1
s__Clostridium_nexile	1	0.69709161	1	1
s__Clostridium_pasteurianum	0.907038481	0.427155308	0.170637542	0.907038481
s__Clostridium_phytofermentans	1	0.907038481	1	1
s__Clostridium_ramosum	1	0.69709161	1	1
s__Clostridium_scindens	0.017209232	0.003016211	0.000116408	0.931756667
s__Clostridium_sp_ASF356	0.057493851	0.387514378	0.319017636	0.210066246
s__Clostridium_sp_ASF502	0.212911903	0.987577456	0.156521501	0.418147743
s__Clostridium_symbiosum	0.350201389	0.008312366	0.027584036	0.503170783
s__Collinsella_unclassified	0.002160122	0.020344835	0.014195744	0.09727655
s__Comamonas_unclassified	0.913210539	0.968950227	0.073365927	0.350201389
s__Coprobacillus_unclassified	0.000253256	0.57512532	0.102080961	0.413686227
s__Coprococcus_catus	0.907038481	0.358289787	0.779278328	0.815334594
s__Corynebacterium_crenatum	1	0.907038481	1	1
s__Corynebacterium_mastitidis	1	1	1	1
s__Crenarchaeota_archaeon_SCGC_AAA	0.640428787	0.193569015	0.876269664	0.913210539
s__Crenarchaeota_archaeon_SCGC_AAA	1	0.69709161	0.508148055	1
s__Cyanobium_unclassified	1	0.907038481	1	1
s__Cytophaga_unclassified	0.161124949	0.000342157	0.085344742	0.68561023
s__Deinococcus_unclassified	0.017209232	8.18E-05	8.72E-05	0.591154148
s__Desulfobulbus_sp_oral_taxon_041	1	1	0.907038481	0.907038481
s__Desulfovibrio_desulfuricans	0.190914616	0.731944896	0.607385323	0.007405518
s__Desulfovibrio_termitidis	0.413686227	0.02396768	0.374815795	0.10877847
s__Dorea_formicigenerans	0.293270727	0.01992741	0.115818913	0.720261359
s__Dorea_longicatena	0.413686227	0.105387504	0.051625033	0.827444925
s__Dorea_sp_5_2	0.02396768	0.523233418	0.028137281	0.293270727
s__Dorea_unclassified	0.907038481	0.436274929	0.779278328	0.289709749
s__Dysgonomonas_unclassified	0.230571353	1.40E-05	0.000120167	0.79725238
s__Echinicola_unclassified	0.07983872	0.000132143	0.014505844	0.657227392
s__Eggerthella_unclassified	1	0.282675457	0.791248558	0.815334594
s__Ehrlichia_unclassified	0.198958931	0.063907089	0.141192973	0.48352206
s__Elizabethkingia_unclassified	1	1	1	1
s__Enhydrobacter_aerosaccus	1	1	1	1
s__Enterobacter_cloacae	1	1	1	1
s__Enterococcus_faecalis	0.708642784	0.330491836	0.289709749	0.37064075
s__Enterococcus_gallinarum	0.326637782	0.334375199	0.559304947	0.559304947
s__Enterococcus_gilvus	1	1	1	1
s__Enterorhabdus_caecimuris	0.634869403	0.69709161	0.445505761	0.296860959
s__Erysipelotrichaceae_bacterium_2_2_4	0.907038481	1	1	1
s__Erysipelotrichaceae_bacterium_21_3	0.07089998	0.030446927	0.005580617	0.876269664
s__Erysipelotrichaceae_bacterium_5_2_5	0.907038481	1	0.69709161	0.907038481
s__Erysipelotrichaceae_bacterium_6_1_4	0.032917581	0.035557906	0.000473245	0.894711557
s__Escherichia_albertii	1	1	1	0.907038481
s__Escherichia_coli	0.962744411	1.50E-06	4.93E-07	5.46E-06
s__Escherichia_unclassified	0.400471832	0.012184114	0.021202619	0.387514378
s__Eubacteriaceae_bacterium_CM2	0.907038481	0.888557428	0.436274929	0.815334594
s__Eubacterium_cellulosolvens	1	0.304129268	0.518179547	0.726095162
s__Eubacterium_eligens	0.726095162	0.962744411	0.779278328	0.907038481
s__Eubacterium_hallii	0.815334594	0.242908261	0.508148055	0.815334594
s__Eubacterium_plexicaudatum	0.218686496	0.454846895	0.021202619	0.629330533
s__Eubacterium_ramulus	1	0.282675457	1	1
s__Eubacterium_rectale	0.720261359	0.4501626	0.679896447	0.559304947
s__Eubacterium_saphenum	1	1	0.907038481	1
s__Eubacterium_sp_14_2	0.876269664	0.907038481	0.779278328	0.69709161
s__Eubacterium_ventriosum	0.907038481	0.296860959	0.779278328	0.815334594
s__Exiguobacterium_unclassified	0.640428787	0.513151034	0.227557961	0.413686227
s__Facklamia_unclassified	1	1	0.907038481	1
s__Faecalibacterium_prausnitzii	0.047125304	0.003016211	0.000285841	0.68561023
s__Fervidobacterium_unclassified	0.726095162	0.779278328	0.119470987	0.640428787
s__Filifactor_alocis	1	1	0.907038481	1
s__Firmicutes_bacterium_M10_2	0.815334594	0.803268221	0.201694666	0.011918386
s__Flavonifractor_plautii	0.640428787	0.350201389	0.779278328	0.640428787
s__Fretibacterium_fastidiosum	1	1	0.907038481	1
s__Fusobacterium_nucleatum	1	1	0.907038481	1
s__Fusobacterium_periodonticum	1	1	1	1
s__Gardnerella_vaginalis	0.640428787	0.293270727	0.726095162	0.815334594
s__Gemella_unclassified	0.907038481	0.464297359	1	0.815334594
s__Geobacillus_unclassified	0.350201389	0.001616457	0.095715856	0.554078824
s__Gillisia_unclassified	0.021202619	1.05E-05	0.00011276	0.69709161
s__Granulicatella_unclassified	0.413686227	0.674200989	0.607385323	0.513151034
s__Granulicella_unclassified	0.094175274	0.342229787	0.224572744	0.743690911
s__Haemophilus_haemolyticus	1	0.907038481	1	1
s__Haemophilus_influenzae	1	0.907038481	1	1
s__Haemophilus_parainfluenzae	0.876269664	0.779278328	0.726095162	0.585788415
s__Halomonas_unclassified	0.640428787	0.224572744	0.175548807	0.950339923
s__Helicobacter_canadensis	0.046266123	0.000352497	0.000217416	0.900872031
s__Helicobacter_cinaedi	0.015806775	1.73E-05	0.000120167	0.528312469
s__Helicobacter_hepaticus	0.755496875	0.06170669	0.350201389	0.055478808
s__Helicobacter_pullorum	0.559304947	0.334375199	0.282675457	0.726095162
s__Helicobacter_winghamensis	0.009530847	0.001018863	4.67E-05	0.427155308
s__Hippea_unclassified	0.128996012	0.000102452	0.188287106	0.981367125
s__Hymenobacter_unclassified	0.01992741	1.55E-07	2.29E-05	0.839596426
s__Johnsonella_ignava	1	0.69709161	1	0.907038481
s__Kingella_unclassified	0.413686227	0.962744411	0.743690911	0.674200989
s__Klebsiella_pneumoniae	1	1	1	1
s__Klebsiella_unclassified	0.815334594	0.815334594	0.559304947	0.413686227
s__Lachnobacterium_bovis	1	0.779278328	1	0.907038481
s__Lachnospiraceae_bacterium_1_1_57F	0.815334594	0.147614803	0.252460504	0.322813046
s__Lachnospiraceae_bacterium_1_4_56F	0.350201389	0.322813046	0.145449844	0.68561023
s__Lachnospiraceae_bacterium_2_1_46F	1	0.596542367	0.436274929	0.726095162
s__Lachnospiraceae_bacterium_2_1_58F	1	1	1	0.907038481
s__Lachnospiraceae_bacterium_3_1_46F	0.018719672	0.289709749	0.020344835	0.102080961
s__Lachnospiraceae_bacterium_3_1_57F	0.350201389	0.165829938	0.017209232	0.564554878
s__Lachnospiraceae_bacterium_4_1_37F	0.48352206	0.255702002	0.119470987	0.937947457
s__Lachnospiraceae_bacterium_5_1_57F	0.48352206	0.498219385	0.119470987	0.791248558
s__Lachnospiraceae_bacterium_5_1_63F	1	0.585788415	0.69709161	1
s__Lachnospiraceae_bacterium_6_1_63F	1	0.907038481	1	1
s__Lachnospiraceae_bacterium_7_1_58F	0.212911903	0.000120167	4.22E-05	0.469063268
s__Lachnospiraceae_bacterium_8_1_57F	0.152018494	0.508148055	0.311514804	0.827444925
s__Lachnospiraceae_bacterium_9_1_43B	0.907038481	0.289709749	0.334375199	0.962744411
s__Lactobacillus_amylovorus	1	0.779278328	1	1
s__Lactobacillus_animalis	0.950339923	0.564554878	0.224572744	0.48352206
s__Lactobacillus_casei_paracasei	1	1	1	1
s__Lactobacillus_crispatus	0.907038481	1	0.815334594	1
s__Lactobacillus_delbrueckii	1	1	0.907038481	1
s__Lactobacillus_gasseri	5.89E-05	0.000650045	0.025463868	0.000770681
s__Lactobacillus_hominis	4.37E-06	1.30E-05	8.18E-05	7.99E-07
s__Lactobacillus_iners	1	0.57512532	0.48352206	0.907038481
s__Lactobacillus_jensenii	0.907038481	0.815334594	0.791248558	1
s__Lactobacillus_johnsonii	0.014505844	0.009317312	1.40E-05	0.030446927
s__Lactobacillus_murinus	0.007405518	0.69709161	0.296860959	0.190914616
s__Lactobacillus_reuteri	0.10877847	0.851786282	0.053522962	0.041383481
s__Lactobacillus_rhamnosus	1	1	1	1
s__Lactobacillus_sp_7_1_47FAA	1	1	0.815334594	1
s__Lactobacillus_sp_ASF360	1	1	0.907038481	1
s__Lactobacillus_vaginalis	0.002396234	0.030446927	0.001945517	0.006745048
s__Lactococcus_lactis	0.69709161	1	1	1
s__Leifsonia_unclassified	1	1	1	0.907038481
s__Leptotrichia_unclassified	0.907038481	1	1	1
s__Leuconostoc_pseudomesenteroides	1	1	1	1
s__Leuconostoc_unclassified	0.950339923	0.141192973	0.156521501	0.731944896
s__Listeria_unclassified	0.907038481	0.864011651	1	0.640428787
s__Lysinibacillus_sphaericus	1	0.242908261	0.907038481	1
s__Marinobacter_unclassified	0.907038481	0.907038481	0.907038481	1
s__Marvinbryantia_formatexigens	0.224572744	0.006745048	0.00506931	0.464297359
s__Megamonas_unclassified	1	0.791248558	1	1
s__Megasphaera_unclassified	0.047125304	0.0270402	0.040613703	0.201694666
s__Meiothermus_unclassified	0.907038481	0.69709161	1	1
s__Methanocaldococcus_unclassified	0.69709161	1	1	1
s__Methanothermococcus_unclassified	0.907038481	0.508148055	1	1
s__Methylotenera_versatilis	0.907038481	0.69709161	0.907038481	1
s__Micrococcus_luteus	1	1	0.907038481	1
s__Microcoleus_unclassified	0.907038481	1	1	1
s__Mitsuokella_unclassified	0.000516394	1.05E-05	1.99E-05	0.473856115
s__Mobiluncus_unclassified	1	0.518179547	1	1
s__Mucispirillum_schaedleri	0.326637782	0.618315368	0.003336111	0.282675457
s__Mycoplasma_capricolum	1	1	0.907038481	0.907038481
s__Mycoplasma_hyorhinis	1	1	1	1
s__Myroides_unclassified	0.128996012	0.003971129	0.034215983	0.68561023
s__Neisseria_elongata	0.907038481	1	1	1
s__Neisseria_flavescens	0.791248558	1	0.907038481	0.815334594
s__Neisseria_macacae	0.907038481	1	1	1
s__Neisseria_meningitidis	0.907038481	0.436274929	1	0.815334594
s__Neisseria_sicca	0.791248558	1	1	1
s__Neisseria_subflava	0.69709161	1	1	0.907038481
s__Neisseria_unclassified	0.149804305	0.001376038	0.042165578	0.059569379
s__Niabella_unclassified	0.198958931	9.30E-05	0.09727655	0.779278328
s__Nitrosospira_unclassified	1	0.779278328	1	1
s__Ochrobactrum_sp_CDB2	1	1	1	1
s__Odoribacter_laneus	0.047125304	0.006745048	0.081187406	0.937947457
s__Odoribacter_unclassified	0.493294026	0.815334594	0.69709161	0.839596426
s__Olsenella_unclassified	3.45E-05	0.000269086	0.001339333	0.020344835
s__Oribacterium_sp_oral_taxon_078	0.815334594	0.907038481	1	0.815334594
s__Oscillibacter_sp_1_3	0.919387837	0.418147743	0.851786282	0.839596426
s__Oscillibacter_unclassified	0.289709749	0.242908261	0.358289787	0.803268221
s__Oscillibacter_valericigenes	0.161124949	0.057493851	0.001616457	0.851786282
s__Oxalobacter_formigenes	0.907038481	1	0.69709161	0.907038481
s__Pantoea_dispersa	1	0.907038481	1	1
s__Pantoea_unclassified	0.975157923	0.102080961	0.350201389	0.293270727
s__Parabacteroides_distasonis	4.52E-05	0.900872031	0.47867576	0.147614803
s__Parabacteroides_goldsteinii	0.000749229	0.857894706	0.319017636	0.132967263
s__Parabacteroides_johnsonii	0.016147623	0.212911903	0.00279493	0.968950227
s__Parabacteroides_merdae	0.020344835	0.063907089	0.049783754	0.962744411
s__Parabacteroides_unclassified	0.000210845	0.743690911	0.152018494	0.170637542
s__Paraprevotella_unclassified	0.128996012	0.668524139	0.409253012	0.041383481
s__Parasutterella_excrementihominis	4.08E-05	0.05647885	0.12704525	0.196250417
s__Pasteurella_pneumotropica	0.554078824	0.554078824	0.580445397	0.907038481
s__Pediococcus_pentosaceus	0.815334594	1	1	1
s__Pediococcus_unclassified	0.086768173	0.000963974	0.012731364	0.987577456
s__Pedobacter_unclassified	0.000120167	2.13E-05	0.00279493	0.050697392
s__Peptostreptococcaceae_noname_unc	0.003507385	0.006745048	0.001339333	0.791248558
s__Peptostreptococcus_stomatis	1	1	0.907038481	1
s__Peptostreptococcus_unclassified	0.326637782	0.0270402	0.002724532	0.708642784
s__Porphyromonas_asaccharolytica	1	1	0.907038481	1
s__Porphyromonas_endodontalis	1	1	0.815334594	1
s__Porphyromonas_gingivalis	1	1	0.815334594	1
s__Prevotella_amnii	0.907038481	1	1	0.907038481
s__Prevotella_bivia	1	0.726095162	1	1
s__Prevotella_copri	0.815334594	0.585788415	0.554078824	1
s__Prevotella_disiens	1	1	0.907038481	1
s__Prevotella_histicola	0.907038481	1	1	0.69709161
s__Prevotella_intermedia	0.69709161	1	0.815334594	1
s__Prevotella_melaninogenica	0.755496875	0.779278328	0.640428787	0.720261359
s__Prevotella_nanceiensis	0.791248558	0.69709161	0.907038481	0.864011651
s__Prevotella_pallens	1	1	1	1
s__Prevotella_pleuritidis	1	1	0.815334594	1
s__Prevotella_salivae	1	1	1	1
s__Prevotella_stercorea	1	1	0.907038481	1
s__Prevotella_timonensis	0.907038481	1	1	1
s__Prevotella_veroralis	0.907038481	1	1	1
s__Propionibacterium_acnes	1	1	0.907038481	1
s__Proteus_mirabilis	0.000145237	0.014195744	0.102080961	0.128996012
s__Proteus_penneri	1	1	1	0.815334594
s__Proteus_unclassified	0.815334594	0.907038481	0.815334594	1
s__Providencia_unclassified	0.907038481	1	0.907038481	0.815334594
s__Pseudoflavonifractor_capillosus	0.358289787	0.00506931	0.003507385	0.585788415
s__Pseudomonas_aeruginosa	1	1	1	0.275757848
s__Pseudomonas_stutzeri	0.907038481	1	1	1
s__Pseudomonas_unclassified	0.523233418	0.445505761	0.937947457	0.731944896
s__Pusillimonas_unclassified	0.413686227	0.204457728	0.400471832	0.726095162
s__Rhodopseudomonas_palustris	1	0.907038481	1	1
s__Rhodospirillum_unclassified	0.907038481	0.815334594	0.646008425	0.726095162
s__Rickettsia_japonica	1	0.907038481	0.907038481	1
s__Riemerella_unclassified	0.907038481	0.221615618	0.391804855	0.640428787
s__Roseburia_hominis	0.242908261	0.036944471	0.000408725	0.436274929
s__Roseburia_intestinalis	0.242908261	0.0270402	0.001845721	0.400471832
s__Roseburia_inulinivorans	0.815334594	0.779278328	1	0.815334594
s__Roseburia_unclassified	0.021643204	0.036944471	0.000245672	0.533416513
s__Rothia_mucilaginosa	1	1	0.907038481	1
s__Rothia_unclassified	1	1	1	1
s__Ruminococcaceae_bacterium_D16	0.020344835	0.003336111	0.000303569	0.737810251
s__Ruminococcus_albus	0.035557906	0.002050245	0.000332101	0.913210539
s__Ruminococcus_bromii	1	0.69709161	1	1
s__Ruminococcus_flavefaciens	0.000579643	1.75E-06	1.13E-05	0.987577456
s__Ruminococcus_gnavus	0.198958931	0.08821071	0.005715509	0.640428787
s__Ruminococcus_lactaris	0.815334594	0.69709161	0.69709161	0.726095162
s__Ruminococcus_obeum	0.48352206	0.06617189	0.05647885	0.618315368
s__Ruminococcus_torques	0.018719672	7.66E-05	6.72E-05	0.864011651
s__Saccharomonospora_unclassified	0.907038481	0.242908261	0.69709161	1
s__Salinicoccus_unclassified	0.907038481	0.815334594	1	0.907038481
s__Scardovia_unclassified	0.913210539	0.307807377	0.053522962	0.554078824
s__Shewanella_unclassified	1	0.907038481	1	1
s__Shigella_flexneri	1	1	1	1
s__Shigella_sonnei	0.876269664	0.559304947	0.559304947	0.640428787
s__Sphaerochaeta_unclassified	0.001018863	0.000253256	1.67E-05	1
s__Sphingobacterium_unclassified	0.001573874	2.35E-07	0.000253256	0.839596426
s__Sphingobium_unclassified	1	1	1	1
s__Spiribacter_unclassified	0.326637782	0.036944471	0.020344835	0.346200974
s__Spiroplasma_unclassified	0.06170669	0.009317312	0.139100765	0.900872031
s__Sporolactobacillus_unclassified	0.413686227	0.082554526	0.651608049	0.242908261
s__Staphylococcus_lentus	0.436274929	0.374815795	0.815334594	0.907038481
s__Staphylococcus_xylosus	0.726095162	0.431701163	0.894711557	0.827444925
s__Stenotrophomonas_maltophilia	1	1	1	1
s__Stenotrophomonas_unclassified	1	1	1	1
s__Streptococcus_mitis_oralis_pneumoni	0.907038481	1	0.907038481	1
s__Subdoligranulum_unclassified	0.081187406	0.454846895	0.057493851	0.493294026
s__Sulfurimonas_unclassified	1	0.69709161	1	1
s__Tannerella_forsythia	1	1	0.907038481	1
s__Terriglobus_unclassified	0.161124949	0.041383481	0.007405518	0.743690911
s__Thermanaerovibrio_unclassified	0.413686227	0.215785288	0.145449844	0.950339923
s__Thermoanaerobacter_ethanolicus	0.907038481	0.779278328	0.69709161	0.69709161
s__Thermodesulfatator_unclassified	0.907038481	0.907038481	1	0.815334594
s__Thermodesulfobacterium_unclassified	0.538545365	0.046266123	0.404848186	0.864011651
s__Thermosipho_unclassified	0.350201389	0.004714045	0.053522962	0.585788415
s__Thermus_scotoductus	1	1	0.907038481	1
s__Thermus_unclassified	0.161124949	0.019517481	0.006745048	0.48352206
s__Thiomonas_unclassified	0.907038481	1	1	1
s__Thiothrix_unclassified	1	0.69709161	1	0.907038481
s__Treponema_denticola	1	1	0.907038481	1
s__Treponema_lecithinolyticum	1	1	0.907038481	1
s__Treponema_maltophilum	1	1	0.907038481	1
s__Treponema_medium	1	1	0.907038481	1
s__Treponema_primitia	0.907038481	0.779278328	1	1
s__Treponema_socranskii	1	1	0.907038481	1
s__Treponema_vincentii	1	1	0.907038481	1
s__Ureaplasma_parvum	1	1	0.815334594	1
s__Ureaplasma_unclassified	1	0.779278328	0.815334594	0.907038481
s__Veillonella_atypica	0.907038481	0.815334594	0.413686227	0.559304947
s__Veillonella_dispar	0.907038481	1	1	1
s__Veillonella_parvula	0.907038481	0.907038481	0.815334594	0.937947457
s__Veillonella_unclassified	0.180564753	0.013891508	0.000459626	5.26E-06
s__Weissella_unclassified	0.383252667	0.275757848	0.004829881	0.326637782
s__Wolbachia_pipientis	1	1	0.907038481	0.791248558
s__Wolbachia_unclassified	1	1	1	0.907038481
s__Yersinia_unclassified	1	0.815334594	0.907038481	0.815334594
s__zeta_proteobacterium_SCGC_AB_602	0.907038481	1	1	1

Supplementary Table 3 | The summary of data size and remove host rates.

Sample_ID	filter_Total	filter_Q10	filter_Q20	filter_Q30	filter_max	filter_min	filter_avgLe	filter_avgSt	filter_avgOA	filter_avgOA	filter_Q10	filter_Q20	filter_Q30	filter_remain	filter_rate	rmhost_Tot	rmhost_Alig	rmhost_Tot	rmhost_Alig	rmhost_rea	rmhost_rea	rmhost_sing	rmhost_max	rmhost_min	rmhost_avg_length
T1_20800	299173888	0.9884	0.98	0.9283	100	30	95	4	0.9507	14	0.9884	0.98	0.9283	297223699	0.9935	147673674	142003297	1876351	1838244	1.335E+10	1.357E+10	140618393	100	30	94.66731423
T1_20801	277791940	0.9884	0.9803	0.9303	100	30	95	4	0.9514	14	0.9884	0.9803	0.9303	276022757	0.9936	137160441	132891651	1701875	1673528	1.253E+10	1.271E+10	128315415	100	30	94.85399597
T1_20802	201095004	0.9883	0.98	0.9287	100	30	95	4	0.9506	14	0.9883	0.98	0.9287	199770722	0.9934	99251240	95989120	1268242	1247379	9.04E+09	9.139E+09	92896188	100	30	94.55897117
T1_20803	170778734	0.9882	0.9792	0.9253	100	30	94	4	0.9487	14	0.9882	0.9792	0.9253	169529936	0.9927	84166164	79617612	1197608	1159184	7.451E+09	7.564E+09	86510623	100	30	94.15075612
T1_20804	174735588	0.9882	0.9793	0.9249	100	30	94	4	0.9488	14	0.9882	0.9793	0.9249	173429433	0.9925	86087823	65962202	1253787	1020627	6.144E+09	6.262E+09	76745830	100	30	93.8977931
T1_20806	191630682	0.9881	0.9787	0.9227	100	30	94	4	0.9474	14	0.9881	0.9787	0.9227	190127998	0.9922	94341989	91660926	1444020	1426344	8.52E+09	8.717E+09	107435408	100	30	93.88070876
T1_20808	245166402	0.9884	0.98	0.929	100	30	95	4	0.9508	14	0.9884	0.98	0.929	243581585	0.9935	121031249	110987177	1519087	1438449	1.046E+10	1.056E+10	107450653	100	30	94.58405344
T1_20809	226952246	0.9883	0.98	0.929	100	30	95	4	0.9506	14	0.9883	0.98	0.929	225441427	0.9933	111995048	101642082	1451331	1365434	9.562E+09	9.69E+09	103557807	100	30	94.57912572
T1_20810	357508492	0.9878	0.9773	0.9187	100	30	93	5	0.9443	13	0.9878	0.9773	0.9187	354648144	0.992	175975691	172155583	2696762	2669496	1.627E+10	1.578E+10	188601060	100	30	92.91609501
T1_20811	216398388	0.9876	0.976	0.913	100	30	92	5	0.9412	13	0.9876	0.976	0.913	214391957	0.9907	106252808	102258762	1886341	1849406	9.55E+09	9.316E+09	128881897	100	30	92.04771278
T1_20812	198730794	0.9875	0.9757	0.9117	100	30	92	6	0.9405	13	0.9875	0.9757	0.9117	196841395	0.9905	97530449	72804176	1780497	1380890	6.784E+09	6.595E+09	96506827	100	30	91.67404817
T1_20813	303589954	0.9878	0.9774	0.9188	100	30	93	5	0.9443	13	0.9878	0.9774	0.9188	301160662	0.992	149432453	117042705	2295756	1935704	1.099E+10	1.07E+10	136027800	100	30	92.47498513
T1_20814	213874388	0.9876	0.9763	0.9139	100	30	92	5	0.9417	13	0.9876	0.9763	0.9139	211871426	0.9906	104994404	85942823	1882618	1640318	8.008E+09	7.795E+09	113341082	100	30	91.72256252
T1_20815	215031432	0.9877	0.9769	0.9172	100	30	93	5	0.9432	13	0.9877	0.9769	0.9172	213218796	0.9916	105756494	91564414	1705808	1558851	8.575E+09	8.39E+09	108500816	100	30	92.44463657
T1_20816	263691752	0.9875	0.9758	0.9118	100	30	92	5	0.9406	13	0.9875	0.9758	0.9118	261226236	0.9907	129449969	127069047	2326298	2304306	1.184E+10	1.161E+10	162866663	100	30	92.06933721
T1_20817	292317512	0.9878	0.9774	0.9194	100	30	93	5	0.9444	13	0.9878	0.9774	0.9194	289981322	0.992	143889633	131694394	2202056	2097949	1.24E+10	1.212E+10	147424923	100	30	92.87818476
T1_20818	286221766	0.9863	0.9728	0.9151	100	30	93	4	0.9316	13	0.9863	0.9728	0.9151	283244042	0.9896	140218166	138884810	2807710	2792051	1.27E+10	1.308E+10	208557583	100	30	92.62449177
T1_20819	224333462	0.9858	0.9701	0.903	100	30	91	5	0.9249	12	0.9858	0.9701	0.903	221442004	0.9871	109366268	107923451	2709468	2690752	9.751E+09	9.957E+09	194997652	100	30	91.0738837
T1_20820	175833660	0.9856	0.9692	0.899	100	30	90	5	0.923	12	0.9856	0.9692	0.899	173268504	0.9854	85440099	84332557	2388306	2372769	7.553E+09	7.722E+09	168481215	100	30	90.29202679
T1_20832	151112526	0.9856	0.9694	0.9001	100	30	90	5	0.9235	12	0.9856	0.9694	0.9001	148932739	0.9856	73450508	66173978	2031723	1889836	5.911E+09	6.062E+09	134122273	100	30	90.19103967
T1_20842	200928376	0.9858	0.97	0.9025	100	30	91	5	0.9249	12	0.9858	0.97	0.9025	198184906	0.9863	97812100	89910138	2560706	2399146	8.041E+09	8.305E+09	171119954	100	30	90.64776502
T1_20843	226202176	0.986	0.9712	0.9084	100	30	92	5	0.9277	12	0.986	0.9712	0.9084	223514462	0.9881	110493926	107456078	2526610	2469139	9.746E+09	1.001E+10	181202227	100	30	91.71717051
T1_20845	248080640	0.986	0.9713	0.9085	100	30	92	5	0.928	12	0.986	0.9713	0.9085	245070110	0.9879	121123324	118776402	2823462	2778784	1.075E+10	1.108E+10	202898377	100	30	91.6497071
T1_20846	227085280	0.9861	0.9717	0.9097	100	30	92	4	0.9288	12	0.9861	0.9717	0.9097	224438359	0.9883	110974824	110128447	2488711	2477357	9.992E+09	1.029E+10	182973783	100	30	91.89906248
T1_20847	308275750	0.9876	0.9761	0.9131	100	30	92	5	0.9412	13	0.9876	0.9761	0.9131	305538161	0.9911	151480356	147140833	2577449	2536507	1.378E+10	1.342E+10	177308995	100	30	92.23550776
T1_20849	216136256	0.9877	0.9771	0.9176	100	30	93	5	0.9436	13	0.9877	0.9771	0.9176	214355402	0.9918	106338398	75051762	1678606	1283170	7.058E+09	6.819E+09	89890686	100	30	92.25965484
T1_20850	180047798	0.9875	0.9757	0.9114	100	30	92	6	0.9404	13	0.9875	0.9757	0.9114	178322547	0.9904	88349728	75950226	1623091	1435469	7.053E+09	6.907E+09	99190911	100	30	91.68539582
T1_20851	180439764	0.9874	0.9753	0.9091	100	30	92	6	0.9394	13	0.9874	0.9753	0.9091	178665384	0.9902	88498854	81023790	1667676	1569968	7.52E+09	7.33E+09	107792545	100	30	91.42091156
T1_20853	278444350	0.9876	0.976	0.9129	100	30	92	5	0.9412	13	0.9876	0.976	0.9129	275790949	0.9905	136648860	112741031	2493229	2181119	1.048E+10	1.024E+10	150868557	100	30	91.64936415
T1_20854	261635786	0.9878	0.9772	0.9187	100	30	93	5	0.9441	13	0.9878	0.9772	0.9187	259413709	0.9915	128662320	113960926	2089069	1895220	1.078E+10	1.036E+10	132773871	100	30	92.55945942
T1_20855	243906496	0.9877	0.9765	0.9153	100	30	92	5	0.9424	13	0.9877	0.9765	0.9153	241718036	0.991	119829871	111543733	2058294	1971200	1.045E+10	1.017E+10	137223380	100	30	92.21170445
T1_20857	249839046	0.9875	0.9756	0.9109	100	30	92	6	0.9401	13	0.9875	0.9756	0.9109	247431757	0.9904	122586244	112599946	2259269	2127006	1.051E+10	1.019E+10	146226872	100	30	91.67946742
T1_20859	252356090	0.9885	0.9807	0.9306	100	30	95	3	0.9524	14	0.9885	0.9807	0.9306	250986085	0.9946	124830615	122653043	1324855	1310696	1.158E+10	1.177E+10	102193251	100	30	95.12095786
T1_20860	376856230	0.9885	0.9803	0.9282	100	30	95	4	0.9514	14	0.9885	0.9803	0.9282	374687960	0.9942	186296909	185032675	2094142	2086842	1.743E+10	1.769E+10	160684027	100	30	94.80072674
T1_20861	206468310	0.9882	0.9786	0.9196	100	30	94	5	0.947	13	0.9882	0.9786	0.9196	204964795	0.9927	101759359	98929273	1446077	1422106	9.185E+09	9.356E+09	105593904	100	30	93.56770515
T1_20862	212135068	0.988	0.9774	0.9135	100	30	93	5	0.944	13	0.988	0.9774	0.9135	210374480	0.9917	104339873	103559181	1694734	1690430	9.537E+09	9.748E+09	125176988	100	30	92.95660298
T1_20863	259837450	0.9882	0.9789	0.9208	100	30	94	5	0.9477	14	0.9882	0.9789	0.9208	257968662	0.9928	128084674	103093332	1799314	1525742	9.56E+09	9.771E+09	114719847	100	30	93.61967979
T1_20864	319799162	0.9884	0.9798	0.926	100	30	95	4	0.9502	14	0.9884	0.9798	0.926	317812553	0.9938	157946851	150913280	1918851	1860159	1.414E+10	1.442E+10	143155669	100	30	94.51605788
T1_20865	224943326	0.9883	0.9796	0.925	100	30	94	4	0.9497	14	0.9883	0.9796	0.925	223471892	0.9935	111027118	104480186	1417656	1369281	9.772E+09	9.926E+09	103033667	100	30	94.14646987
T1_20866	352866070	0.9885	0.9804	0.9285	100	30	95	4	0.9517	14	0.9885	0.9804	0.9285	350785319	0.9941	174388425	168637424	2008469	1975030	1.585E+10	1.609E+10	150680348	100	30	94.5924321
T1_20867	284844542	0.9867	0.9721	0.9017	100	30	89	8	0.9334	13	0.9867	0.9721	0.9017	279974647	0.9829	137740925	135656763	4492797	4444448	1.219E+10	1.215E+10	301853199	100	30	89.36233157
T1_20868	168910832	0.9861	0.9691	0.8895	100	30	87	9	0.9271	12	0.9861	0.9691	0.8895	165189433	0.978	80896069	78298286	3397295	3333764	6.86E+09	6.822E+09	217878061	100	30	86.90972696
T1_20869	142684896	0.9863	0.9703	0.894	100	30	87	9	0.9298	12	0.9863	0.9703	0.894	139747617	0.9794	68539709	61758111	2668199	2497007	5.476E+09	5.339E+09	160403407	100	30	87.10033023
T1_20870	158266802	0.9866	0.9716	0.8996	100	30	89	8	0.9322	12	0.9866	0.9716	0.8996	155441167	0.9821	76421475	74168676	2598217	2550092	6.659E+09	6.565E+09	170959083	100	30	88.77417286
T1_20871	189679492	0.9864	0.9708	0.8975	100	30	88	8	0.9308	12	0.9864	0.9708	0.8975	186072096	0.981	91393344	90117538	3285408	3262877	8.121E+09	7.849E+09	213028379	100	30	88.19213337
T1_20872	229300152	0.9866	0.9719	0.9002	100	30	89	8	0.9329	12	0.9866	0.9719	0.9002	225226037	0.9822	110739038	110173467	3747961	3737165	9.877E+09	9.798E+09	251062909	100	30	88.92352608
T1_20873	225320764	0.9864	0.9706	0.8958	100	30	88	8	0.9302	12	0.9864	0.9706	0.8958	220994107	0.9808	108515917	103746851	3962273	3851855	9.224E+09	9.14E+09	254060350	100	30	88.09353726
T1_20874	224516522	0.9864	0.9707	0.8965	100	30	88	8	0.9304	12	0.9864	0.9707	0.8965	220228028	0.9809	108145573	107791506	3936882	3930298	9.599E+09	9.55E+09	261748025	100	30	88.42596146
T1_20875	203385776	0.9775	0.977	0.9684	63	30	45	31	0.9907	20	0.9775	0.977	0.9684	168723331	0.8296	76659963	74426748	15403405	15148254	3.363E+09	3.348E+09	610889448	63	30	44.64002259
T1_20876	278768984	0.9773	0.9767	0.9667	63	30	44	32	0.9907	20	0.9773	0.9767	0.9667	219495362	0.7874	96966083	94917269	25563196	25152780	4.259E+09	4.24E+09	1.004E+09	63	30	44.20339098
T1_20877	236088742	0.9772	0.9766	0.9664	63	30	44	32	0.9907	20	0.9772	0.9766	0.9664	180945473	0.7664	79348652	64672935	22248169	19055599	2.889E+09	2.882E+09	755622752	63	30	43.97732942
T1_20878	214105552	0.9772	0.9766	0.9663	63	30	44	32	0.9907	20	0.9772	0.9766	0.9663	164670125	0.7691	71915080	70957840	20839965	20685202	3.176E+09	3.163E+09	824861863	63	30	44.05957364
T1_20879	262322688	0.9772	0.9767	0.9667	63	30	44	32	0.9907	20	0.9772	0.9767	0.9667	204589315	0.7799	90370476	86845034	23848363	23178068	3.887E+09	3.877E+09	920399445	63	30	44.11059398
T1_20880	240328148	0.977	0.9764	0.9656	63	30	44	32	0.9907	20	0.977	0.9764	0.9656	178387839	0.7423	77056297	58172938	24275245	19274539	2.588E+09	2.579E+09	761767459	63	30	43.72091382
T1_20881	260928536	0.9772	0.9765	0.9661	62	30	44	32	0.9907	20	0.9772	0.9765	0.9661	199054457	0.7629	86760425	82666735	25533607	24734416	3.696E+09	3.678E+09	983231277	62	30	43.96556764
T1_20882	256339304	0.9772	0.9766	0.9663	62	30	44	32	0.9907	20	0.9772	0.9766	0.9663	198065004	0.7727	87504897	86564487	23055210	22938373	3.863E+09	3.867E+09	905693472	62	30	44.04510384
T1_20884	235660708	0.9771	0.9765	0.9664	62	30	44	33	0.9907	20	0.9771	0.9765	0.9664	182018159	0.7724	79935672	55299947	22146815	16831193	2.449E+09	2.463E+09	668638475	62	30	43.78703815
T1_20885	246875044	0.9772	0.9766	0.9665	62	30	44	32	0.9907	20	0.9772	0.9766	0.9665	193494710	0.7838	85102349	74619492	23290012	21167487	3.315E+09	3.332E+09	847030839	62	30	43.9759653
T1_20887	231378816	0.9772	0.9766	0.9666	62	30	44	33	0.9907	20	0.9772	0.9766	0.9666	180895882	0.7818	79983577	77693101	20928728	20524761	3.442E+09	3.473E+09	816425991	62	30	43.95215978
T1_20888	187622550	0.9772	0.9766	0.9668	63	30	44	32	0.9907	20	0.9772	0.9766	0.9668	147785338	0.7877	65439824	64867802	16905690	16830003	2.873E+09	2.904E+09	672962925	63	30	44.00239397
T1_20895	206502012	0.9772	0.9766	0.9669	63	30	44	33	0.9907	20	0.9772	0.9766	0.9669	164393023	0.7961	73217422	72650653	17958179	17874486	3.216E+09	3.252E+09	714251264	63	30	44.01873716
T1_20896	128218036	0.9769	0.9763	0.9659	63	30	44	33	0.9907	20	0.9769	0.9763	0.9659	94112870	0.734	40732502	30318778	12647866	10309661	1.334E+09	1.346E+09	404037505	63	30	43.4607233
T1_20897	221431356	0.9772	0.9766	0.9667	63	30	44	33	0.9907	20	0.9772	0.9766	0.9667	173414029	0.7832	76723330	75423275	19967369	19766500	3.344E+09	3.37E+09	785485070	63	30	43.95576288
T1_20898	236025082	0.9771	0.9764	0.966	63	30	44	33	0.9907	20	0.9771	0.9764	0.966	180954961	0.7667	79189730	77279550	22575501	22279538	3.419E+09	3.445E+09	883718823	63	30	43.81373345
T1_20899	254221314	0.9771	0.9764	0.9655	63	30	44	32	0.9907	20	0.9771	0.9764	0.9655	193075391	0.7595	83747020	82904068	25581351	25436376	3.664E+09	3.697E+09	1.019E+09	63	30	43.82139181
T2_20800	240469020	0.9771	0.9765	0.9662	63	30	44	32	0.9907	20	0.9771	0.9765	0.9662	184873090	0.7688	81252075	80676703	22368940	22265364	3.556E+09	3.608E+09	890760973	63	30	43.86521805
T2_20801	215828436	0.9769	0.9762	0.965	62	30	44	33	0.9907	20	0.9769	0.9762	0.965	156992481	0.7274	67859479	67724504	21273523	21253116	2.968E+09	3.015E+09	838061168	62	30	43.53321917
T2_20802	170316716	0.9769	0.9762	0.9649	62	30	43	33	0.9907	20	0.9769	0.9762	0.9649	122729961	0.7206	53034294	50709136	16661373	16150845	2.219E+09	2.257E+09	634829532	62	30	43.46620145
T2_20803	236898298	0.977	0.9763	0.9652	62	30	44	33	0.9907	20	0.977	0.9763	0.9652	175395077	0.7404	76366101	70731971	22662875	21245916	3.097E+09	3.157E+09	839554980	62	30	43.59904799
T2_20804	222239656	0.9769	0.9763	0.9653	62	30	44	33	0.9907	20	0.9769	0.9763	0.9653	162773279	0.7324	70550869	59844954	21671541	19201949	2.618E+09	2.665E+09	757920138	62	30	43.49255657
T2_20806	219412192	0.977	0.9763	0.9656	62	30	44	33	0.9907	20	0.977	0.9763	0.9656	161981231	0.7383	70238760	57057645	21503711	18254232	2.508E+09	2.54E+09	723697992	62	30	43.60674546
T2_20808	236800126	0.9769	0.9761	0.9646	63	30	43	32	0.9907	20	0.9769	0.9761	0.9646	169771086	0.7169	72617615	71902729	24535856	24416929	3.152E+09	3.194E+09	962952154	63	30	43.44834882
T2_20809	229983186	0.9771	0.9765	0.9661	63	30	44	32	0.9907	20	0.9771	0.9765	0.9661	176580194	0.7678	77894091	75498244	20792012	20430344	3.329E+09	3.384E+09	814002814	63	30	43.90887247
T2_20810	213802034	0.9769	0.9762	0.965	62	30	44	33	0.9907	20	0.9769	0.9762	0.965	157510322	0.7367	68140385	61021904	21229552	19616624	2.671E+09	2.715E+09	774556002	62	30	43.48618798
T2_20811	225566240	0.9771	0.9764	0.9657	62	30	44	32	0.9907	20	0.9771	0.9764	0.9657	168344668	0.7463	73149760	72940072	22045148	22011174	3.221E+09	3.254E+09	876036487	62	30	43.78368133
T2_20812	201306544	0.977	0.9763	0.9654	62	30	44	32	0.9907	20	0.977	0.9763	0.9654	149249337	0.7414	64122193	64012175	21004951	20984133	2.824E+09	2.846E+09	837369454	62	30	43.67231482
T2_20813	217829460	0.9771	0.9765	0.9664	62	30	44	33	0.9907	20	0.9771	0.9765	0.9664	169452658	0.7779	75027681	75006091	19397296	19393027	3.294E+09	3.359E+09	777146998	62	30	43.85863969
T2_20814	269771706	0.9772	0.9766	0.9664	63	30	44	33	0.9907	20	0.9772	0.9766	0.9664	212508368	0.7877	94680050	93829558	23148268	23003657	4.134E+09	4.205E+09	917068115	63	30	43.93696898
T2_20815	250819282	0.9769	0.9762	0.9652	62	30	44	32	0.9907	20	0.9769	0.9762	0.9652	181404592	0.7232	78142855	68836887	25118882	23066062	3.023E+09	3.056E+09	908475976	62	30	43.47495744
T2_20816	175770580	0.977	0.9763	0.9655	63	30	44	33	0.9907	20	0.977	0.9763	0.9655	130005145	0.7396	56437711	31166214	17129723	10248413	1.369E+09	1.384E+09	405460108	62	30	43.51733472
T2_20817	472042826	0.9766	0.9758	0.9642	62	30	43	32	0.9907	20	0.9766	0.9758	0.9642	334065586	0.7077	141934620	141793049	50196346	50160928	6.098E+09	6.262E+09	1.995E+09	62	30	43.01086543
T2_20818	410349248	0.9765	0.9756	0.9636	62	30	43	32	0.9907	20	0.9765	0.9756	0.9636	277476230	0.6762	115830136	110036511	45815958	44252818	4.699E+09	4.841E+09	1.756E+09	62	30	42.7375572
T2_20819	337450088	0.9764	0.9756	0.9633	62	30	43	32	0.9907	20	0.9764	0.9756	0.9633	223858232	0.6634	92741059	87472981	38376114	36516625	3.736E+09	3.839E+09	1.445E+09	62	30	42.65323623
T2_20820	278696626	0.9763	0.9754	0.9625	62	30	43	32	0.9907	20	0.9763	0.9754	0.9625	175573723	0.63	71576377	68893502	32420969	31603164	2.923E+09	3.033E+09	1.247E+09	62	30	42.52494101
T2_20832	251527588	0.9764	0.9756	0.9632	62	30	43	33	0.9907	20	0.9764	0.9756	0.9632	161920467	0.6437	66404947	57452069	29110573	26127063	2.432E+09	2.53E+09	1.034E+09	62	30	42.51438905
T2_20842	374644164	0.9764	0.9755	0.9629	62	30	43	32	0.9907	20	0.9764	0.9755	0.9629	244393758	0.6523	101093986	94587148	42205786	40434908	4.036E+09	4.159E+09	1.592E+09	62	30	42.6253004
T2_20843	363218350	0.9764	0.9756	0.9634	62	30	43	32	0.9907	20	0.9764	0.9756	0.9634	241737417	0.6655	100496866	97163220	40743685	39808622	4.145E+09	4.269E+09	1.578E+09	62	30	42.67860032
T2_20845	416162892	0.9766	0.9758	0.9644	62	30	43	31	0.9906	20	0.9766	0.9758	0.9644	297705736	0.7154	126427471	125864799	44850794	44687180	5.414E+09	5.537E+09	1.786E+09	62	30	42.97244129
T2_20846	320017876	0.9876	0.9763	0.9163	100	30	93	5	0.9421	13	0.9876	0.9763	0.9163	317230381	0.9913	157298068	153315640	2634245	2590487	1.43E+10	1.423E+10	185447547	100	30	92.89183103
T2_20847	208937980	0.9874	0.9754	0.912	100	30	92	5	0.9399	13	0.9874	0.9754	0.912	206905804	0.9903	102490440	101836385	1924924	1917672	9.408E+09	9.436E+09	138494890	100	30	92.32830853
T2_20849	261244792	0.9876	0.9763	0.9159	100	30	93	5	0.942	13	0.9876	0.9763	0.9159	259027798	0.9915	128463045	127610302	2101708	2093988	1.192E+10	1.184E+10	151503185	100	30	92.94526103
T2_20850	302403144	0.9878	0.9772	0.9197	100	30	93	4	0.9444	13	0.9878	0.9772	0.9197	300125441	0.9925	148984649	146210492	2156143	2135532	1.376E+10	1.36E+10	153296797	100	30	93.38205669
T2_20851	221141608	0.9874	0.9752	0.9099	100	30	92	5	0.9393	13	0.9874	0.9752	0.9099	218868783	0.9897	108362038	106939779	2144707	2122651	9.876E+09	9.82E+09	149885615	100	30	91.87856546
T2_20853	259008844	0.9876	0.9765	0.9161	100	30	93	5	0.9425	13	0.9876	0.9765	0.9161	256734427	0.9912	127290051	125867388	2154325	2140529	1.172E+10	1.169E+10	154462092	100	30	92.83023506
T2_20854	267099162	0.9875	0.9759	0.9137	100	30	93	5	0.9409	13	0.9875	0.9759	0.9137	264601022	0.9906	131116055	126936426	2368912	2323899	1.175E+10	1.179E+10	168346713	100	30	92.51432087
T2_20855	232302474	0.9875	0.9759	0.9134	100	30	92	5	0.9409	13	0.9875	0.9759	0.9134	230096949	0.9905	114006056	113281219	2084837	2078010	1.05E+10	1.048E+10	147927790	100	30	92.40638227
T2_20857	248262318	0.9869	0.9734	0.9072	100	30	90	7	0.9362	13	0.9869	0.9734	0.9072	244436098	0.9846	120459855	117179036	3516388	3446766	1.071E+10	1.048E+10	231298364	100	30	90.08451286
T2_20859	233481988	0.9865	0.9712	0.8981	100	30	89	8	0.9314	12	0.9865	0.9712	0.8981	229119838	0.9813	112553926	112314845	4011986	4006131	1.001E+10	1.002E+10	268579572	100	30	88.75991266
T2_20860	218790648	0.9867	0.9723	0.9022	100	30	89	8	0.9337	13	0.9867	0.9723	0.9022	214904671	0.9822	105654033	105512810	3596605	3593603	9.453E+09	9.481E+09	247989787	100	30	89.37399832
T2_20861	136497478	0.9864	0.9709	0.8966	100	30	88	9	0.9309	12	0.9864	0.9709	0.8966	133745542	0.9798	65616568	55924119	2512406	2215318	4.963E+09	4.902E+09	145433721	100	30	87.76674822
T2_20862	214433884	0.9867	0.9723	0.9024	100	30	89	8	0.9339	13	0.9867	0.9723	0.9024	210703780	0.9826	103639408	103284600	3424964	3418980	9.341E+09	9.163E+09	229540824	100	30	89.21123055
T2_20863	184957232	0.9865	0.9714	0.8988	100	30	89	8	0.9319	12	0.9865	0.9714	0.8988	181527711	0.9815	89183094	83323453	3161523	2978524	7.455E+09	7.388E+09	201994996	100	30	88.69177766
T2_20864	203751154	0.9867	0.9722	0.9023	100	30	89	8	0.9335	13	0.9867	0.9722	0.9023	200164847	0.9824	98443659	96992097	3277529	3251685	8.765E+09	8.596E+09	214621339	100	30	89.10797828
T2_20865	227561466	0.9868	0.973	0.9055	100	30	90	7	0.9353	13	0.9868	0.973	0.9055	223852172	0.9837	110224136	107400366	3403900	3349135	9.764E+09	9.574E+09	223768452	100	30	89.67281325
T2_20866	315354256	0.9883	0.9796	0.9249	100	30	95	4	0.9497	14	0.9883	0.9796	0.9249	313408745	0.9938	155765466	154968816	1877813	1873309	1.452E+10	1.48E+10	142623554	100	30	94.49394129
T2_20867	280137718	0.9886	0.981	0.9314	100	30	95	4	0.9532	14	0.9886	0.981	0.9314	278744268	0.995	138699262	138573102	1345744	1345111	1.317E+10	1.324E+10	102854920	100	30	95.18874866
T2_20868	298815566	0.9884	0.9801	0.9275	100	30	95	4	0.9509	14	0.9884	0.9801	0.9275	297055902	0.9941	147677893	144055952	1700116	1670712	1.353E+10	1.379E+10	128564974	100	30	94.70952586
T2_20869	224952316	0.9883	0.9794	0.9225	100	30	94	4	0.9488	14	0.9883	0.9794	0.9225	223501711	0.9936	111048780	109923739	1404151	1397096	1.024E+10	1.046E+10	107145406	100	30	94.05571837
T2_20870	291980894	0.9882	0.9785	0.9185	100	30	93	5	0.9466	13	0.9882	0.9785	0.9185	289732626	0.9923	143784730	118856989	2163166	1885610	1.096E+10	1.124E+10	140491740	100	30	93.22062091
T2_20871	318695080	0.9884	0.9798	0.9257	100	30	94	4	0.95	14	0.9884	0.9798	0.9257	316684435	0.9937	157375936	156570109	1932563	1927469	1.472E+10	1.485E+10	143108954	100	30	94.32614462
T2_20872	277644184	0.9882	0.9789	0.9212	100	30	94	5	0.9479	14	0.9882	0.9789	0.9212	275661790	0.9929	136877705	131947370	1906380	1868270	1.227E+10	1.25E+10	138862268	100	30	93.7497542
T2_20873	310302954	0.9884	0.98	0.9262	100	30	94	4	0.9505	14	0.9884	0.98	0.9262	308409852	0.9939	153293800	142519664	1822252	1731944	1.338E+10	1.357E+10	129627622	100	30	94.418459
T2_20874	185110916	0.9876	0.9764	0.9145	100	30	92	5	0.942	13	0.9876	0.9764	0.9145	183428523	0.9909	90923746	89180231	1581031	1559038	8.355E+09	8.139E+09	108534522	100	30	92.27836784
T2_20875	275351312	0.9876	0.9762	0.9139	100	30	92	5	0.9418	13	0.9876	0.9762	0.9139	272833498	0.9909	135234376	133778810	2364746	2351807	1.256E+10	1.215E+10	162556889	100	30	92.1422452
T2_20876	237105524	0.9877	0.9768	0.9168	100	30	93	5	0.943	13	0.9877	0.9768	0.9168	235103123	0.9916	116608752	116284141	1885619	1882483	1.094E+10	1.066E+10	132503825	100	30	92.70261027
T2_20877	194528546	0.9875	0.9754	0.9104	100	30	92	6	0.9398	13	0.9875	0.9754	0.9104	192596326	0.9901	95393780	91997650	1808766	1772297	8.583E+09	8.306E+09	120916489	100	30	91.56281445
T2_20878	271758482	0.9878	0.9773	0.9182	100	30	93	5	0.9441	13	0.9878	0.9773	0.9182	269483439	0.9916	133671265	133627119	2140909	2140223	1.261E+10	1.224E+10	150367281	100	30	92.8049315
T2_20879	240264786	0.9875	0.9758	0.9118	100	30	92	6	0.9406	13	0.9875	0.9758	0.9118	237962841	0.9904	117899722	111168991	2163397	2087490	1.037E+10	1.008E+10	144316344	100	30	91.76021413
T2_20880	247558318	0.9878	0.9772	0.9183	100	30	93	5	0.9441	13	0.9878	0.9772	0.9183	245496614	0.9917	121777542	121523398	1941530	1938863	1.148E+10	1.112E+10	136018362	100	30	92.77433956
T2_20881	317314472	0.9878	0.9772	0.9184	100	30	93	5	0.944	13	0.9878	0.9772	0.9184	314702664	0.9918	156120029	147785076	2462606	2356796	1.394E+10	1.354E+10	166244476	100	30	92.8010067
T2_20882	297639622	0.9878	0.9773	0.9181	100	30	93	5	0.9444	13	0.9878	0.9773	0.9181	295022584	0.9912	146271762	137889530	2479060	2364219	1.285E+10	1.285E+10	170954761	100	30	93.0447498
T2_20884	213575342	0.9874	0.9753	0.909	100	30	92	6	0.9395	13	0.9874	0.9753	0.909	211180486	0.9888	104461506	99050054	2257474	2187831	9.078E+09	9.116E+09	154655949	100	30	91.61255964
T2_20885	263598574	0.9877	0.9766	0.915	100	30	93	5	0.9426	13	0.9877	0.9766	0.915	261037294	0.9903	129312317	115729502	2412660	2201176	1.076E+10	1.069E+10	155440000	100	30	92.46573129
T2_20887	164833164	0.9876	0.9765	0.914	100	30	92	5	0.9423	13	0.9876	0.9765	0.914	163216843	0.9902	80846112	53826535	1524619	1142843	4.952E+09	4.939E+09	80187070	100	30	91.65082984
T2_20888	218805890	0.9877	0.9768	0.9166	100	30	93	5	0.9432	13	0.9877	0.9768	0.9166	216714270	0.9904	107372312	105404648	1969646	1945783	9.846E+09	9.731E+09	137745397	100	30	92.66629119
T2_20895	250959810	0.9875	0.9755	0.9098	100	30	92	6	0.94	13	0.9875	0.9755	0.9098	248223004	0.9891	122823004	117631837	2577094	2498829	1.08E+10	1.084E+10	175010770	100	30	91.74923575
T2_20896	294557764	0.9878	0.9775	0.9187	100	30	93	5	0.9447	13	0.9878	0.9775	0.9187	292024567	0.9914	144810004	141830987	2404559	2374856	1.322E+10	1.324E+10	173287634	100	30	93.14122223
T2_20897	289792604	0.9879	0.9777	0.9198	100	30	93	5	0.9452	13	0.9879	0.9777	0.9198	287287343	0.9914	142461741	121559349	2363861	2120495	1.134E+10	1.13E+10	149853127	100	30	92.93703493
T2_20898	163975014	0.9768	0.976	0.9656	62	30	43	34	0.9907	20	0.9768	0.976	0.9656	125965259	0.7682	54973997	54929304	16017265	16008427	2.386E+09	2.426E+09	640542409	62	30	43.32017433
T2_20899	234658420	0.9765	0.9757	0.9641	62	30	43	35	0.9908	20	0.9765	0.9757	0.9641	168612784	0.7185	71254092	70235619	26104600	25904889	3.02E+09	3.085E+09	1.025E+09	62	30	42.85457135
T3_20800	202907922	0.9764	0.9756	0.9636	62	30	43	35	0.9907	20	0.9764	0.9756	0.9636	141143529	0.6956	58745090	56716425	23653349	23049173	2.436E+09	2.483E+09	909879251	62	30	42.70578759
T3_20801	204578822	0.9763	0.9755	0.9631	62	30	43	35	0.9907	20	0.9763	0.9755	0.9631	139337202	0.6811	57718492	55410524	23900218	23245850	2.374E+09	2.42E+09	911680369	62	30	42.55787891
T3_20802	199192786	0.9765	0.9757	0.964	62	30	43	35	0.9908	20	0.9765	0.9757	0.964	142476122	0.7153	60243908	60017238	21988306	21946113	2.579E+09	2.635E+09	867096083	62	30	42.83140412
T3_20803	154784208	0.9763	0.9755	0.9632	62	30	43	35	0.9907	20	0.9763	0.9755	0.9632	104934790	0.6779	43438229	42712406	18058332	17880648	1.83E+09	1.867E+09	701610724	62	30	42.57321846
T3_20804	209340816	0.9765	0.9756	0.9637	63	30	43	35	0.9907	20	0.9765	0.9756	0.9637	149013449	0.7118	62349903	61838111	24313643	24202235	2.666E+09	2.71E+09	959715427	63	30	42.84509328
T3_20806	197368568	0.9765	0.9756	0.9638	62	30	43	35	0.9907	20	0.9765	0.9756	0.9638	139711998	0.7079	59192809	58174032	21326380	21118569	2.499E+09	2.555E+09	830057912	62	30	42.80085335
T3_20808	220596514	0.9771	0.9764	0.9658	63	30	44	32	0.9907	20	0.9771	0.9764	0.9658	166986845	0.757	72750645	72591146	21485555	21458547	3.209E+09	3.244E+09	857167827	63	30	43.86887108
T3_20809	215141054	0.977	0.9763	0.9654	63	30	44	32	0.9907	20	0.977	0.9763	0.9654	158695592	0.7376	69074891	66756061	20545810	20146707	2.936E+09	2.978E+09	795769467	63	30	43.66198464
T3_20810	261045068	0.977	0.9764	0.9656	62	30	44	33	0.9907	20	0.977	0.9764	0.9656	195277006	0.7481	84979709	75617838	25317588	23151702	3.327E+09	3.372E+09	918976999	62	30	43.68861394
T3_20811	233423152	0.977	0.9764	0.9657	63	30	44	33	0.9907	20	0.977	0.9764	0.9657	178507583	0.7647	78569924	72833759	21367735	20202595	3.191E+09	3.26E+09	803664355	63	30	43.73309165
T3_20812	176566632	0.9768	0.9761	0.9647	62	30	43	33	0.9907	20	0.9768	0.9761	0.9647	125349470	0.7099	53779669	46430303	17790132	15745024	2.018E+09	2.067E+09	620301253	62	30	43.32254088
T3_20813	149416042	0.9772	0.9766	0.9668	62	30	44	32	0.9907	20	0.9772	0.9766	0.9668	114611421	0.7671	50898738	50250764	12813945	12715662	2.223E+09	2.257E+09	505772118	62	30	44.0458948
T3_20814	272101000	0.9772	0.9766	0.9664	63	30	44	32	0.9907	20	0.9772	0.9766	0.9664	211448758	0.7771	93360396	83529955	24727966	23231621	3.691E+09	3.735E+09	928403887	63	30	43.90056708
T3_20815	271419086	0.9771	0.9765	0.9661	62	30	44	32	0.9907	20	0.9771	0.9765	0.9661	207865204	0.7658	91033906	90278001	25797392	25668466	3.995E+09	4.033E+09	1.026E+09	62	30	43.90203614
T3_20816	223644624	0.9773	0.9767	0.9666	63	30	44	32	0.9907	20	0.9773	0.9767	0.9666	174813441	0.7817	76985131	65864451	20843179	18543581	2.953E+09	2.938E+09	740488800	63	30	44.13302043
T3_20817	192177894	0.9772	0.9766	0.9666	62	30	44	32	0.9907	20	0.9772	0.9766	0.9666	148893969	0.7748	65473976	63399906	17946017	17554431	2.833E+09	2.829E+09	699721073	62	30	44.07033745
T3_20818	198304924	0.9771	0.9765	0.9662	63	30	44	32	0.9907	20	0.9771	0.9765	0.9662	148315921	0.7479	64470539	61175127	19374843	18883921	2.726E+09	2.721E+09	745426251	63	30	43.84162036
T3_20819	154618850	0.977	0.9764	0.9655	63	30	44	32	0.9907	20	0.977	0.9764	0.9655	112988355	0.7308	48496089	38675861	15996177	13239877	1.721E+09	1.712E+09	521894686	63	30	43.65249358
T3_20820	260584774	0.9773	0.9767	0.9671	63	30	44	32	0.9907	20	0.9773	0.9767	0.9671	207577715	0.7966	92394810	92128930	22788095	22757694	4.128E+09	4.122E+09	909636704	63	30	44.24432234
T3_20832	193067038	0.9772	0.9765	0.9662	62	30	44	32	0.9907	20	0.9772	0.9765	0.9662	146713209	0.7599	64077145	57099219	18558919	17066905	2.549E+09	2.544E+09	676031583	62	30	43.95405653
T3_20842	293780376	0.9774	0.9768	0.9673	62	30	44	31	0.9907	20	0.9774	0.9768	0.9673	235691254	0.8023	104725802	104467224	26239650	26199298	4.702E+09	4.678E+09	1.056E+09	62	30	44.38480922
T3_20843	241251956	0.9772	0.9767	0.9667	63	30	44	32	0.9907	20	0.9772	0.9767	0.9667	187105349	0.7756	82544042	79388077	22017265	21438392	3.556E+09	3.544E+09	849632854	63	30	44.11039429
T3_20845	195987526	0.9883	0.9798	0.9276	100	30	95	4	0.9502	14	0.9883	0.9798	0.9276	194643496	0.9931	96675045	94068787	1293406	1271688	8.815E+09	8.985E+09	97296464	100	30	94.48799747
T3_20846	163664950	0.9882	0.9792	0.9243	100	30	94	4	0.9486	14	0.9882	0.9792	0.9243	162547056	0.9932	80733809	76845737	1079438	1050166	7.168E+09	7.321E+09	80443311	100	30	94.15787003
T3_20847	242551680	0.9884	0.9803	0.9305	100	30	95	3	0.9515	14	0.9884	0.9803	0.9305	241036686	0.9938	119788173	119547185	1460340	1458496	1.127E+10	1.148E+10	113850341	100	30	95.05835518
T3_20849	181912182	0.9882	0.9791	0.9243	100	30	94	4	0.9483	14	0.9882	0.9791	0.9243	180574987	0.9926	89643055	89242854	1288877	1286215	8.314E+09	8.533E+09	99397325	100	30	94.26362275
T3_20850	204280650	0.9885	0.9809	0.9333	100	30	95	3	0.953	14	0.9885	0.9809	0.9333	203122918	0.9943	101007053	86254144	1108812	981275	8.207E+09	8.221E+09	73855836	100	30	95.1186551
T3_20851	198435968	0.9882	0.9791	0.9226	100	30	94	4	0.9483	14	0.9882	0.9791	0.9226	196991705	0.9927	97803082	96352508	1385541	1370642	9.008E+09	9.143E+09	102043337	100	30	94.0532355
T3_20853	236115154	0.9883	0.9794	0.9257	100	30	94	4	0.9491	14	0.9883	0.9794	0.9257	234380418	0.9927	116353686	115687225	1673046	1668614	1.079E+10	1.107E+10	129544223	100	30	94.36465175
T3_20854	195723164	0.9884	0.9802	0.9296	100	30	95	4	0.9512	14	0.9884	0.9802	0.9296	194479382	0.9936	96639465	96515117	1200452	1199639	9.08E+09	9.258E+09	93546478	100	30	94.89709327
T3_20855	337593996	0.9875	0.9756	0.9097	100	30	91	7	0.9405	13	0.9875	0.9756	0.9097	333641859	0.9883	164969409	154867673	3703041	3522329	1.431E+10	1.392E+10	244802643	100	30	90.9202444
T3_20857	316404958	0.9875	0.9758	0.9113	100	30	91	6	0.9409	13	0.9875	0.9758	0.9113	312809277	0.9886	154713846	153710435	3381585	3369203	1.424E+10	1.392E+10	239071533	100	30	91.3591307
T3_20859	262208604	0.9875	0.9757	0.911	100	30	91	6	0.9408	13	0.9875	0.9757	0.911	259271288	0.9888	128257509	127145880	2756270	2743351	1.181E+10	1.148E+10	192718506	100	30	91.33297093
T3_20860	267398194	0.9874	0.9749	0.907	100	30	91	7	0.9388	13	0.9874	0.9749	0.907	264097945	0.9877	130499171	123864342	3099603	2982107	1.139E+10	1.113E+10	209732160	100	30	90.64568614
T3_20861	223697584	0.9875	0.9754	0.9092	100	30	91	7	0.9401	13	0.9875	0.9754	0.9092	221019935	0.988	109254127	82981253	2511681	2023817	7.632E+09	7.429E+09	141420902	100	30	90.4984701
T3_20862	297459466	0.9876	0.9759	0.9114	100	30	91	6	0.9411	13	0.9876	0.9759	0.9114	294081973	0.9886	145449685	128294465	3182603	2902882	1.182E+10	1.165E+10	207431741	100	30	91.24349647
T3_20863	345033832	0.9878	0.9772	0.9178	100	30	92	6	0.9443	13	0.9878	0.9772	0.9178	341655014	0.9902	169236814	168662121	3181386	3175251	1.582E+10	1.534E+10	226538921	100	30	92.19198197
T3_20864	286043948	0.9876	0.9762	0.9132	100	30	91	6	0.942	13	0.9876	0.9762	0.9132	282866018	0.9889	139938818	118136666	2988382	2592200	1.095E+10	1.069E+10	184127480	100	30	91.40303332
T3_20865	470831064	0.9879	0.9776	0.9204	100	30	93	5	0.9449	13	0.9879	0.9776	0.9204	466263170	0.9903	230977322	230444720	4308526	4297935	2.161E+10	2.131E+10	310736220	100	30	92.92819064
T3_20866	285680720	0.9874	0.9751	0.9089	100	30	91	6	0.939	13	0.9874	0.9751	0.9089	282030124	0.9872	139300883	132563215	3428358	3319261	1.216E+10	1.207E+10	234793819	100	30	91.10484975
T3_20867	313248772	0.9876	0.9761	0.9131	100	30	92	6	0.9414	13	0.9876	0.9761	0.9131	309709287	0.9887	153187864	147094489	3333559	3233288	1.366E+10	1.338E+10	231387687	100	30	91.71718878
T3_20868	384004686	0.9876	0.9761	0.9136	100	30	92	6	0.9415	13	0.9876	0.9761	0.9136	379741838	0.9889	187862646	187222103	4016546	4004428	1.739E+10	1.711E+10	287026230	100	30	91.93286199
T3_20869	275168684	0.9876	0.976	0.9121	100	30	91	6	0.941	13	0.9876	0.976	0.9121	271874370	0.988	134391904	133306180	3090562	3071668	1.235E+10	1.21E+10	216215449	100	30	91.46440331
T3_20870	311293840	0.9874	0.9751	0.9088	100	30	91	6	0.9391	13	0.9874	0.9751	0.9088	307400348	0.9875	151869365	146845591	3661618	3578837	1.35E+10	1.334E+10	253926055	100	30	91.16278667
T3_20871	263382654	0.9875	0.9755	0.9109	100	30	92	6	0.94	13	0.9875	0.9755	0.9109	260173290	0.9878	128573937	126220133	3025416	2995454	1.161E+10	1.157E+10	215545934	100	30	91.58721052
T3_20872	259267326	0.9875	0.9752	0.9097	100	30	91	6	0.9394	13	0.9875	0.9752	0.9097	256019288	0.9875	126485902	123290730	3047484	3002098	1.135E+10	1.122E+10	211687258	100	30	91.27887963
T3_20873	195896388	0.9766	0.9757	0.9645	62	30	43	35	0.9907	20	0.9766	0.9757	0.9645	140466700	0.717	60450181	59925867	19566338	19461485	2.577E+09	2.644E+09	764076366	62	30	42.96547956
T3_20874	176900978	0.9764	0.9755	0.9632	62	30	43	35	0.9907	20	0.9764	0.9755	0.9632	119819096	0.6773	50164423	48739831	19490250	19123654	2.09E+09	2.138E+09	745818507	62	30	42.66183469
T3_20875	140061164	0.9764	0.9755	0.9633	62	30	43	35	0.9907	20	0.9764	0.9755	0.9633	94687166	0.676	39365347	39206808	15956472	15918777	1.685E+09	1.718E+09	623667665	62	30	42.68517177
T3_20876	162409222	0.9766	0.9758	0.9647	62	30	43	34	0.9907	20	0.9766	0.9758	0.9647	117613721	0.7242	50374279	50291886	16865163	16850505	2.168E+09	2.218E+09	666072288	62	30	43.01782176
T3_20877	137133026	0.9764	0.9755	0.9636	62	30	43	35	0.9908	20	0.9764	0.9755	0.9636	92726113	0.6762	38988375	36556258	14749363	14168404	1.562E+09	1.605E+09	552790737	62	30	42.61479179
T3_20878	186901948	0.9765	0.9756	0.9639	63	30	43	35	0.9908	20	0.9765	0.9756	0.9639	130870050	0.7002	55572098	45709984	19725854	16941753	1.958E+09	2.009E+09	663175646	63	30	42.72514822
T3_20879	198616954	0.9765	0.9756	0.9638	63	30	43	35	0.9908	20	0.9765	0.9756	0.9638	138530288	0.6975	58723424	53342892	21083440	19796519	2.285E+09	2.345E+09	775283326	63	30	42.73888772
T3_20880	170774698	0.9765	0.9757	0.9641	62	30	43	35	0.9907	20	0.9765	0.9757	0.9641	120563778	0.706	51234965	50926864	18093848	18024058	2.186E+09	2.241E+09	708124551	62	30	42.83918513
T3_20881	290812022	0.9884	0.9801	0.9265	100	30	95	4	0.9507	14	0.9884	0.9801	0.9265	289139742	0.9942	143762495	140922255	1614752	1588571	1.325E+10	1.342E+10	121058517	100	30	94.53753683
T3_20882	159580634	0.9883	0.9795	0.9238	100	30	94	4	0.9493	14	0.9883	0.9795	0.9238	158603574	0.9939	78831398	71711761	940778	886496	6.722E+09	6.767E+09	65604713	100	30	93.93153103
T3_20884	226957182	0.9884	0.98	0.9262	100	30	94	4	0.9505	14	0.9884	0.98	0.9262	225604839	0.994	112151521	90427434	1301797	1165018	8.8E+09	8.923E+09	87094464	100	30	94.12117202
T3_20885	159292790	0.9882	0.979	0.9212	100	30	94	5	0.9479	14	0.9882	0.979	0.9212	158237843	0.9934	78609552	71479840	1018739	952498	6.645E+09	6.763E+09	71313572	100	30	93.6637762
T3_20887	284034802	0.9883	0.9791	0.9222	100	30	94	5	0.9482	14	0.9883	0.9791	0.9222	282049519	0.993	140069400	139462005	1910719	1906683	1.299E+10	1.322E+10	141562426	100	30	93.82939129
T3_20888	338230964	0.9886	0.9815	0.9343	100	30	95	3	0.9544	14	0.9886	0.9815	0.9343	336624623	0.9953	167538423	166499472	1547777	1542429	1.588E+10	1.589E+10	116627982	100	30	95.30493141
T3_20895	227467966	0.9883	0.9792	0.9224	100	30	94	5	0.9485	14	0.9883	0.9792	0.9224	225924939	0.9932	112220843	107234944	1483253	1442563	1E+10	1.014E+10	106574782	100	30	93.79454667
T3_20896	219642818	0.9882	0.9789	0.9208	100	30	94	5	0.9478	14	0.9882	0.9789	0.9208	218141191	0.9932	108347065	105098954	1447061	1422480	9.792E+09	9.932E+09	106039503	100	30	93.70703774
T3_20897	141120248	0.9881	0.9782	0.919	100	30	93	6	0.9465	13	0.9881	0.9782	0.919	139842536	0.9909	69309639	68422793	1223258	1216597	6.349E+09	6.339E+09	88776395	100	30	92.53811349
T3_20898	155503208	0.9881	0.9783	0.9194	100	30	93	5	0.9465	13	0.9881	0.9783	0.9194	154111340	0.991	76386491	76298646	1338358	1337449	7.08E+09	7.096E+09	99813793	100	30	92.74367229
T3_20899	179881892	0.9882	0.979	0.9228	100	30	93	5	0.9483	14	0.9882	0.979	0.9228	178424681	0.9919	88513520	77596477	1397641	1276549	7.235E+09	7.208E+09	93944325	100	30	92.90186488
T4_20800	257208312	0.9883	0.9796	0.9244	100	30	94	4	0.9496	14	0.9883	0.9796	0.9244	255661003	0.994	127085888	126069331	1489227	1482178	1.183E+10	1.196E+10	109916353	100	30	94.21869267
T4_20801	272977468	0.9884	0.98	0.9262	100	30	94	4	0.9506	14	0.9884	0.98	0.9262	271413566	0.9943	134954302	134889960	1504962	1504583	1.271E+10	1.281E+10	111764297	100	30	94.47900052
T4_20802	176499418	0.9883	0.9794	0.9244	100	30	94	4	0.9492	14	0.9883	0.9794	0.9244	175332669	0.9934	87107225	85987322	1118219	1110939	8.073E+09	8.1E+09	80236614	100	30	93.90438905
T4_20803	133040138	0.9883	0.9791	0.9222	100	30	94	5	0.9483	14	0.9883	0.9791	0.9222	132168914	0.9935	65665735	63986629	837444	826820	5.983E+09	6.03E+09	60308816	100	30	93.73697096
T4_20804	279739290	0.9885	0.9804	0.929	100	30	95	4	0.9515	14	0.9885	0.9804	0.929	278193997	0.9945	138351901	125728461	1490195	1365597	1.187E+10	1.199E+10	103987939	100	30	94.78110656
T4_20806	179585574	0.9884	0.9799	0.9257	100	30	94	4	0.9501	14	0.9884	0.9799	0.9257	178516207	0.994	88742854	81133486	1030499	958763	7.615E+09	7.708E+09	72287251	100	30	94.32062075
T4_20808	248438352	0.9883	0.9793	0.9228	100	30	94	4	0.9488	14	0.9883	0.9793	0.9228	246876447	0.9937	122687048	121543753	1502351	1493080	1.139E+10	1.15E+10	109856099	100	30	94.01237615
T4_20809	263691928	0.9883	0.9796	0.926	100	30	94	5	0.9497	14	0.9883	0.9796	0.926	261655796	0.9923	129846998	126206932	1961800	1928161	1.182E+10	1.184E+10	146463406	100	30	93.58987009
T4_20810	206814900	0.9883	0.9797	0.9265	100	30	94	5	0.95	14	0.9883	0.9797	0.9265	205241278	0.9924	101864873	99422166	1511532	1490898	9.339E+09	9.297E+09	111161461	100	30	93.57774029
T4_20811	225639776	0.9884	0.9801	0.9283	100	30	94	5	0.951	14	0.9884	0.9801	0.9283	224026458	0.9929	111236754	110071960	1552950	1544774	1.036E+10	1.034E+10	116702846	100	30	93.91313524
T4_20812	234066758	0.9882	0.9789	0.9224	100	30	93	5	0.948	14	0.9882	0.9789	0.9224	232111811	0.9916	115116267	106237978	1879277	1768425	9.898E+09	9.913E+09	132439726	100	30	93.08855561
T4_20813	175589214	0.988	0.9781	0.9188	100	30	93	6	0.9461	13	0.988	0.9781	0.9188	173982061	0.9908	86222253	85958235	1537555	1535323	7.99E+09	7.95E+09	111508054	100	30	92.54508132
T4_20814	225303718	0.9886	0.9814	0.9336	100	30	95	3	0.9541	14	0.9886	0.9814	0.9336	224219798	0.9952	111586112	111367839	1047574	1046367	1.058E+10	1.066E+10	80073422	100	30	95.30089874
T4_20815	168828094	0.9766	0.9759	0.9653	62	30	43	34	0.9907	20	0.9766	0.9759	0.9653	124093369	0.735	53915405	51176151	16262559	15769214	2.213E+09	2.259E+09	620840519	62	30	43.11512671
T4_20816	90996022	0.9765	0.9757	0.9641	62	30	43	35	0.9907	20	0.9765	0.9757	0.9641	64111057	0.7045	27410370	23145613	9290317	8240382	994350872	1.018E+09	321447452	62	30	42.8002815
T4_20817	232003956	0.9765	0.9756	0.9639	62	30	43	35	0.9907	20	0.9765	0.9756	0.9639	162160337	0.699	68826788	67547055	24506761	24259212	2.901E+09	2.971E+09	949272642	62	30	42.80715238
T4_20818	261981430	0.9767	0.976	0.9656	62	30	43	34	0.9907	20	0.9767	0.976	0.9656	197833948	0.7551	86356970	64773500	25120008	21440160	2.799E+09	2.861E+09	848217178	62	30	43.09999385
T4_20819	235158088	0.9766	0.9757	0.9644	62	30	43	34	0.9907	20	0.9766	0.9757	0.9644	167065885	0.7104	71822891	71566673	23420103	23375784	3.081E+09	3.16E+09	914755002	62	30	42.97602776
T4_20820	49627942	0.9765	0.9757	0.964	62	30	43	34	0.9907	20	0.9765	0.9757	0.964	35010251	0.7055	14857624	14435117	5295003	5195450	621692449	635740307	204152689	62	30	42.90491995
T4_20832	245314530	0.9764	0.9755	0.9633	62	30	43	35	0.9907	20	0.9764	0.9755	0.9633	169914817	0.6926	71257046	69789604	27400725	27092705	3.004E+09	3.06E+09	1.061E+09	62	30	42.74560057
T4_20842	107722484	0.9766	0.9758	0.9645	62	30	43	34	0.9907	20	0.9766	0.9758	0.9645	79027183	0.7336	34050558	34022287	10926067	10920647	1.467E+09	1.501E+09	430671996	62	30	43.04232903
T4_20843	299355732	0.9769	0.9761	0.9648	62	30	44	33	0.9907	20	0.9769	0.9761	0.9648	221910256	0.7413	95035655	94972644	31838946	31829342	4.176E+09	4.214E+09	1.267E+09	62	30	43.54608744
T4_20845	83805148	0.9769	0.9761	0.9648	63	30	44	33	0.9907	20	0.9769	0.9761	0.9648	62159316	0.7417	26644372	26590614	8870572	8862764	1.169E+09	1.18E+09	352582015	63	30	43.5473731
T4_20846	307698476	0.977	0.9762	0.9654	62	30	44	33	0.9907	20	0.977	0.9762	0.9654	234508614	0.7621	102011628	100827083	30485358	30249408	4.438E+09	4.493E+09	1.206E+09	62	30	43.71238423
T4_20847	101639276	0.9768	0.9761	0.9649	62	30	43	33	0.9907	20	0.9768	0.9761	0.9649	73963958	0.7277	31997582	31910504	9968794	9953734	1.393E+09	1.422E+09	391883788	62	30	43.47249144
T4_20849	252099230	0.9768	0.976	0.9646	62	30	43	33	0.9907	20	0.9768	0.976	0.9646	184794440	0.733	79671220	79266956	25452000	25378281	3.465E+09	3.53E+09	1.003E+09	62	30	43.49054598
T4_20850	180743238	0.977	0.9762	0.9656	62	30	44	32	0.9907	20	0.977	0.9762	0.9656	137603166	0.7613	60065530	59382333	17472106	17315637	2.61E+09	2.652E+09	688033975	62	30	43.72696828
T4_20851	291453560	0.9769	0.9762	0.965	63	30	44	33	0.9907	20	0.9769	0.9762	0.965	217733019	0.7471	93990311	92593841	29752397	29448576	4.074E+09	4.109E+09	1.166E+09	63	30	43.55852891
T4_20853	90258970	0.9769	0.9761	0.9649	62	30	44	33	0.9907	20	0.9769	0.9761	0.9649	67215183	0.7447	28942669	27529832	9329845	9067871	1.21E+09	1.222E+09	360228456	62	30	43.53807694
T4_20854	234336192	0.9764	0.9755	0.9631	62	30	43	35	0.9907	20	0.9764	0.9755	0.9631	161806405	0.6905	66958416	65459748	27889573	27497031	2.807E+09	2.86E+09	1.084E+09	62	30	42.6195923
T4_20855	165051004	0.9764	0.9756	0.9637	62	30	43	35	0.9908	20	0.9764	0.9756	0.9637	117312903	0.7108	49513764	47098007	18285375	17528162	2.018E+09	2.067E+09	691763647	62	30	42.75219832
T4_20857	255408086	0.9765	0.9757	0.964	62	30	43	35	0.9908	20	0.9765	0.9757	0.964	184532043	0.7225	78084456	77924751	28363131	28333688	3.348E+09	3.421E+09	1.122E+09	62	30	42.84243262
T4_20859	72881212	0.9764	0.9755	0.9631	62	30	43	35	0.9907	20	0.9764	0.9755	0.9631	50409414	0.6917	20953667	20481335	8502080	8365697	879133672	895126249	329197774	62	30	42.6419487
T4_20860	245041174	0.9765	0.9757	0.9646	62	30	43	35	0.9908	20	0.9765	0.9757	0.9646	178439946	0.7282	76609144	76269406	25221658	25146495	3.272E+09	3.356E+09	993614200	62	30	42.89004466
T4_20861	267245796	0.9765	0.9756	0.964	62	30	43	35	0.9907	20	0.9765	0.9756	0.964	190773294	0.7138	81169655	71028953	28433984	26153001	3.043E+09	3.117E+09	1.027E+09	62	30	42.72608886
T4_20862	336823670	0.9765	0.9757	0.9642	63	30	43	35	0.9908	20	0.9765	0.9757	0.9642	246286930	0.7312	104396861	103114245	37493208	37216978	4.442E+09	4.528E+09	1.481E+09	63	30	42.92869944
T4_20863	120119608	0.9766	0.9758	0.9647	62	30	43	35	0.9908	20	0.9766	0.9758	0.9647	89401901	0.7443	38468656	36010613	12464589	11836384	1.551E+09	1.584E+09	469517079	62	30	42.99141857
T4_20864	231490924	0.9772	0.9766	0.9668	63	30	44	33	0.9907	20	0.9772	0.9766	0.9668	184393396	0.7965	82100059	81713777	20193278	20136695	3.625E+09	3.653E+09	803551193	63	30	44.02455213
T4_20865	131056940	0.977	0.9764	0.9657	62	30	44	33	0.9907	20	0.977	0.9764	0.9657	98550913	0.752	42808480	42440055	12933953	12859315	1.87E+09	1.889E+09	508658224	62	30	43.65781055
T4_20866	203067852	0.9771	0.9765	0.9661	62	30	44	33	0.9907	20	0.9771	0.9765	0.9661	155154115	0.7641	68197933	53535575	18758249	15197748	2.367E+09	2.386E+09	600639307	62	30	43.78994936
T4_20867	167086282	0.9771	0.9764	0.9661	62	30	44	33	0.9907	20	0.9771	0.9764	0.9661	127021517	0.7602	55289238	54585115	16443041	16300538	2.414E+09	2.429E+09	648035458	62	30	43.76482936
T4_20868	254119710	0.9771	0.9765	0.9665	62	30	44	33	0.9907	20	0.9771	0.9765	0.9665	197629628	0.7777	87694120	87664073	22241388	22235755	3.857E+09	3.92E+09	882891305	62	30	43.83094952
T4_20869	260037098	0.977	0.9764	0.9655	62	30	44	34	0.9908	20	0.977	0.9764	0.9655	198029601	0.7615	87258114	87101373	23513373	23489255	3.817E+09	3.881E+09	920715082	62	30	43.60032438
T4_20870	275058972	0.9771	0.9765	0.9661	62	30	44	33	0.9907	20	0.9771	0.9765	0.9661	211349139	0.7684	93103221	83200050	25142697	23106662	3.677E+09	3.711E+09	912499540	62	30	43.79749531
T4_20871	183359396	0.9771	0.9764	0.9659	62	30	44	33	0.9907	20	0.9771	0.9764	0.9659	140389646	0.7657	61633755	56542186	17122136	16072541	2.496E+09	2.52E+09	635327146	62	30	43.75744385
T4_20872	129914136	0.9884	0.9797	0.9254	100	30	94	4	0.9496	14	0.9884	0.9797	0.9254	128996649	0.9929	64059448	54956880	877753	785868	5.199E+09	5.142E+09	57710581	100	30	93.93919331
T4_20873	228664890	0.9883	0.9794	0.9242	100	30	94	4	0.949	14	0.9883	0.9794	0.9242	227047082	0.9929	112749408	112419476	1548266	1545990	1.066E+10	1.054E+10	113961248	100	30	94.13869514
T4_20874	247311506	0.9883	0.9795	0.9245	100	30	94	4	0.9491	14	0.9883	0.9795	0.9245	245596427	0.9931	121975375	120918896	1645677	1636656	1.148E+10	1.133E+10	122257977	100	30	94.21198933
T4_20875	245002270	0.9881	0.9781	0.9176	100	30	93	5	0.9457	13	0.9881	0.9781	0.9176	242943987	0.9916	120487255	114966101	1969477	1908472	1.082E+10	1.064E+10	140098067	100	30	93.15990933
T4_20876	259098674	0.9883	0.9794	0.9235	100	30	94	4	0.9488	14	0.9883	0.9794	0.9235	257243735	0.9928	127735036	126429519	1773663	1765280	1.195E+10	1.185E+10	129555394	100	30	93.9666875
T4_20877	279095546	0.9881	0.9782	0.9173	100	30	93	5	0.9457	13	0.9881	0.9782	0.9173	276768990	0.9917	137272881	120592998	2223228	2000964	1.132E+10	1.116E+10	146738413	100	30	93.07238307
T4_20878	225105920	0.9881	0.9782	0.9171	100	30	93	5	0.9457	13	0.9881	0.9782	0.9171	223254875	0.9918	110741684	95792469	1771507	1597557	9.004E+09	8.845E+09	116958675	100	30	93.00143135
T4_20879	205894428	0.9883	0.9795	0.9241	100	30	94	4	0.9491	14	0.9883	0.9795	0.9241	204464262	0.9931	101548815	98885299	1366632	1349961	9.339E+09	9.275E+09	98528710	100	30	93.97588571
T4_20880	208484140	0.9883	0.979	0.9216	100	30	94	4	0.9478	14	0.9883	0.979	0.9216	206926877	0.9925	102715094	96129593	1496689	1434920	9.104E+09	8.942E+09	107566088	100	30	93.72296639
T4_20881	254798486	0.9882	0.9788	0.9209	100	30	94	4	0.9474	14	0.9882	0.9788	0.9209	252845664	0.9923	125485258	124405067	1875148	1865272	1.178E+10	1.158E+10	140145839	100	30	93.73073219
T4_20882	430019500	0.9883	0.9791	0.9226	100	30	94	4	0.9482	14	0.9883	0.9791	0.9226	426836009	0.9926	211892381	211258405	3051247	3046299	2.001E+10	1.974E+10	226559392	100	30	93.94054744
T4_20884	121945856	0.9884	0.9798	0.9262	100	30	94	4	0.95	14	0.9884	0.9798	0.9262	121129023	0.9933	60173644	59441673	781735	774081	5.616E+09	5.603E+09	57588410	100	30	94.24388313
T4_20885	275853662	0.9884	0.9797	0.9258	100	30	94	4	0.9497	14	0.9884	0.9797	0.9258	274011884	0.9933	136125254	134042912	1761376	1744699	1.267E+10	1.264E+10	128749407	100	30	94.26919468
T4_20887	141068256	0.9882	0.979	0.9221	100	30	94	5	0.9478	14	0.9882	0.979	0.9221	139997759	0.9924	69487294	69185697	1023171	1021336	6.531E+09	6.461E+09	74435692	100	30	93.74031957
T4_20888	184612210	0.9884	0.98	0.9262	100	30	94	4	0.9502	14	0.9884	0.98	0.9262	183373443	0.9933	91092109	90941473	1189225	1188239	8.629E+09	8.549E+09	89427143	100	30	94.31754947
T4_20895	173775878	0.9884	0.9798	0.9259	100	30	94	4	0.9499	14	0.9884	0.9798	0.9259	172606160	0.9933	85741998	85328275	1122164	1119117	8.128E+09	8.007E+09	84480952	100	30	94.42146963
T4_20896	158892276	0.9881	0.9786	0.9226	100	30	94	4	0.9464	14	0.9881	0.9786	0.9226	157257516	0.9897	77822717	75202485	1612082	1573316	7.259E+09	6.855E+09	137421847	100	30	93.77342823
T4_20897	120530344	0.9881	0.9787	0.9233	100	30	94	4	0.9466	14	0.9881	0.9787	0.9233	119259014	0.9895	59003505	58857959	1252004	1250045	5.67E+09	5.377E+09	108420903	100	30	93.77377065
T4_20898	189961472	0.9881	0.9791	0.9254	100	30	94	3	0.9477	14	0.9881	0.9791	0.9254	188121515	0.9903	93154058	92522610	1813399	1805425	8.943E+09	8.489E+09	157931516	100	30	94.14191994
T4_20899	176617424	0.9883	0.9802	0.9317	100	30	95	3	0.9506	14	0.9883	0.9802	0.9317	175140666	0.9916	86842039	86231735	1456588	1448917	8.379E+09	8.009E+09	128444073	100	30	94.96954141

Supplementary Table 4 | The PCOA results of the two highest proportion principal components.

Sample_ID	time	group	Genus.Axis.1	Genus.Axis.2	Species.Axis	Species.Axis.2
T1_20800	T1	Model	-0.1733908	-0.052973285	-0.183212	-0.05674596
T1_20801	T1	Model	-0.1671718	-0.055940571	-0.197314	-0.043527597
T1_20802	T4	Normal	-0.3166624	0.045709419	0.2207199	0.145601866
T1_20803	T1	Normal	-0.2870513	0.031379312	0.1782416	-0.181317035
T1_20804	T4	Model	0.08165466	-0.143031892	-0.186356	-0.042673525
T1_20806	T4	Model	-0.280948	0.017497139	-0.048783	-0.099081018
T1_20808	T1	Model	-0.1849969	-0.036961948	-0.324982	0.035424456
T1_20809	T4	Model	-0.2180541	-0.024438292	0.2228594	-0.207856786
T1_20810	T4	Model	-0.1697742	-0.051753041	0.3267801	0.41903784
T1_20811	T4	Model	-0.3209445	0.045217152	-0.186849	-0.031991846
T1_20812	T4	Normal	0.19175615	-0.204427882	-0.13082	0.062315147
T1_20813	T4	Model	-0.1697136	-0.042887451	0.3845191	-0.234363337
T1_20814	T4	Model	-0.2621509	0.015476525	-0.128418	-0.0405557
T1_20815	T4	Normal	-0.2470279	0.007657213	-0.205015	0.064565327
T1_20816	T4	Model	-0.1351513	-0.069900151	-0.170415	0.147129321
T1_20817	T4	Model	-0.2070708	-0.035956335	-0.248294	0.037615846
T1_20818	T4	Model	0.20193959	-0.211631413	0.1956507	0.279312429
T1_20819	T4	Normal	-0.1016322	-0.100838173	0.406292	-0.01607342
T1_20820	T4	Normal	-0.3173772	0.064935236	0.3828906	0.547623053
T1_20832	T1	Normal	-0.2786535	0.017536697	0.3997347	0.210878748
T1_20842	T1	Normal	-0.0753099	-0.05818711	0.442339	0.297045193
T1_20843	T1	Model	0.00663094	-0.133297837	-0.296974	0.019385766
T1_20845	T1	Normal	0.10888777	-0.17664766	0.3075378	0.012528006
T1_20846	T1	Normal	0.01574267	-0.149965193	-0.30296	0.05783664
T1_20847	T1	Normal	-0.2010158	-0.022370723	0.4269486	0.28755149
T1_20849	T1	Normal	-0.2139443	0.004452349	0.2922158	-0.201432925
T1_20850	T1	Normal	-0.1765715	-0.045137406	-0.112604	-0.086389211
T1_20851	T1	Normal	-0.3007795	0.041137732	-0.258864	0.043088705
T1_20853	T1	Normal	-0.228853	-0.005494794	-0.157092	0.012111983
T1_20854	T1	Normal	-0.2312131	-0.010760901	0.1152998	-0.131249673
T1_20855	T1	Normal	-0.2027671	-0.033473919	0.0995072	-0.168551445
T1_20857	T1	Normal	-0.2700799	0.055196463	0.262373	-0.194517809
T1_20859	T1	Normal	0.10402194	-0.178805045	0.0738564	-0.138460232
T1_20860	T1	Normal	0.08443389	0.058563439	0.2226911	-0.046052941
T1_20861	T1	Model	-0.2001206	0.101539073	0.0399963	-0.128900012
T1_20862	T1	Model	-0.0408207	0.34989549	-0.281159	0.000434943
T1_20863	T1	Model	-0.1148624	0.089667928	-0.21691	-0.026694932
T1_20864	T1	Model	0.30524981	0.150158539	-0.200739	-0.044336417
T1_20865	T1	Model	-0.1556746	0.185181796	-0.214051	-0.0272117
T1_20866	T1	Model	-0.0017308	-0.118361504	-0.343582	0.069396941
T1_20867	T1	Model	0.31118021	-0.167048576	0.2029009	-0.215742588
T1_20868	T1	Model	-0.0902379	-0.075865006	-0.173628	-0.060326027
T1_20869	T1	Model	-0.147839	-0.047462671	-0.288858	0.035743955
T1_20870	T1	Model	0.00163543	-0.087403175	-0.254423	-0.003786879
T1_20871	T1	Model	-0.0745684	0.219937485	-0.18064	-0.04809494
T1_20872	T1	Model	0.04688007	-0.151167837	-0.182962	-0.048999947
T1_20873	T1	Model	-0.1583143	-0.034994488	0.2231115	-0.215516397
T1_20874	T1	Model	0.32162076	0.360495277	-0.134301	-0.086720566
T1_20875	T1	Model	0.20437645	-0.192820384	-0.33615	0.084170342
T1_20876	T1	Model	0.10433972	-0.155598071	-0.262846	-0.008222138
T1_20877	T1	Model	-0.0149075	-0.007125379	-0.026756	-0.084341667
T1_20878	T1	Model	0.11122935	-0.154350208	0.0576491	-0.154503001
T1_20879	T1	Model	0.36773834	-0.159514147	0.2067912	-0.184990284
T1_20880	T1	Model	-0.0736147	-0.05987291	0.1173613	-0.175846998
T1_20881	T1	Model	-0.2775258	0.013976333	-0.166009	-0.049430943
T1_20882	T1	Model	-0.3225284	0.090782681	-0.215953	-0.015393647
T1_20884	T1	Model	-0.0480824	-0.052292559	-0.192491	-0.055037566
T1_20885	T1	Model	0.04013256	-0.151080007	-0.311611	0.032960863
T1_20887	T1	Model	-0.0538837	-0.103533902	-0.226953	-0.021491842
T1_20888	T1	Model	0.19765896	-0.205805526	-0.237906	-0.017311113
T1_20895	T1	Model	0.26266903	-0.201807815	-0.202719	-0.03923055
T1_20896	T1	Model	0.03854335	-0.1288114	-0.2092	0.016667979
T1_20897	T1	Model	0.27646758	-0.027721457	0.094393	-0.173668618
T1_20898	T1	Model	-0.2419804	0.03617284	0.2232163	0.060213603
T1_20899	T1	Model	0.19855467	-0.186235187	-0.227284	0.100993743
T2_20800	T1	Model	0.08691172	-0.175685177	0.0663257	0.350598221
T2_20801	T1	Model	-0.2933253	0.145873485	-0.099204	0.074046256
T2_20802	T4	Normal	-0.2781075	0.102797889	0.0330919	-0.087035781
T2_20803	T1	Model	-0.2063304	0.145351059	0.3095464	0.157632108
T2_20804	T4	Model	-0.1994043	-0.020299787	-0.173082	0.016096172
T2_20806	T4	Model	-0.0765378	-0.088315339	0.0003225	-0.065517228
T2_20808	T1	Model	0.33829167	0.40082428	-0.146846	0.176005453
T2_20809	T4	Model	-0.2701852	0.015495833	-0.213245	-0.016740674
T2_20810	T4	Normal	0.04363971	-0.09066196	-0.262777	0.028813904
T2_20811	T4	Model	-0.1551169	0.005981464	-0.184407	-0.019746083
T2_20812	T4	Normal	0.38347289	-0.251105921	0.3373138	0.096633565
T2_20813	T4	Model	0.28287938	-0.179566429	-0.250614	0.027989354
T2_20814	T4	Model	0.43063547	-0.240366396	-0.17095	-0.016576819
T2_20815	T4	Normal	-0.209731	0.006537546	-0.236244	0.174426618
T2_20816	T4	Model	-0.1746322	-0.03924742	-0.14683	-0.047649715
T2_20817	T4	Model	0.2011538	-0.202522978	-0.241197	0.005497855
T2_20818	T4	Model	-0.0195496	0.080035317	-0.216797	-0.001111376
T2_20819	T4	Normal	0.33728158	-0.228824067	-0.148332	0.081111263
T2_20820	T4	Normal	-0.2094279	0.17385086	-0.195211	-0.007968329
T2_20832	T1	Model	0.19079683	0.185137168	-0.012057	-0.11928775
T2_20842	T1	Model	-0.2071618	-0.013090878	0.3288424	-0.165186229
T2_20843	T1	Model	-0.2256327	-0.009909236	-0.105799	-0.081482532
T2_20845	T1	Model	0.21121101	-0.216147918	-0.143938	-0.05753805
T2_20846	T1	Model	0.45343305	0.382826541	0.0659852	-0.10509407
T2_20847	T1	Model	-0.2128875	-0.015370381	-0.082558	0.223222048
T2_20849	T1	Model	0.08736827	-0.063693305	0.0857671	-0.162476145
T2_20850	T1	Model	-0.2315488	0.190848803	-0.154677	-0.047811161
T2_20851	T1	Model	0.36397822	0.511444353	0.2994527	0.372830512
T2_20853	T1	Model	0.168531	0.020858796	0.1859024	-0.186376204
T2_20854	T1	Model	0.15457232	0.039095601	0.1041146	-0.153353994
T2_20855	T2	Model	0.13576806	0.262673403	-0.030258	0.000441968
T2_20857	T2	Model	-0.1320171	0.018941111	0.0917126	-0.149433844
T2_20859	T2	Normal	0.36221215	-0.20382022	-0.285464	0.035207394
T2_20860	T2	Model	0.07564685	-0.178976363	0.3514796	-0.147881411
T2_20861	T2	Normal	-0.2396591	0.016502547	-0.161725	-0.045311448
T2_20862	T2	Normal	-0.0447827	-0.102943076	-0.129017	0.032213678
T2_20863	T2	Model	0.34110013	-0.228278626	0.0251057	-0.098733379
T2_20864	T2	Normal	-0.2219273	0.029337529	0.2090063	-0.024811994
T2_20865	T2	Normal	-0.2186164	-0.007849997	0.3931242	-0.183599167
T2_20866	T2	Normal	0.39402496	0.45883245	0.1618261	-0.156800167
T2_20867	T2	Normal	0.18056888	0.01007691	-0.191618	-0.040217187
T2_20868	T2	Normal	0.33598561	-0.234839988	0.3378091	0.646716404
T2_20869	T2	Normal	0.23972385	0.095390129	-0.072818	-0.023704598
T2_20870	T2	Normal	-0.2012612	0.01020885	-0.236308	-0.001136881
T2_20871	T2	Normal	-0.0778178	0.059591126	-0.174551	0.248217353
T2_20872	T2	Normal	-0.2366609	0.001274074	-0.15807	-0.015834927
T2_20873	T2	Normal	-0.2413645	0.013912813	-0.169758	0.035254825
T2_20874	T2	Normal	-0.2712156	0.029560837	-0.304382	0.090458894
T2_20875	T2	Normal	-0.292698	0.082630607	0.2645823	-0.178493294
T2_20876	T2	Model	0.22489264	-0.19839827	-0.268541	-0.005965384
T2_20877	T2	Model	-0.249699	0.015584012	-0.330682	0.090351068
T2_20878	T2	Model	0.18493481	-0.181580303	-0.058723	-0.056915638
T2_20879	T2	Model	-0.2181478	-0.007430016	0.1147262	-0.168554354
T2_20880	T2	Model	0.21085535	0.284350795	-0.015462	-0.122306713
T2_20881	T2	Model	-0.1190922	0.018595235	0.2006156	-0.212857316
T2_20882	T2	Model	0.2914314	-0.212671591	0.2640401	-0.200160675
T2_20884	T2	Model	-0.1048917	0.021764878	0.0274506	-0.130355777
T2_20885	T2	Model	-0.0889943	-0.017385156	0.2491717	-0.0209112
T2_20887	T2	Model	-0.0969491	-0.077615345	-0.257641	0.030396211
T2_20888	T2	Model	0.08875958	-0.118881115	0.1919857	-0.172627294
T2_20895	T2	Model	-0.2549322	0.051998446	0.0542757	-0.167442214
T2_20896	T2	Model	0.35449225	-0.249189129	-0.311797	0.168975697
T2_20897	T2	Model	-0.1823541	0.004671744	-0.28526	0.094236633
T2_20898	T2	Model	-0.0876094	-0.07957017	-0.232666	0.165894632
T2_20899	T2	Model	0.27836571	-0.22807847	-0.228742	-0.012684848
T3_20800	T2	Model	0.11563039	-0.179633233	-0.11695	-0.077573574
T3_20801	T2	Model	0.14314951	-0.162018645	0.3170314	0.404253151
T3_20802	T4	Normal	0.21271066	-0.193227558	0.3798386	0.713408082
T3_20803	T2	Model	-0.2603691	0.029721763	-0.284362	0.014125398
T3_20804	T4	Model	0.25786543	-0.211251038	0.0008942	-0.08579992
T3_20806	T4	Model	-0.1909312	-0.005948479	0.2468668	-0.115800107
T3_20808	T2	Model	0.03543139	-0.139298741	0.027317	-0.092618947
T3_20809	T4	Model	-0.2728167	0.047569778	0.4394199	-0.196857497
T3_20810	T4	Normal	0.38951339	-0.221025501	0.0847985	0.224826551
T3_20811	T4	Model	-0.2827603	0.041091237	0.0948376	0.203547125
T3_20812	T4	Model	-0.0991047	-0.031719506	0.2925698	-0.207101541
T3_20813	T4	Model	-0.0963251	-0.0918379	-0.13896	-0.030138814
T3_20814	T4	Model	-0.2476082	-0.002812231	-0.128513	0.150502371
T3_20815	T4	Model	0.26256305	-0.186503065	0.3071005	0.284144742
T3_20816	T4	Model	0.35053711	-0.199267399	-0.26075	0.020715602
T3_20817	T4	Model	0.04039038	-0.120595109	0.3596785	-0.171191055
T3_20818	T4	Model	0.1245039	-0.010857632	-0.25917	0.056149441
T3_20819	T4	Model	0.05844954	-0.144979731	-0.135064	0.165751715
T3_20820	T4	Model	0.38772273	-0.23821526	0.3791883	0.720854197
T3_20832	T2	Model	0.39802522	0.502429279	-0.159891	-0.008095198
T3_20842	T2	Model	0.42177557	-0.25186357	0.366707	-0.242054286
T3_20843	T2	Model	0.01801152	-0.111841669	0.3005957	-0.181676191
T3_20845	T2	Model	-0.1016982	-0.080435876	0.416001	-0.241669972
T3_20846	T2	Model	0.35089221	0.410542893	-0.216724	-0.009827706
T3_20847	T2	Model	0.29188984	-0.226799198	-0.19999	-0.038845408
T3_20849	T2	Model	0.31934101	0.264283492	0.197421	-0.206076477
T3_20850	T2	Model	-0.1076252	0.166117867	-0.051579	0.088115444
T3_20851	T2	Model	0.40186841	0.719325948	0.3168067	-0.21826395
T3_20853	T2	Model	0.11830994	-0.117448301	-0.226304	0.178660872
T3_20854	T2	Model	0.32023886	-0.226694341	0.1443548	0.178714589
T3_20855	T2	Model	-0.3013388	0.067657105	-0.17788	-0.017286611
T3_20857	T2	Model	0.33114583	0.310070142	-0.239353	-0.000749016
T3_20859	T2	Model	-0.0667871	0.327384972	0.2332309	-0.201563906
T3_20860	T2	Model	-0.1615367	-0.036162489	0.4340069	0.402594287
T3_20861	T2	Model	-0.201486	-0.027632732	-0.187975	-0.016873244
T3_20862	T2	Model	-0.0208416	-0.105643685	0.0701447	-0.035948065
T3_20863	T2	Model	0.21442721	-0.216577737	-0.25618	0.194232284
T3_20864	T2	Model	-0.1626452	-0.042772746	0.3414937	0.503576896
T3_20865	T2	Model	0.39248218	-0.249838895	0.1494622	0.037006906
T3_20866	T2	Model	-0.1774452	-0.01371826	0.1860029	0.046230577
T3_20867	T2	Model	-0.1393326	0.148292778	0.1313212	0.273606405
T3_20868	T2	Model	-0.2483165	0.052172437	-0.132471	0.013625467
T3_20869	T2	Model	0.22955469	0.304493061	0.3570335	-0.18093184
T3_20870	T2	Model	-0.1666738	0.019085425	0.0604722	-0.16997525
T3_20871	T2	Model	0.0171288	-0.074380694	-0.256486	0.021755485
T3_20872	T3	Model	-0.2440009	0.006420797	-0.076492	-0.092867093
T3_20873	T3	Model	-0.1712568	-0.024523226	0.3197581	-0.217447573
T3_20874	T3	Normal	-0.230947	0.020924331	-0.261704	0.039158457
T3_20875	T3	Model	-0.1242099	-0.029904086	-0.250458	0.050112998
T3_20876	T3	Normal	-0.1672426	-0.041937827	-0.02006	-0.068136966
T3_20877	T3	Normal	-0.2373391	0.013017831	-0.117867	0.028122939
T3_20878	T3	Model	-0.2247826	0.014684436	-0.237737	0.001368118
T3_20879	T3	Normal	-0.0787282	-0.055582645	0.0726314	-0.01747598
T3_20880	T3	Normal	0.27381256	-0.118043927	-0.228357	0.028234627
T3_20881	T3	Normal	0.46089882	-0.218021032	-0.027704	-0.073662412
T3_20882	T3	Normal	0.13038287	0.183658837	0.3888941	0.568061659
T3_20884	T3	Normal	-0.1358301	-0.026227922	-0.047549	-5.57E-05
T3_20885	T3	Normal	-0.129547	0.15749285	-0.225192	0.041754088
T3_20887	T3	Normal	-0.2528107	0.02653034	0.1396651	-0.142518534
T3_20888	T3	Normal	0.37500209	-0.186307488	0.1038973	0.321262512
T3_20895	T3	Normal	-0.2524817	0.064153397	0.3554071	0.248223942
T3_20896	T3	Normal	-0.236004	0.028347287	0.2268344	0.338285642
T3_20897	T3	Normal	-0.3229032	0.064259266	0.1973954	-0.166497301
T3_20898	T3	Normal	-0.1075023	-0.044214229	-0.115044	-0.031986345
T3_20899	T3	Model	-0.2421632	0.03731689	0.3735173	0.461530885
T4_20800	T3	Model	-0.1619524	-0.044484659	0.1415296	0.025584419
T4_20801	T3	Model	0.00766614	-0.121197481	0.3252458	-0.227337921
T4_20802	T4	Model	-0.2357772	0.138628658	0.1018733	-0.111395087
T4_20803	T3	Model	-0.2926741	0.051612795	0.1976141	0.093293604
T4_20804	T4	Model	0.21715901	-0.159954774	-0.248972	0.023424398
T4_20806	T4	Model	0.12383045	-0.172785187	-0.337759	0.072809677
T4_20808	T3	Model	-0.29216	0.060793132	-0.221184	0.01480096
T4_20809	T4	Model	0.30003392	-0.198843187	-0.081118	-0.068983695
T4_20810	T4	Model	-0.1769816	0.02861396	0.3076163	-0.214287811
T4_20811	T4	Model	-0.0319055	-0.111028005	-0.244092	0.025328818
T4_20812	T4	Model	0.24136504	-0.055416815	-0.32139	0.089157467
T4_20813	T4	Model	-0.2755437	0.049925723	-0.181243	-0.038689769
T4_20814	T4	Model	0.41085863	-0.200143783	0.0746386	-0.141891055
T4_20815	T4	Model	-0.1674656	-0.044461862	0.3188627	0.317755523
T4_20816	T4	Model	-0.2220168	0.011305908	-0.211426	0.115374765
T4_20817	T4	Model	-0.2732071	0.063438292	-0.301965	0.060656101
T4_20818	T4	Model	0.27876794	-0.177878259	0.2099527	-0.149912758
T4_20819	T4	Model	-0.2535352	0.051342648	-0.060491	0.34455153
T4_20820	T4	Model	-0.1995329	0.009154334	-0.188327	-0.027156237
T4_20832	T3	Model	0.40409657	0.551347652	-0.06957	0.057854973
T4_20842	T3	Model	0.01175003	-0.135065519	-0.242696	-0.007467892
T4_20843	T3	Model	0.13712172	-0.174917963	-0.2456	0.018628266
T4_20845	T3	Model	-0.2132072	-0.003800283	-0.28177	0.031605634
T4_20846	T3	Model	0.4686342	0.342233459	-0.294999	0.070253332
T4_20847	T3	Model	-0.2627458	0.070240593	0.2183073	-0.189717061
T4_20849	T3	Model	0.39812002	0.544156447	-0.242122	-0.004859694
T4_20850	T3	Model	0.36071924	0.084483914	0.1699329	-0.177332944
T4_20851	T3	Model	0.40283603	0.713610784	-0.220314	-0.022109726
T4_20853	T3	Model	0.11722959	0.200168527	0.1774444	0.314182098
T4_20854	T3	Model	0.41659721	0.181956827	-0.080134	-0.004377803
T4_20855	T3	Model	0.46858687	0.264440279	0.289741	-0.21147394
T4_20857	T3	Model	0.28858387	0.005365928	-0.136772	0.033154306
T4_20859	T3	Model	0.42422341	0.269683307	0.0245047	-0.05494175
T4_20860	T3	Model	-0.1541794	-0.065077321	-0.080853	-0.089955826
T4_20861	T3	Model	-0.2445281	0.037920759	0.0923326	-0.118186143
T4_20862	T3	Model	0.00707365	-0.108321686	-0.259034	0.036852268
T4_20863	T3	Model	0.02182976	-0.156763264	0.343166	-0.234027702
T4_20864	T3	Model	0.2339837	-0.19910297	-0.185283	-0.012374856
T4_20865	T3	Model	-0.159224	-0.045094964	-0.101632	-0.078332883
T4_20866	T3	Model	-0.1561735	0.053407391	0.2723992	-0.222198706
T4_20867	T3	Model	0.1716924	-0.01777151	0.1207234	-0.171371751
T4_20868	T3	Model	0.1421369	-0.163745131	0.1157212	-0.151434604
T4_20869	T3	Model	0.36809197	0.642365527	0.262215	-0.199289394
T4_20870	T3	Model	-0.1133619	-0.012887777	-0.263615	0.018472271
T4_20871	T3	Model	-0.1603309	0.22741145	0.2287688	-0.193755758
T4_20872	T3	Model	-0.1779938	-0.003246951	-0.205761	-0.018062596
T4_20873	T3	Model	-0.1701796	0.027047033	0.0156242	-0.136264622
T4_20874	T3	Model	-0.0208165	-0.071615651	-0.272546	0.024038431
T4_20875	T3	Model	-0.0775111	0.015660121	0.3729966	-0.216080199
T4_20876	T3	Model	0.08710552	-0.027831329	-0.291085	0.02254956
T4_20877	T3	Model	-0.0446364	-0.064260235	-0.082686	-0.046128323
T4_20878	T3	Model	-0.0535702	-0.006197247	-0.076274	-0.110316127
T4_20879	T3	Model	-0.2163617	0.048789612	-0.253485	-0.020863163
T4_20880	T3	Model	0.14432463	0.295493616	0.2796129	-0.194211468
T4_20881	T3	Model	0.33123699	0.231024092	0.3316495	-0.194803321
T4_20882	T3	Model	0.24141538	0.325034845	0.1158249	-0.149595885
T4_20884	T3	Model	0.18200884	-0.1835357	0.1078161	-0.009106935
T4_20885	T3	Model	0.2320325	0.12969574	0.0267567	-0.134929119
T4_20887	T3	Model	-0.1183464	0.0552729	0.3886087	-0.241341562
T4_20888	T3	Model	0.41707967	-0.031606353	0.3829717	0.515752716
T4_20895	T4	Model	0.01971004	-0.090188345	0.4109832	-0.23545439
T4_20896	T4	Model	-0.2662265	0.042754635	-0.011302	-0.09743936
T4_20897	T4	Normal	-0.2348724	0.170076557	0.4467573	0.365849384
T4_20898	T4	Model	-0.1397972	0.070663674	-0.129335	-0.062131208
T4_20899	T4	Normal	-0.202347	0.001467908	0.2116891	-0.185857626

Supplementary Table 5 | The taxonomic of the gut microbiota and Kruskal-Wallis(KW) test for the gut microbiota species in OVX group mice at different time point .

Metaphlan Species	kw.p	p_T2_T1	p_T3_T1	p_T4_T1	p_T3_T2	p_T4_T2	p_T4_T3	Nearby
s__Veillonella_unclassified	2.19E-25	8.54E-09	1.61E-17	9.12E-23	0.006695	5.52E-05	0.17533	T1 < T2 < T3 <= T4
s__Escherichia_coli	3.26E-10	6.84E-06	3.68E-07	1.32E-09	0.518518	0.132945	0.348593	T1 < T2 <= T3 <= T4
s__Firmicutes_bacterium_M10_2	1.03E-09	6.20E-05	0.008934	4.48E-10	0.13174	0.029078	0.000339	T1 < T3 <= T2 < T4
s__Dysgonomonas_unclassified	1.16E-06	0.413411	0.027991	1.92E-06	0.149937	5.28E-05	0.011595	T1 <= T2 <= T3 < T4
s__Parabacteroides_unclassified	2.63E-05	0.589847	0.260049	4.82E-05	0.493013	0.00026	0.003831	T1 <= T2 <= T3 < T4
s__Parabacteroides_distasonis	3.62E-05	0.146545	0.0445	1.38E-05	0.529627	0.004431	0.021506	T1 <= T2 <= T3 < T4
s__Paraprevotella_unclassified	0.0013042	0.156616	0.076722	0.00054	0.576233	0.048648	0.09753	T1 <= T2 <= T3 <= T4
s__Dorea_unclassified	0.0016491	0.473784	0.177159	0.001198	0.473784	0.012198	0.062065	T1 <= T2 <= T3 <= T4
s__Peptostreptococcaceae_noname_unclassified	0.0182155	0.323328	0.245238	0.010691	0.772528	0.130141	0.166918	T1 <= T2 <= T3 <= T4
s__Lachnospiraceae_bacterium_8_1_57FAA	0.0276123	0.158676	0.029018	0.029018	0.485963	0.485963	0.953844	T1 <= T2 <= T3 <= T4
s__Bacteroides_xylanisolvens	0.0332314	0.41938	0.222778	0.02469	0.511574	0.159481	0.302283	T1 <= T2 <= T3 <= T4
s__Desulfovibrio_termitidis	0.0916854	0.6792169	0.5289038	0.0954847	0.6792169	0.1743336	0.2770393	T1 <= T2 <= T3 <= T4
s__Bacteroides_uniformis	0.1150932	0.7769077	0.2076114	0.2076114	0.2687497	0.2076114	0.7769077	T1 <= T2 <= T3 <= T4
s__Acinetobacter_unclassified	0.2787332	0.6275095	0.6275095	0.3366888	0.9949028	0.4118067	0.4118067	T1 <= T2 <= T3 <= T4
s__Eubacterium_cellulosolvens	0.2824347	0.6275095	0.6275095	0.341653	0.9898058	0.4210422	0.4210422	T1 <= T2 <= T3 <= T4
s__Lachnospiraceae_bacterium_9_1_43BFAA	0.3540276	0.9959396	0.730853	0.3564501	0.730853	0.3564501	0.5889812	T1 <= T2 <= T3 <= T4
s__Terriglobus_unclassified	0.5333214	0.7498168	0.7240697	0.7240697	0.7498168	0.7240697	0.7240697	T1 <= T2 <= T3 <= T4
s__Lactobacillus_vaginalis	0.548524	0.6426238	0.6426238	0.6426238	0.9820798	0.8507924	0.8507924	T1 <= T2 <= T3 <= T4
s__Eggerthella_unclassified	0.5702499	0.5847364	0.5847364	0.5847364	0.9886313	0.5847364	0.5847364	T1 <= T2 <= T3 <= T4
s__Chlamydophila_unclassified	0.8007934	0.849712	0.849712	0.849712	0.849712	0.849712	0.849712	T1 <= T2 <= T3 <= T4
s__Veillonella_atypica	0.1045712	0.7306346	0.1862733	0.2399199	0.2098627	0.3390385	0.656971	T1 <= T2 <= T4 <= T3
s__Ureaplasma_unclassified	0.5576755	0.5793905	0.5793905	0.5793905	0.5793905	0.9943155	0.5793905	T1 <= T2 <= T4 <= T3
s__Rhodospirillum_unclassified	0.7284928	0.7813955	0.7813955	0.7813955	0.7813955	0.7813955	0.9769759	T1 <= T2 <= T4 <= T3
s__Burkholderiales_bacterium_1_1_47	0.0045298	0.481367	0.586755	0.00417	0.763699	0.032883	0.020827	T1 <= T3 <= T2 < T4
s__Bacteroides_vulgatus	0.2908171	0.8438741	0.9490289	0.3059563	0.8438741	0.4194388	0.3059563	T1 <= T3 <= T2 <= T4
s__Riemerella_unclassified	0.6100227	0.7454362	0.7454362	0.7454362	0.9930217	0.7454362	0.7454362	T1 <= T3 <= T2 <= T4
s__Candidatus_Pelagibacter_ubique	0.6387813	0.7138358	0.9913406	0.7138358	0.7138358	0.7138358	0.7138358	T1 <= T3 <= T2 <= T4
s__Eubacterium_ventriosum	0.8878455	0.9923542	0.9923542	0.9923542	0.9923542	0.9923542	0.9923542	T1 <= T3 <= T2 <= T4
s__Staphylococcus_xylosus	0.0003408	0.000184	0.263748	0.219925	0.009801	0.013178	0.82237	T1 <= T3 <= T4 < T2
s__Pantoea_unclassified	0.0370532	0.025047	0.260248	0.201296	0.260248	0.361556	0.638381	T1 <= T3 <= T4 <= T2
s__Anaerotruncus_sp_G3_2012	0.075335	0.1129981	0.5770338	0.1129981	0.2543708	0.9037372	0.2543708	T1 <= T3 <= T4 <= T2
s__Yersinia_unclassified	0.5263554	0.6108667	0.6375252	0.6108667	0.6375252	0.9949028	0.6375252	T1 <= T3 <= T4 <= T2
s__Lactobacillus_iners	0.0191685	0.522394	0.020699	0.640802	0.093942	0.640802	0.04192	T1 <= T4 <= T2 <= T3
s__Staphylococcus_lentus	1.67E-08	3.63E-07	0.723051	0.739257	4.86E-06	1.11E-06	0.739257	T1 <= T4 <= T3 < T2
s__Escherichia_unclassified	0.0049934	0.020853	0.044021	0.814043	0.632846	0.021629	0.059822	T1 <= T4 <= T3 <= T2
s__Bacteroides_coprocola	0.0728193	0.0696159	0.2297814	0.4521032	0.4521032	0.2645883	0.4521032	T1 <= T4 <= T3 <= T2
s__Bacteroides_intestinalis	0.5476748	0.7457888	0.7457888	0.8894846	0.7457888	0.7457888	0.7457888	T1 <= T4 <= T3 <= T2
s__Shigella_sonnei	0.6449159	0.8432309	0.8432309	0.8681233	0.9414467	0.9210033	0.9210033	T1 <= T4 <= T3 <= T2
s__Wolbachia_pipientis	0.5703026	1	0.4777851	0.4777851	0.4777851	0.4777851	1	T1 <=> T2 <= T3 <=> T4
s__Desulfobulbus_sp_oral_taxon_041	0.5703026	1	0.4777851	0.4777851	0.4777851	0.4777851	1	T1 <=> T2 <= T4 <=> T3
s__Mycoplasma_capricolum	0.5703026	1	0.4777851	0.4777851	0.4777851	0.4777851	1	T1 <=> T2 <= T4 <=> T3
s__Pseudomonas_aeruginosa	0.0067846	1	1	0.008749	1	0.008749	0.008749	T1 <=> T2 <=> T3 < T4
s__Borrelia_recurrentis	0.0281083	1	1	0.027648	1	0.027648	0.027648	T1 <=> T2 <=> T3 < T4
s__Proteus_penneri	0.1101525	1	1	0.0899221	1	0.0899221	0.0899221	T1 <=> T2 <=> T3 <= T4
s__Acetobacter_unclassified	0.3916252	1	1	0.3145984	1	0.3145984	0.3145984	T1 <=> T2 <=> T3 <= T4
s__Alistipes_shahii	0.3916252	1	1	0.3145984	1	0.3145984	0.3145984	T1 <=> T2 <=> T3 <= T4
s__Bacillus_cereus_thuringiensis	0.3916252	1	1	0.3145984	1	0.3145984	0.3145984	T1 <=> T2 <=> T3 <= T4
s__Butyrivibrio_fibrisolvens	0.3916252	1	1	0.3145984	1	0.3145984	0.3145984	T1 <=> T2 <=> T3 <= T4
s__Candidatus_Arthromitus_sp_SFB_5	0.3916252	1	1	0.3145984	1	0.3145984	0.3145984	T1 <=> T2 <=> T3 <= T4
s__Candidatus_Moranella_endobia	0.3916252	1	1	0.3145984	1	0.3145984	0.3145984	T1 <=> T2 <=> T3 <= T4
s__Escherichia_albertii	0.3916252	1	1	0.3145984	1	0.3145984	0.3145984	T1 <=> T2 <=> T3 <= T4
s__Johnsonella_ignava	0.3916252	1	1	0.3145984	1	0.3145984	0.3145984	T1 <=> T2 <=> T3 <= T4
s__Lachnospiraceae_bacterium_2_1_58FAA	0.3916252	1	1	0.3145984	1	0.3145984	0.3145984	T1 <=> T2 <=> T3 <= T4
s__Leifsonia_unclassified	0.3916252	1	1	0.3145984	1	0.3145984	0.3145984	T1 <=> T2 <=> T3 <= T4
s__Neisseria_subflava	0.3916252	1	1	0.3145984	1	0.3145984	0.3145984	T1 <=> T2 <=> T3 <= T4
s__Thiothrix_unclassified	0.3916252	1	1	0.3145984	1	0.3145984	0.3145984	T1 <=> T2 <=> T3 <= T4
s__Wolbachia_unclassified	0.3916252	1	1	0.3145984	1	0.3145984	0.3145984	T1 <=> T2 <=> T3 <= T4
s__Alloprevotella_rava	0.1101525	1	0.0899221	1	0.0899221	1	0.0899221	T1 <=> T2 <=> T4 <= T3
s__Alloprevotella_tannerae	0.1101525	1	0.0899221	1	0.0899221	1	0.0899221	T1 <=> T2 <=> T4 <= T3
s__Lactobacillus_sp_7_1_47FAA	0.1101525	1	0.0899221	1	0.0899221	1	0.0899221	T1 <=> T2 <=> T4 <= T3
s__Porphyromonas_endodontalis	0.1101525	1	0.0899221	1	0.0899221	1	0.0899221	T1 <=> T2 <=> T4 <= T3
s__Porphyromonas_gingivalis	0.1101525	1	0.0899221	1	0.0899221	1	0.0899221	T1 <=> T2 <=> T4 <= T3
s__Prevotella_intermedia	0.1101525	1	0.0899221	1	0.0899221	1	0.0899221	T1 <=> T2 <=> T4 <= T3
s__Prevotella_pleuritidis	0.1101525	1	0.0899221	1	0.0899221	1	0.0899221	T1 <=> T2 <=> T4 <= T3
s__Ureaplasma_parvum	0.1101525	1	0.0899221	1	0.0899221	1	0.0899221	T1 <=> T2 <=> T4 <= T3
s__Actinoplanes_unclassified	0.3916252	1	0.3145984	1	0.3145984	1	0.3145984	T1 <=> T2 <=> T4 <= T3
s__Asticcacaulis_unclassified	0.3916252	1	0.3145984	1	0.3145984	1	0.3145984	T1 <=> T2 <=> T4 <= T3
s__Bacteroides_salyersiae	0.3916252	1	0.3145984	1	0.3145984	1	0.3145984	T1 <=> T2 <=> T4 <= T3
s__Bacteroidetes_bacterium_oral_taxon_272	0.3916252	1	0.3145984	1	0.3145984	1	0.3145984	T1 <=> T2 <=> T4 <= T3
s__Bacteroidetes_oral_taxon_274	0.3916252	1	0.3145984	1	0.3145984	1	0.3145984	T1 <=> T2 <=> T4 <= T3
s__Bilophila_wadsworthia	0.3916252	1	0.3145984	1	0.3145984	1	0.3145984	T1 <=> T2 <=> T4 <= T3
s__Borrelia_duttonii	0.3916252	1	0.3145984	1	0.3145984	1	0.3145984	T1 <=> T2 <=> T4 <= T3
s__Bulleidia_extructa	0.3916252	1	0.3145984	1	0.3145984	1	0.3145984	T1 <=> T2 <=> T4 <= T3
s__Campylobacter_rectus	0.3916252	1	0.3145984	1	0.3145984	1	0.3145984	T1 <=> T2 <=> T4 <= T3
s__Campylobacter_showae	0.3916252	1	0.3145984	1	0.3145984	1	0.3145984	T1 <=> T2 <=> T4 <= T3
s__Crenarchaeota_archaeon_SCGC_AAA471_O08	0.3916252	1	0.3145984	1	0.3145984	1	0.3145984	T1 <=> T2 <=> T4 <= T3
s__Eubacterium_saphenum	0.3916252	1	0.3145984	1	0.3145984	1	0.3145984	T1 <=> T2 <=> T4 <= T3
s__Facklamia_unclassified	0.3916252	1	0.3145984	1	0.3145984	1	0.3145984	T1 <=> T2 <=> T4 <= T3
s__Filifactor_alocis	0.3916252	1	0.3145984	1	0.3145984	1	0.3145984	T1 <=> T2 <=> T4 <= T3
s__Fretibacterium_fastidiosum	0.3916252	1	0.3145984	1	0.3145984	1	0.3145984	T1 <=> T2 <=> T4 <= T3
s__Fusobacterium_nucleatum	0.3916252	1	0.3145984	1	0.3145984	1	0.3145984	T1 <=> T2 <=> T4 <= T3
s__Lactobacillus_delbrueckii	0.3916252	1	0.3145984	1	0.3145984	1	0.3145984	T1 <=> T2 <=> T4 <= T3
s__Lactobacillus_sp_ASF360	0.3916252	1	0.3145984	1	0.3145984	1	0.3145984	T1 <=> T2 <=> T4 <= T3
s__Lysinibacillus_sphaericus	0.3916252	1	0.3145984	1	0.3145984	1	0.3145984	T1 <=> T2 <=> T4 <= T3
s__Micrococcus_luteus	0.3916252	1	0.3145984	1	0.3145984	1	0.3145984	T1 <=> T2 <=> T4 <= T3
s__Peptostreptococcus_stomatis	0.3916252	1	0.3145984	1	0.3145984	1	0.3145984	T1 <=> T2 <=> T4 <= T3
s__Porphyromonas_asaccharolytica	0.3916252	1	0.3145984	1	0.3145984	1	0.3145984	T1 <=> T2 <=> T4 <= T3
s__Prevotella_disiens	0.3916252	1	0.3145984	1	0.3145984	1	0.3145984	T1 <=> T2 <=> T4 <= T3
s__Prevotella_stercorea	0.3916252	1	0.3145984	1	0.3145984	1	0.3145984	T1 <=> T2 <=> T4 <= T3
s__Propionibacterium_acnes	0.3916252	1	0.3145984	1	0.3145984	1	0.3145984	T1 <=> T2 <=> T4 <= T3
s__Rothia_mucilaginosa	0.3916252	1	0.3145984	1	0.3145984	1	0.3145984	T1 <=> T2 <=> T4 <= T3
s__Tannerella_forsythia	0.3916252	1	0.3145984	1	0.3145984	1	0.3145984	T1 <=> T2 <=> T4 <= T3
s__Thermus_scotoductus	0.3916252	1	0.3145984	1	0.3145984	1	0.3145984	T1 <=> T2 <=> T4 <= T3
s__Treponema_denticola	0.3916252	1	0.3145984	1	0.3145984	1	0.3145984	T1 <=> T2 <=> T4 <= T3
s__Treponema_lecithinolyticum	0.3916252	1	0.3145984	1	0.3145984	1	0.3145984	T1 <=> T2 <=> T4 <= T3
s__Treponema_maltophilum	0.3916252	1	0.3145984	1	0.3145984	1	0.3145984	T1 <=> T2 <=> T4 <= T3
s__Treponema_medium	0.3916252	1	0.3145984	1	0.3145984	1	0.3145984	T1 <=> T2 <=> T4 <= T3
s__Treponema_socranskii	0.3916252	1	0.3145984	1	0.3145984	1	0.3145984	T1 <=> T2 <=> T4 <= T3
s__Treponema_vincentii	0.3916252	1	0.3145984	1	0.3145984	1	0.3145984	T1 <=> T2 <=> T4 <= T3
s__Lachnospiraceae_bacterium_2_1_46FAA	0.1370842	0.3054334	1	0.1634703	0.3054334	0.6175751	0.1634703	T1 <=> T3 <= T2 <= T4
s__Clostridiales_bacterium_1_7_47FAA	0.5703026	0.4777851	1	0.4777851	0.4777851	1	0.4777851	T1 <=> T3 <= T2 <=> T4
s__Lachnobacterium_bovis	0.5703026	0.4777851	1	0.4777851	0.4777851	1	0.4777851	T1 <=> T3 <= T2 <=> T4
s__Bacteroides_helcogenes	0.5703026	0.4777851	1	0.4777851	0.4777851	1	0.4777851	T1 <=> T3 <= T4 <=> T2
s__Megamonas_unclassified	0.0003538	0.000903	1	1	0.000903	0.000903	1	T1 <=> T3 <=> T4 < T2
s__Prevotella_bivia	0.0281083	0.027648	1	1	0.027648	0.027648	1	T1 <=> T3 <=> T4 < T2
s__Bacteroides_pectinophilus	0.1101525	0.0899221	1	1	0.0899221	0.0899221	1	T1 <=> T3 <=> T4 <= T2
s__Lachnospiraceae_bacterium_5_1_63FAA	0.1101525	0.0899221	1	1	0.0899221	0.0899221	1	T1 <=> T3 <=> T4 <= T2
s__Alistipes_onderdonkii	0.3916252	0.3145984	1	1	0.3145984	0.3145984	1	T1 <=> T3 <=> T4 <= T2
s__Bacteroides_salanitronis	0.3916252	0.3145984	1	1	0.3145984	0.3145984	1	T1 <=> T3 <=> T4 <= T2
s__Burkholderia_unclassified	0.3916252	0.3145984	1	1	0.3145984	0.3145984	1	T1 <=> T3 <=> T4 <= T2
s__Clostridium_phytofermentans	0.3916252	0.3145984	1	1	0.3145984	0.3145984	1	T1 <=> T3 <=> T4 <= T2
s__Corynebacterium_crenatum	0.3916252	0.3145984	1	1	0.3145984	0.3145984	1	T1 <=> T3 <=> T4 <= T2
s__Cyanobium_unclassified	0.3916252	0.3145984	1	1	0.3145984	0.3145984	1	T1 <=> T3 <=> T4 <= T2
s__Eubacterium_ramulus	0.3916252	0.3145984	1	1	0.3145984	0.3145984	1	T1 <=> T3 <=> T4 <= T2
s__Haemophilus_haemolyticus	0.3916252	0.3145984	1	1	0.3145984	0.3145984	1	T1 <=> T3 <=> T4 <= T2
s__Haemophilus_influenzae	0.3916252	0.3145984	1	1	0.3145984	0.3145984	1	T1 <=> T3 <=> T4 <= T2
s__Lachnospiraceae_bacterium_6_1_63FAA	0.3916252	0.3145984	1	1	0.3145984	0.3145984	1	T1 <=> T3 <=> T4 <= T2
s__Lactobacillus_amylovorus	0.3916252	0.3145984	1	1	0.3145984	0.3145984	1	T1 <=> T3 <=> T4 <= T2
s__Mobiluncus_unclassified	0.3916252	0.3145984	1	1	0.3145984	0.3145984	1	T1 <=> T3 <=> T4 <= T2
s__Nitrosospira_unclassified	0.3916252	0.3145984	1	1	0.3145984	0.3145984	1	T1 <=> T3 <=> T4 <= T2
s__Pantoea_dispersa	0.3916252	0.3145984	1	1	0.3145984	0.3145984	1	T1 <=> T3 <=> T4 <= T2
s__Rhodopseudomonas_palustris	0.3916252	0.3145984	1	1	0.3145984	0.3145984	1	T1 <=> T3 <=> T4 <= T2
s__Shewanella_unclassified	0.3916252	0.3145984	1	1	0.3145984	0.3145984	1	T1 <=> T3 <=> T4 <= T2
s__Rickettsia_japonica	0.5703026	0.4777851	0.4777851	1	1	0.4777851	0.4777851	T1 <=> T4 <= T3 <=> T2
s__Neisseria_unclassified	9.11E-06	0.010335	0.659929	0.037013	0.019436	2.22E-06	0.018694	T2 < T3 <= T1 < T4
s__Bacteroides_dorei	1.77E-07	0.35666	0.145932	1.78E-05	0.020278	3.01E-07	0.005742	T2 <= T1 <= T3 < T4
s__Olsenella_unclassified	1.81E-05	0.163955	0.88188	0.002603	0.152431	8.67E-06	0.002929	T2 <= T1 <= T3 < T4
s__Alloprevotella_unclassified	2.23E-05	0.953843	0.461757	8.03E-05	0.461757	8.03E-05	0.001735	T2 <= T1 <= T3 < T4
s__Lactobacillus_hominis	7.41E-05	0.938026	0.785419	0.000287	0.785419	0.000287	0.001105	T2 <= T1 <= T3 < T4
s__Chlorobium_phaeobacteroides	0.0003012	0.524901	0.521807	0.002072	0.234836	0.000336	0.017985	T2 <= T1 <= T3 < T4
s__Sphingobacterium_unclassified	0.0131466	0.783877	0.355318	0.022029	0.2807	0.018751	0.203662	T2 <= T1 <= T3 <= T4
s__Ruminococcus_flavefaciens	0.0246703	0.370571	0.843844	0.140172	0.33672	0.015856	0.146325	T2 <= T1 <= T3 <= T4
s__Pedobacter_unclassified	0.0409319	0.209225	0.922111	0.309906	0.209225	0.026133	0.309906	T2 <= T1 <= T3 <= T4
s__Buchnera_aphidicola	0.0662683	0.9963498	0.7591195	0.0645389	0.7591195	0.0645389	0.1372783	T2 <= T1 <= T3 <= T4
s__Butyrivibrio_unclassified	0.0962854	0.4808621	0.4808621	0.2683165	0.3141273	0.0961185	0.480737	T2 <= T1 <= T3 <= T4
s__Ruminococcus_torques	0.1341346	0.6030296	0.6030296	0.2558473	0.4378797	0.1449143	0.4378797	T2 <= T1 <= T3 <= T4
s__Klebsiella_unclassified	0.1401313	0.9824196	0.3881447	0.1541353	0.3881447	0.1541353	0.4953532	T2 <= T1 <= T3 <= T4
s__Veillonella_parvula	0.3675077	0.9756415	0.7480417	0.3824234	0.7480417	0.3824234	0.6031568	T2 <= T1 <= T3 <= T4
s__Megasphaera_unclassified	0.4974933	0.8714851	0.9856743	0.5564456	0.8714851	0.5564456	0.5564456	T2 <= T1 <= T3 <= T4
s__Providencia_unclassified	0.5702499	0.5847364	0.9886313	0.5847364	0.5847364	0.5847364	0.5847364	T2 <= T1 <= T3 <= T4
s__Cytophaga_unclassified	0.8026764	0.897242	0.9780848	0.9780848	0.897242	0.897242	0.9780848	T2 <= T1 <= T3 <= T4
s__Brucella_microti	0.876844	0.9860061	0.858935	0.858935	0.858935	0.858935	0.9860061	T2 <= T1 <= T3 <= T4
s__Lachnospiraceae_bacterium_1_1_57FAA	0.1503821	0.5448634	0.3761534	0.5448634	0.1587308	0.3761534	0.5448634	T2 <= T1 <= T4 <= T3
s__Actinobacillus_unclassified	0.4515701	0.5292818	0.5292818	0.9552288	0.5292818	0.5292818	0.5292818	T2 <= T1 <= T4 <= T3
s__Clostridium_sp_ASF502	0.9983068	0.9875899	0.9875899	0.9875899	0.9875899	0.9875899	0.9875899	T2 <= T1 <= T4 <= T3
s__Haemophilus_parainfluenzae	0.7847967	0.7437281	0.7437281	1	0.7437281	0.7437281	0.7437281	T2 <= T1 <=> T4 <= T3
s__Alistipes_indistinctus	1.62E-05	0.02976	0.031969	0.081337	0.894288	0.000109	0.000109	T2 <= T3 < T1 <= T4
s__Parabacteroides_johnsonii	3.68E-05	0.012487	0.101872	0.101872	0.311665	4.73E-05	0.001641	T2 <= T3 <= T1 <= T4
s__Parabacteroides_merdae	0.0012484	0.002845	0.127425	0.891424	0.127425	0.002845	0.127425	T2 <= T3 <= T1 <= T4
s__Butyricimonas_synergistica	0.0016526	0.044255	0.421768	0.165113	0.165113	0.000817	0.044255	T2 <= T3 <= T1 <= T4
s__Capnocytophaga_unclassified	0.0024314	0.090452	0.327616	0.157706	0.327616	0.001747	0.024575	T2 <= T3 <= T1 <= T4
s__Collinsella_unclassified	0.0141541	0.178151	0.792638	0.178151	0.186934	0.007115	0.178151	T2 <= T3 <= T1 <= T4
s__Prevotella_melaninogenica	0.0164178	0.369905	0.991871	0.074078	0.369905	0.011059	0.074078	T2 <= T3 <= T1 <= T4
s__Mitsuokella_unclassified	0.0190677	0.557855	0.939403	0.059226	0.557855	0.017177	0.059226	T2 <= T3 <= T1 <= T4
s__Cellulophaga_unclassified	0.0230816	0.094467	0.423824	0.423824	0.276607	0.019027	0.209039	T2 <= T3 <= T1 <= T4
s__Alistipes_senegalensis	0.0661349	0.0715256	0.3865648	0.9302537	0.3865648	0.0715256	0.3865648	T2 <= T3 <= T1 <= T4
s__Bacteroides_massiliensis	0.0685338	0.3323083	0.5599026	0.3323083	0.5599026	0.0691109	0.1558395	T2 <= T3 <= T1 <= T4
s__Anaerotruncus_unclassified	0.0865232	0.1116216	0.5540616	0.8043458	0.3556628	0.1116216	0.4878594	T2 <= T3 <= T1 <= T4
s__Deinococcus_unclassified	0.1024255	0.3039921	0.5462471	0.4884416	0.4884416	0.0950078	0.3039921	T2 <= T3 <= T1 <= T4
s__Dorea_formicigenerans	0.1247556	0.1911766	0.1911766	0.9512911	0.9512911	0.1911766	0.1911766	T2 <= T3 <= T1 <= T4
s__Subdoligranulum_unclassified	0.1365432	0.2440513	0.3417481	0.7335819	0.7335819	0.2440513	0.2440513	T2 <= T3 <= T1 <= T4
s__Ruminococcus_albus	0.1379316	0.2735136	0.7299833	0.6848338	0.3578295	0.1443185	0.542514	T2 <= T3 <= T1 <= T4
s__Oscillibacter_unclassified	0.1543754	0.2116266	0.7071814	0.7538904	0.4091758	0.2030633	0.5902074	T2 <= T3 <= T1 <= T4
s__Clostridium_clostridioforme	0.1839809	0.2174235	0.5739317	0.8521009	0.5540309	0.2174235	0.555767	T2 <= T3 <= T1 <= T4
s__Roseburia_hominis	0.1923025	0.4516011	0.4516011	0.4516011	0.897575	0.2236498	0.2236498	T2 <= T3 <= T1 <= T4
s__Myroides_unclassified	0.2227881	0.4678571	0.5888405	0.5888405	0.5888405	0.2608656	0.4678571	T2 <= T3 <= T1 <= T4
s__Thermosipho_unclassified	0.2352056	0.3429239	0.3429239	0.8970109	0.8848859	0.3429239	0.3429239	T2 <= T3 <= T1 <= T4
s__Brachyspira_unclassified	0.2609509	0.5344257	0.6122204	0.6122204	0.652024	0.3635457	0.4620239	T2 <= T3 <= T1 <= T4
s__Roseburia_intestinalis	0.3672113	0.4959144	0.6672148	0.8070583	0.6367188	0.4959144	0.6367188	T2 <= T3 <= T1 <= T4
s__Thermodesulfobacterium_unclassified	0.4584742	0.5237082	0.5237082	0.9136605	0.9136605	0.5237082	0.5237082	T2 <= T3 <= T1 <= T4
s__Sporolactobacillus_unclassified	0.5386028	0.7658212	0.7658212	0.7658212	0.7658212	0.7658212	0.7658212	T2 <= T3 <= T1 <= T4
s__Neisseria_flavescens	0.5618867	0.5793905	0.9886313	0.5793905	0.5793905	0.5793905	0.5793905	T2 <= T3 <= T1 <= T4
s__Prevotella_nanceiensis	0.5618867	0.5793905	0.9886313	0.5793905	0.5793905	0.5793905	0.5793905	T2 <= T3 <= T1 <= T4
s__Kingella_unclassified	0.5758343	0.5949151	0.5949151	0.9037928	0.9037928	0.5949151	0.5949151	T2 <= T3 <= T1 <= T4
s__Roseburia_unclassified	0.657541	0.7483835	0.7483835	0.8873366	0.7483835	0.7483835	0.7483835	T2 <= T3 <= T1 <= T4
s__Candidatus_Liberibacter_unclassified	0.7858613	0.741815	0.741815	0.9912093	0.9912093	0.741815	0.741815	T2 <= T3 <= T1 <= T4
s__Niabella_unclassified	0.9322908	0.8474554	0.8474554	0.8474554	0.8474554	0.8474554	0.8474554	T2 <= T3 <= T1 <= T4
s__Parabacteroides_goldsteinii	0.0007015	0.001537	0.101108	0.963375	0.119893	0.001537	0.101108	T2 <= T3 <= T4 <= T1
s__Bacteroidales_bacterium_ph8	0.0013352	0.000829	0.018081	0.099842	0.344213	0.09614	0.361937	T2 <= T3 <= T4 <= T1
s__Gillisia_unclassified	0.0239958	0.060778	0.060778	0.633056	0.819378	0.110197	0.136612	T2 <= T3 <= T4 <= T1
s__Helicobacter_canadensis	0.0250085	0.037327	0.037731	0.277242	0.810049	0.277242	0.333149	T2 <= T3 <= T4 <= T1
s__Thermus_unclassified	0.0344159	0.057776	0.057776	0.576922	0.952316	0.152941	0.152941	T2 <= T3 <= T4 <= T1
s__Clostridium_scindens	0.0388712	0.031595	0.175315	0.412629	0.412629	0.175315	0.446469	T2 <= T3 <= T4 <= T1
s__Helicobacter_winghamensis	0.0401128	0.042863	0.093524	0.096607	0.710991	0.710991	0.857035	T2 <= T3 <= T4 <= T1
s__Pseudoflavonifractor_capillosus	0.042964	0.088524	0.088524	0.863214	0.863214	0.088524	0.098803	T2 <= T3 <= T4 <= T1
s__Alicyclobacillus_unclassified	0.0580283	0.0813446	0.1814857	0.5980712	0.5243863	0.1565551	0.3664441	T2 <= T3 <= T4 <= T1
s__Granulicella_unclassified	0.1097985	0.0963724	0.2472507	0.2472507	0.4773858	0.4773858	0.9822481	T2 <= T3 <= T4 <= T1
s__Hymenobacter_unclassified	0.1839664	0.1894847	0.4270109	0.4500631	0.5938822	0.4500631	0.7234813	T2 <= T3 <= T4 <= T1
s__Bacteriovorax_unclassified	0.1912235	0.2948273	0.3881741	0.819851	0.6723372	0.2948273	0.4263577	T2 <= T3 <= T4 <= T1
s__Echinicola_unclassified	0.3866787	0.446345	0.9303773	0.9303773	0.4493263	0.446345	0.9303773	T2 <= T3 <= T4 <= T1
s__Hippea_unclassified	0.4728173	0.6626423	0.7303915	0.8017005	0.7303915	0.6626423	0.7884018	T2 <= T3 <= T4 <= T1
s__Erysipelotrichaceae_bacterium_6_1_45	0.4788727	0.6809952	0.6809952	0.6809952	0.6809952	0.6809952	0.6809952	T2 <= T3 <= T4 <= T1
s__Eubacterium_hallii	0.5293608	0.6246088	0.6526994	0.9796133	0.6526994	0.6246088	0.6526994	T2 <= T3 <= T4 <= T1
s__Peptostreptococcus_unclassified	0.5773583	0.7102051	0.9393442	0.9393442	0.7102051	0.7102051	0.9393442	T2 <= T3 <= T4 <= T1
s__Candidatus_Zinderia_insecticola	0.2383838	0.2717045	0.8942677	0.542932	0.2717045	0.542932	0.542932	T2 <= T4 <= T1 <= T3
s__Crenarchaeota_archaeon_SCGC_AAA471_L14	0.7105048	0.8111937	0.8111937	0.960115	0.8111937	0.8111937	0.8111937	T2 <= T4 <= T1 <= T3
s__Pseudomonas_unclassified	0.8829824	0.9833395	0.9833395	0.9833395	0.9833395	0.9833395	0.9833395	T2 <= T4 <= T1 <= T3
s__Sphaerochaeta_unclassified	0.0187037	0.019321	0.077407	0.046952	0.569452	0.716221	0.726613	T2 <= T4 <= T3 <= T1
s__Odoribacter_laneus	0.0977331	0.0757761	0.3818892	0.3528794	0.3528794	0.3528794	0.8502051	T2 <= T4 <= T3 <= T1
s__Lachnospiraceae_bacterium_7_1_58FAA	0.1732892	0.1956823	0.703702	0.703702	0.3109359	0.3109359	0.8632073	T2 <= T4 <= T3 <= T1
s__Ehrlichia_unclassified	0.1888696	0.1951506	0.4737861	0.4312088	0.5092841	0.5989409	0.7800686	T2 <= T4 <= T3 <= T1
s__Lachnospiraceae_bacterium_3_1_46FAA	0.3489246	0.4828702	0.6090634	0.4828702	0.4828702	0.6090634	0.6090634	T2 <= T4 <= T3 <= T1
s__Lactobacillus_animalis	0.434118	0.5228504	0.891433	0.891433	0.5228504	0.5228504	0.891433	T2 <= T4 <= T3 <= T1
s__Ruminococcaceae_bacterium_D16	0.5617791	0.6995286	0.6995286	0.6995286	0.8770652	0.8770652	0.9297601	T2 <= T4 <= T3 <= T1
s__Caulobacter_unclassified	0.7384296	0.8547721	0.8547721	0.8547721	0.8547721	0.8547721	0.8587363	T2 <= T4 <= T3 <= T1
s__Odoribacter_unclassified	0.8377884	0.868265	0.868265	0.868265	0.868265	0.868265	0.868265	T2 <= T4 <= T3 <= T1
s__Anaerococcus_vaginalis	0.0331677	0.622532	0.622532	0.113884	1	0.032411	0.032411	T2 <=> T3 <= T1 <= T4
s__Ruminococcus_lactaris	0.139982	0.2953341	0.2953341	0.6476215	1	0.1708265	0.1708265	T2 <=> T3 <= T1 <= T4
s__Neisseria_meningitidis	0.295295	0.4942469	0.4942469	0.4942469	1	0.302264	0.302264	T2 <=> T3 <= T1 <= T4
s__Oxalobacter_formigenes	0.5703026	0.4777851	0.4777851	1	1	0.4777851	0.4777851	T2 <=> T3 <= T1 <=> T4
s__Prevotella_amnii	0.5703026	0.4777851	0.4777851	1	1	0.4777851	0.4777851	T2 <=> T3 <= T1 <=> T4
s__Oscillibacter_valericigenes	0.4263688	0.4140144	0.4140144	1	1	0.4140144	0.4140144	T2 <=> T3 <= T4 <=> T1
s__Erysipelotrichaceae_bacterium_5_2_54FAA	0.5703026	0.4777851	0.4777851	1	1	0.4777851	0.4777851	T2 <=> T3 <= T4 <=> T1
s__Pediococcus_pentosaceus	0.1101525	0.0899221	0.0899221	0.0899221	1	1	1	T2 <=> T3 <=> T4 <= T1
s__Acinetobacter_pittii_calcoaceticus_nosocomialis	0.3916252	0.3145984	0.3145984	0.3145984	1	1	1	T2 <=> T3 <=> T4 <= T1
s__Aggregatibacter_segnis	0.3916252	0.3145984	0.3145984	0.3145984	1	1	1	T2 <=> T3 <=> T4 <= T1
s__alpha_proteobacterium_SCGC_AAA024_N17	0.3916252	0.3145984	0.3145984	0.3145984	1	1	1	T2 <=> T3 <=> T4 <= T1
s__Bacteroides_plebeius	0.3916252	0.3145984	0.3145984	0.3145984	1	1	1	T2 <=> T3 <=> T4 <= T1
s__Capnocytophaga_gingivalis	0.3916252	0.3145984	0.3145984	0.3145984	1	1	1	T2 <=> T3 <=> T4 <= T1
s__Erysipelotrichaceae_bacterium_2_2_44A	0.3916252	0.3145984	0.3145984	0.3145984	1	1	1	T2 <=> T3 <=> T4 <= T1
s__Leptotrichia_unclassified	0.3916252	0.3145984	0.3145984	0.3145984	1	1	1	T2 <=> T3 <=> T4 <= T1
s__Meiothermus_unclassified	0.3916252	0.3145984	0.3145984	0.3145984	1	1	1	T2 <=> T3 <=> T4 <= T1
s__Microcoleus_unclassified	0.3916252	0.3145984	0.3145984	0.3145984	1	1	1	T2 <=> T3 <=> T4 <= T1
s__Neisseria_elongata	0.3916252	0.3145984	0.3145984	0.3145984	1	1	1	T2 <=> T3 <=> T4 <= T1
s__Neisseria_macacae	0.3916252	0.3145984	0.3145984	0.3145984	1	1	1	T2 <=> T3 <=> T4 <= T1
s__Neisseria_sicca	0.3916252	0.3145984	0.3145984	0.3145984	1	1	1	T2 <=> T3 <=> T4 <= T1
s__Prevotella_histicola	0.3916252	0.3145984	0.3145984	0.3145984	1	1	1	T2 <=> T3 <=> T4 <= T1
s__Prevotella_timonensis	0.3916252	0.3145984	0.3145984	0.3145984	1	1	1	T2 <=> T3 <=> T4 <= T1
s__Prevotella_veroralis	0.3916252	0.3145984	0.3145984	0.3145984	1	1	1	T2 <=> T3 <=> T4 <= T1
s__Pseudomonas_stutzeri	0.3916252	0.3145984	0.3145984	0.3145984	1	1	1	T2 <=> T3 <=> T4 <= T1
s__Saccharomonospora_unclassified	0.3916252	0.3145984	0.3145984	0.3145984	1	1	1	T2 <=> T3 <=> T4 <= T1
s__Thiomonas_unclassified	0.3916252	0.3145984	0.3145984	0.3145984	1	1	1	T2 <=> T3 <=> T4 <= T1
s__Veillonella_dispar	0.3916252	0.3145984	0.3145984	0.3145984	1	1	1	T2 <=> T3 <=> T4 <= T1
s__zeta_proteobacterium_SCGC_AB_602_E04	0.3916252	0.3145984	0.3145984	0.3145984	1	1	1	T2 <=> T3 <=> T4 <= T1
s__Lactobacillus_crispatus	0.292033	0.4998767	0.4998767	0.4998767	0.2971898	1	0.2971898	T2 <=> T4 <= T1 <= T3
s__Brucella_pinnipedialis	0.5703026	0.4777851	1	0.4777851	0.4777851	1	0.4777851	T2 <=> T4 <= T1 <=> T3
s__Methylotenera_versatilis	0.5703026	0.4777851	1	0.4777851	0.4777851	1	0.4777851	T2 <=> T4 <= T3 <=> T1
s__Streptococcus_mitis_oralis_pneumoniae	0.5703026	0.4777851	1	0.4777851	0.4777851	1	0.4777851	T2 <=> T4 <= T3 <=> T1
s__Desulfovibrio_desulfuricans	0.0055113	0.131702	0.868937	0.012283	0.12238	0.293453	0.012283	T3 <= T1 <= T2 <= T4
s__Comamonas_unclassified	0.0971381	0.5051051	0.468316	0.3556649	0.2083787	0.5873816	0.1102263	T3 <= T1 <= T2 <= T4
s__Candidatus_Arthromitus_sp_SFB_1	0.1527959	0.6061881	0.6061881	0.2937139	0.4349619	0.4349619	0.1678002	T3 <= T1 <= T2 <= T4
s__Listeria_unclassified	0.1560412	0.5986274	0.5986274	0.3003769	0.4275839	0.4275839	0.172499	T3 <= T1 <= T2 <= T4
s__Scardovia_unclassified	0.2582708	0.9722543	0.9873109	0.2691056	0.9722543	0.2903322	0.2691056	T3 <= T1 <= T2 <= T4
s__Aerococcus_viridans	0.3749465	0.7012043	0.9959396	0.3824234	0.7012043	0.6570629	0.3824234	T3 <= T1 <= T2 <= T4
s__Eubacteriaceae_bacterium_CM2	0.5166599	0.6375252	0.6375252	0.6375252	0.6108667	0.9796133	0.6108667	T3 <= T1 <= T2 <= T4
s__Candidatus_Phytoplasma_unclassified	0.5209034	0.6721505	0.9341329	0.6591352	0.6721505	0.6721505	0.6591352	T3 <= T1 <= T2 <= T4
s__Thermodesulfatator_unclassified	0.5576755	0.9943155	0.5793905	0.5793905	0.5793905	0.5793905	0.5793905	T3 <= T1 <= T2 <= T4
s__Exiguobacterium_unclassified	0.5583339	0.7676283	0.7676283	0.7676283	0.7676283	0.7676283	0.7676283	T3 <= T1 <= T2 <= T4
s__Arcobacter_unclassified	0.8329763	0.9890922	0.9183513	0.9890922	0.9183513	0.9890922	0.9183513	T3 <= T1 <= T2 <= T4
s__Bacteroides_caccae	0.0006429	0.004759	0.613466	0.822601	0.000816	0.006688	0.567365	T3 <= T1 <= T4 < T2
s__Gemella_unclassified	0.1641598	0.3210911	0.5986274	0.5986274	0.1822371	0.420291	0.420291	T3 <= T1 <= T4 <= T2
s__Salinicoccus_unclassified	0.5661203	0.5740712	0.5740712	0.9943155	0.5740712	0.5740712	0.5740712	T3 <= T1 <= T4 <= T2
s__Coprococcus_catus	0.886363	0.8709097	0.9906705	0.8709097	0.8709097	0.9906705	0.8709097	T3 <= T1 <= T4 <= T2
s__Aggregatibacter_unclassified	0.9259696	0.9870113	0.9870113	0.9870113	0.9870113	0.9870113	0.9870113	T3 <= T1 <= T4 <= T2
s__Granulicatella_unclassified	0.1889133	1	0.3150789	0.5689975	0.3150789	0.5689975	0.1994685	T3 <= T1 <=> T2 <= T4
s__Cellvibrio_unclassified	0.8144147	1	0.7697697	0.7697697	0.7697697	0.7697697	0.7697697	T3 <= T1 <=> T2 <= T4
s__Lachnospiraceae_bacterium_5_1_57FAA	0.0165424	0.060562	0.045302	0.987097	0.844681	0.060562	0.045302	T3 <= T2 <= T1 <= T4
s__Clostridium_symbiosum	0.0487661	0.389195	0.0964	0.664623	0.370819	0.311716	0.059921	T3 <= T2 <= T1 <= T4
s__Spiribacter_unclassified	0.0513646	0.5090263	0.0758612	0.7579404	0.3009742	0.4022061	0.0655337	T3 <= T2 <= T1 <= T4
s__Parasutterella_excrementihominis	0.0611889	0.917427	0.9156193	0.0869583	0.9156193	0.0869583	0.0869583	T3 <= T2 <= T1 <= T4
s__Marvinbryantia_formatexigens	0.0858601	0.1815115	0.1815115	0.7674653	0.9980639	0.1304991	0.1304991	T3 <= T2 <= T1 <= T4
s__Blautia_hydrogenotrophica	0.1131176	0.5432281	0.2589617	0.5432281	0.4648045	0.3820331	0.1210513	T3 <= T2 <= T1 <= T4
s__Geobacillus_unclassified	0.1258631	0.4006878	0.202841	0.7888068	0.5674081	0.3366737	0.202841	T3 <= T2 <= T1 <= T4
s__Fervidobacterium_unclassified	0.1575533	0.4347419	0.2549489	0.6414747	0.6414747	0.2549489	0.2549489	T3 <= T2 <= T1 <= T4
s__Clostridium_asparagiforme	0.1697773	0.4432984	0.357291	0.638428	0.7650355	0.2841754	0.2841754	T3 <= T2 <= T1 <= T4
s__Lachnospiraceae_bacterium_3_1_57FAA_CT1	0.3181377	0.5197976	0.3741875	0.9682554	0.6631071	0.5197976	0.3741875	T3 <= T2 <= T1 <= T4
s__Anaerostipes_unclassified	0.3314019	0.5766084	0.4845573	0.5766084	0.5766084	0.4845573	0.4845573	T3 <= T2 <= T1 <= T4
s__Ruminococcus_obeum	0.3648509	0.6230372	0.6230372	0.6230372	0.7282475	0.5802572	0.5802572	T3 <= T2 <= T1 <= T4
s__Flavonifractor_plautii	0.4685985	0.5726789	0.5726789	0.9825554	0.8210795	0.5726789	0.5726789	T3 <= T2 <= T1 <= T4
s__Candidatus_Sulcia_muelleri	0.4785093	0.7535097	0.7048767	0.7048767	0.7535097	0.7048767	0.7048767	T3 <= T2 <= T1 <= T4
s__Roseburia_inulinivorans	0.51994	0.6325072	0.6108667	0.9847092	0.6325072	0.6325072	0.6108667	T3 <= T2 <= T1 <= T4
s__Bacteroides_fluxus	0.5293608	0.6526994	0.6246088	0.9796133	0.6526994	0.6526994	0.6246088	T3 <= T2 <= T1 <= T4
s__Clostridium_hathewayi	0.8220769	0.8390916	0.8390916	0.9344349	0.9344349	0.8390916	0.8390916	T3 <= T2 <= T1 <= T4
s__Halomonas_unclassified	0.8223314	0.9626144	0.9350251	0.9626144	0.9350251	0.9626144	0.9350251	T3 <= T2 <= T1 <= T4
s__Lactobacillus_johnsonii	9.72E-10	3.06E-08	3.06E-08	8.14E-06	0.973813	0.315475	0.315475	T3 <= T2 <= T4 < T1
s__Erysipelotrichaceae_bacterium_21_3	0.0014694	0.004391	0.002059	0.029676	0.690241	0.547188	0.378939	T3 <= T2 <= T4 < T1
s__Blautia_producta	0.0334742	0.10121	0.046414	0.493125	0.493125	0.306412	0.10121	T3 <= T2 <= T4 <= T1
s__Eubacterium_plexicaudatum	0.0422872	0.050381	0.050381	0.285775	0.879155	0.425293	0.421477	T3 <= T2 <= T4 <= T1
s__Dorea_longicatena	0.0454672	0.205945	0.040509	0.418153	0.418153	0.418153	0.177128	T3 <= T2 <= T4 <= T1
s__Bartonella_unclassified	0.0591567	0.1368256	0.1368256	0.8214492	0.8214492	0.1655334	0.1368256	T3 <= T2 <= T4 <= T1
s__Lachnospiraceae_bacterium_1_4_56FAA	0.0899032	0.3856355	0.1065835	0.6848102	0.3856355	0.5603866	0.1483835	T3 <= T2 <= T4 <= T1
s__Lachnospiraceae_bacterium_4_1_37FAA	0.1207059	0.5368953	0.112787	0.5368953	0.2524118	0.951722	0.2524118	T3 <= T2 <= T4 <= T1
s__Ruminococcus_gnavus	0.121942	0.2724363	0.1217638	0.3843722	0.6131621	0.6131621	0.3843722	T3 <= T2 <= T4 <= T1
s__Thermanaerovibrio_unclassified	0.1437475	0.3791962	0.1351708	0.4088567	0.5418269	0.7306346	0.4088567	T3 <= T2 <= T4 <= T1
s__Blattabacterium_unclassified	0.2117605	0.268197	0.268197	0.9488512	0.9488512	0.268197	0.268197	T3 <= T2 <= T4 <= T1
s__Helicobacter_pullorum	0.3306363	0.5468271	0.4680713	0.5870694	0.6682441	0.6682441	0.5870694	T3 <= T2 <= T4 <= T1
s__Oribacterium_sp_oral_taxon_078	0.5167734	0.6375227	0.6006986	0.9949028	0.6375227	0.6375227	0.6006986	T3 <= T2 <= T4 <= T1
s__Anaerococcus_prevotii	0.5218399	0.7355915	0.5860154	0.9966508	0.7355915	0.7355915	0.5860154	T3 <= T2 <= T4 <= T1
s__Butyricicoccus_pullicaecorum	0.5474035	0.6534573	0.6534573	0.8227391	0.8227391	0.6738434	0.6534573	T3 <= T2 <= T4 <= T1
s__Clostridium_hylemonae	0.5577	0.7164763	0.7164763	0.7164763	0.7855568	0.9731398	0.7855568	T3 <= T2 <= T4 <= T1
s__Akkermansia_muciniphila	0.0072902	0.675904	0.016847	0.613742	0.008611	0.423806	0.069228	T3 <= T4 <= T1 <= T2
s__Bacteroides_stercoris	0.0356922	0.306814	0.26615	0.306814	0.049927	0.068358	0.717539	T3 <= T4 <= T1 <= T2
s__Enterococcus_gallinarum	0.2711702	0.99784	0.2948833	0.2948833	0.2948833	0.2948833	0.99784	T3 <= T4 <= T1 <= T2
s__Bacteroides_finegoldii	0.3140409	0.6599722	0.6339167	0.6685815	0.4574194	0.5221521	0.6791305	T3 <= T4 <= T1 <= T2
s__Eubacterium_eligens	0.3741419	0.7855579	0.4932655	0.4932655	0.463528	0.463528	0.9884867	T3 <= T4 <= T1 <= T2
s__Bifidobacterium_pseudolongum	0.8601952	0.8480109	0.8480109	0.9678875	0.8480109	0.8480109	0.8480109	T3 <= T4 <= T1 <= T2
s__Enterorhabdus_caecimuris	0.0023923	0.047018	0.003066	0.003066	0.369636	0.369636	0.995864	T3 <= T4 <= T2 < T1
s__Lactobacillus_gasseri	0.0178469	0.343301	0.014693	0.110057	0.110057	0.343301	0.343301	T3 <= T4 <= T2 <= T1
s__Dorea_sp_5_2	0.2177288	0.5902206	0.2962988	0.4025692	0.4025692	0.5902206	0.598506	T3 <= T4 <= T2 <= T1
s__Bacteroides_thetaiotaomicron	0.3967308	0.7439836	0.5621769	0.7299096	0.6536806	0.8416004	0.6536806	T3 <= T4 <= T2 <= T1
s__Pasteurella_pneumotropica	0.6169093	0.7293408	0.7293408	0.7293408	0.7293408	0.7293408	0.928993	T3 <= T4 <= T2 <= T1
s__Faecalibacterium_prausnitzii	0.7932713	0.9546033	0.87161	0.87161	0.87161	0.87161	0.87161	T3 <= T4 <= T2 <= T1
s__Weissella_unclassified	0.9927541	0.987474	0.987474	0.987474	0.987474	0.987474	0.987474	T3 <= T4 <= T2 <= T1
s__Borrelia_bavariensis	0.295295	0.4942469	0.4942469	0.4942469	0.302264	0.302264	1	T3 <=> T4 <= T1 <= T2
s__Clostridium_leptum	0.5703026	1	0.4777851	0.4777851	0.4777851	0.4777851	1	T3 <=> T4 <= T2 <=> T1
s__Methanothermococcus_unclassified	0.5703026	1	0.4777851	0.4777851	0.4777851	0.4777851	1	T3 <=> T4 <= T2 <=> T1
s__Thermoanaerobacter_ethanolicus	0.5703026	1	0.4777851	0.4777851	0.4777851	0.4777851	1	T3 <=> T4 <= T2 <=> T1
s__Treponema_primitia	0.5703026	1	0.4777851	0.4777851	0.4777851	0.4777851	1	T3 <=> T4 <= T2 <=> T1
s__Candidatus_Arthromitus_unclassified	9.34E-11	0.001128	0.001711	1.17E-11	0.856686	0.000489	0.000356	T4 < T2 <= T3 < T1
s__Mucispirillum_schaedleri	1.14E-09	0.312769	0.805505	1.51E-06	0.255513	4.63E-09	3.52E-06	T4 < T3 <= T1 <= T2
s__Lactobacillus_reuteri	2.81E-08	0.001586	0.001586	4.58E-09	0.995864	0.006286	0.006286	T4 < T3 <= T2 < T1
s__Alistipes_unclassified	5.47E-08	0.02265	0.009455	1.14E-08	0.701306	0.000764	0.00212	T4 < T3 <= T2 < T1
s__Clostridium_sp_ASF356	3.00E-05	0.43387	0.05989	3.47E-05	0.244082	0.000527	0.026303	T4 < T3 <= T2 <= T1
s__Bacteroides_ovatus	0.0722914	0.4731511	0.2198817	0.4731511	0.4731511	0.2515324	0.0727763	T4 <= T1 <= T2 <= T3
s__Candidatus_Uzinura_diaspidicola	0.8833284	0.858935	0.858935	0.9953352	0.9953352	0.858935	0.858935	T4 <= T1 <= T2 <= T3
s__Bacteroides_cellulosilyticus	0.4507613	0.4521876	0.9225196	0.9225196	0.4521876	0.4521876	0.9225196	T4 <= T1 <= T3 <= T2
s__Clostridium_pasteurianum	0.6652936	0.7365494	0.7365494	0.9913406	0.7365494	0.7365494	0.7365494	T4 <= T1 <= T3 <= T2
s__Oscillibacter_sp_1_3	0.7598241	0.8297271	0.8297271	0.8297271	0.8297271	0.8297271	0.8297271	T4 <= T1 <= T3 <= T2
s__Prevotella_copri	0.0040525	0.993302	0.01851	0.504339	0.01851	0.504339	0.003802	T4 <= T2 <= T1 < T3
s__Brevibacterium_unclassified	0.2494679	0.8133839	0.9651192	0.2523115	0.8133839	0.3790973	0.2523115	T4 <= T2 <= T1 <= T3
s__Marinobacter_unclassified	0.7987468	0.9934526	0.9934526	0.8331278	0.9934526	0.8331278	0.8331278	T4 <= T2 <= T1 <= T3
s__Alistipes_putredinis	0.001394	0.037732	0.045882	0.000604	0.825016	0.196109	0.159549	T4 <= T2 <= T3 < T1
s__Eubacterium_rectale	0.1651414	0.3467491	0.489023	0.1847655	0.5577851	0.5577851	0.4779523	T4 <= T2 <= T3 <= T1
s__Lactobacillus_murinus	0.2290137	0.9706448	0.9765683	0.2402528	0.9706448	0.2629482	0.2402528	T4 <= T2 <= T3 <= T1
s__Spiroplasma_unclassified	0.3464167	0.3709446	0.5245234	0.3709446	0.8106293	0.9477627	0.8106293	T4 <= T2 <= T3 <= T1
s__Pusillimonas_unclassified	0.3795504	0.3190924	0.3190924	0.3190924	0.9912093	0.9912093	0.9912093	T4 <= T2 <= T3 <= T1
s__Proteus_unclassified	0.5166599	0.6375252	0.9796133	0.6108667	0.6375252	0.6375252	0.6108667	T4 <= T2 <= T3 <= T1
s__Leuconostoc_unclassified	0.7361521	0.9026709	0.9521475	0.9026709	0.9026709	0.9026709	0.9026709	T4 <= T2 <= T3 <= T1
s__Adlercreutzia_equolifaciens	0.0069853	0.136914	0.367435	0.27638	0.013314	0.009804	0.76072	T4 <= T3 <= T1 <= T2
s__Gardnerella_vaginalis	0.082383	0.1861913	0.7343549	0.7133227	0.1305778	0.1003303	0.7343549	T4 <= T3 <= T1 <= T2
s__Helicobacter_cinaedi	0.2686833	0.7534925	0.7534925	0.3832477	0.7534925	0.3786509	0.4533255	T4 <= T3 <= T1 <= T2
s__Bacteroides_oleiciplenus	0.4160652	0.6311133	0.8527798	0.7925976	0.6311133	0.6206035	0.7925976	T4 <= T3 <= T1 <= T2
s__Lactobacillus_jensenii	0.5661203	0.5740712	0.9943155	0.5740712	0.5740712	0.5740712	0.5740712	T4 <= T3 <= T1 <= T2
s__Helicobacter_hepaticus	0.8528996	0.9394029	0.8725046	0.8725046	0.8725046	0.8725046	0.8725046	T4 <= T3 <= T1 <= T2
s__Proteus_mirabilis	0.0006979	0.015618	0.001338	0.001338	0.473722	0.473722	0.923048	T4 <= T3 <= T2 < T1
s__Anaerotruncus_colihominis	0.0002173	0.125393	0.000882	0.000862	0.068714	0.058766	0.856241	T4 <= T3 <= T2 <= T1
s__Coprobacillus_unclassified	0.0002425	0.205683	0.054707	0.000108	0.469112	0.010501	0.054707	T4 <= T3 <= T2 <= T1
s__Enterococcus_faecalis	0.0569599	0.7147347	0.2526124	0.0851397	0.3670454	0.1106877	0.4268416	T4 <= T3 <= T2 <= T1
s__Pediococcus_unclassified	0.2670143	0.5713444	0.4328264	0.3475668	0.6947706	0.6110131	0.6947706	T4 <= T3 <= T2 <= T1
s__Eubacterium_sp_14_2	0.5661203	0.5740712	0.5740712	0.5740712	0.9943155	0.5740712	0.5740712	T4 <= T3 <= T2 <= T1

Supplementary Table 6 | The Wilcoxon rank-sum test for significantly changed gut microbiota species between OVX and control group mice.

		p_value	Q-Value(BH correction)	rm_Model	rm_Normal	or_Model	or_Normal	Enrichment
T1	s__Veillonella_unclassified	0.0339883	0.088369681	31.28	38.733333	0.1	0.3333333	OVX < Control
	s__Escherichia_coli	0.9686698	0.968669769	33.06	32.8	0.74	0.7333333	Control <= OVX
	s__Firmicutes_bacterium_M10_2	0.4504867	0.610831546	33.3	32	0.04	0	Control <= OVX
	s__Dysgonomonas_unclassified	0.2230901	0.362521367	34.54	27.866667	0.64	0.6666667	Control <= OVX
	s__Parabacteroides_unclassified	0.0002174	0.001413204	37.76	17.133333	1	1	Control < OVX
	s__Parabacteroides_distasonis	4.47E-05	0.0005811	38.24	15.533333	0.9	0.6	Control < OVX
	s__Paraprevotella_unclassified	0.0306321	0.088369681	34.95	26.5	0.26	0	Control < OVX
	s__Dorea_unclassified	0.6092056	0.659972747	33.15	32.5	0.02	0	Control <= OVX
	s__Peptostreptococcaceae_noname_unclassified	0.0034316	0.014870405	36.75	20.5	0.82	0.6666667	Control < OVX
	s__Lachnospiraceae_bacterium_8_1_57FAA	0.1542575	0.286478237	34.84	26.866667	1	1	Control <= OVX
	s__Bacteroides_xylanisolvens	0.5168575	0.610831546	33.84	30.2	0.78	0.9333333	Control <= OVX
	s__Desulfovibrio_termitidis	0.1324368	0.286478237	34.05	29.5	0.14	0	Control <= OVX
	s__Bacteroides_uniformis	0.4786758	0.610831546	33.92	29.933333	0.98	1	Control <= OVX
	s__Acinetobacter_unclassified	NaN	NaN	33	33	0	0	OVX <=> Control
	s__Eubacterium_cellulosolvens	NaN	NaN	33	33	0	0	OVX <=> Control
T2	s__Veillonella_unclassified	0.0140691	0.0307581	36.16	22.466667	0.96	0.5333333	Control < OVX
	s__Escherichia_coli	1.47E-06	0.00002205	39.18	12.4	0.92	0.5333333	Control < OVX
	s__Firmicutes_bacterium_M10_2	0.7892999	0.8456785	33.32	31.933333	0.46	0.3333333	Control <= OVX
	s__Dysgonomonas_unclassified	1.36E-05	0.000102	27.42	51.6	0.76	1	OVX < Control
	s__Parabacteroides_unclassified	0.7495866	0.8456785	32.58	34.4	1	1	OVX <= Control
	s__Parabacteroides_distasonis	0.9069169	0.90691691	33.16	32.466667	0.84	0.9333333	Control <= OVX
	s__Paraprevotella_unclassified	0.6384077	0.79800965	32.45	34.833333	0.42	0.4	OVX <= Control
	s__Dorea_unclassified	0.1520788	0.25346471	32	36.333333	0.08	0.2	OVX <= Control
	s__Peptostreptococcaceae_noname_unclassified	0.0068836	0.02065071	29.52	44.6	0.88	1	OVX < Control
	s__Lachnospiraceae_bacterium_8_1_57FAA	0.5131464	0.76971965	33.85	30.166667	0.98	0.9333333	Control <= OVX
	s__Bacteroides_xylanisolvens	0.046873	0.08788684	35.54	24.533333	0.8	0.5333333	Control < OVX
	s__Desulfovibrio_termitidis	0.0043354	0.01625781	30.1	42.666667	0.2	0.5333333	OVX < Control
	s__Bacteroides_uniformis	0.0016157	0.00807847	37.06	19.466667	0.98	0.8666667	Control < OVX
	s__Acinetobacter_unclassified	0.6092056	0.79800965	33.15	32.5	0.02	0	Control <= OVX
	s__Eubacterium_cellulosolvens	0.0143538	0.0307581	31.68	37.4	0.02	0.2	OVX < Control
T3	s__Veillonella_unclassified	0.0004652	0.002325835	37.5	18	0.92	0.6666667	Control < OVX
	s__Escherichia_coli	4.82E-07	0.00000723	39.46	11.466667	0.94	0.4666667	Control < OVX
	s__Firmicutes_bacterium_M10_2	0.1076288	0.230633128	34.64	27.533333	0.32	0.1333333	Control <= OVX
	s__Dysgonomonas_unclassified	0.0001228	0.000921128	28.06	49.466667	0.86	1	OVX < Control
	s__Parabacteroides_unclassified	0.1542395	0.28919907	31.16	39.133333	0.94	1	OVX <= Control
	s__Parabacteroides_distasonis	0.4831202	0.572870392	32.09	36.033333	0.86	0.9333333	OVX <= Control
	s__Paraprevotella_unclassified	0.3772324	0.514407822	31.94	36.533333	0.48	0.4666667	OVX <= Control
	s__Dorea_unclassified	0.6126159	0.612615928	33.36	31.8	0.12	0.0666667	Control <= OVX
	s__Peptostreptococcaceae_noname_unclassified	0.0013487	0.005057546	28.88	46.733333	0.8	1	OVX < Control
	s__Lachnospiraceae_bacterium_8_1_57FAA	0.3152516	0.472877333	31.7	37.333333	0.98	1	OVX <= Control
	s__Bacteroides_xylanisolvens	0.4964877	0.572870392	33.88	30.066667	0.84	0.6666667	Control <= OVX
	s__Desulfovibrio_termitidis	0.244633	0.407721625	31.86	36.8	0.22	0.3333333	OVX <= Control
	s__Bacteroides_uniformis	0.0079377	0.023812956	36.42	21.6	0.98	1	Control < OVX
	s__Acinetobacter_unclassified	0.6092056	0.612615928	33.15	32.5	0.02	0	Control <= OVX
	s__Eubacterium_cellulosolvens	0.0791239	0.197809775	32.17	35.766667	0.02	0.1333333	OVX <= Control
T4	s__Veillonella_unclassified	5.35E-06	4.19E-05	38.85	13.5	0.98	0.5333333	Control < OVX
	s__Escherichia_coli	5.59E-06	4.19E-05	38.84	13.533333	1	0.5333333	Control < OVX
	s__Firmicutes_bacterium_M10_2	0.0089679	0.044839715	36.23	22.233333	0.66	0.2666667	Control < OVX
	s__Dysgonomonas_unclassified	0.8032472	0.833497777	32.67	34.1	0.94	0.9333333	OVX <= Control
	s__Parabacteroides_unclassified	0.1730755	0.324516482	34.76	27.133333	0.96	1	Control <= OVX
	s__Parabacteroides_distasonis	0.1497153	0.320818457	34.86	26.8	0.92	0.9333333	Control <= OVX
	s__Paraprevotella_unclassified	0.030384	0.113940124	35.62	24.266667	0.56	0.3333333	Control < OVX
	s__Dorea_unclassified	0.1438519	0.320818457	34.36	28.466667	0.26	0.0666667	Control <= OVX
	s__Peptostreptococcaceae_noname_unclassified	0.7968939	0.833497777	33.34	31.866667	0.88	0.7333333	Control <= OVX
	s__Lachnospiraceae_bacterium_8_1_57FAA	0.8334978	0.833497777	33.28	32.066667	0.92	1	Control <= OVX
	s__Bacteroides_xylanisolvens	0.8208331	0.833497777	33.3	32	0.8	0.8666667	Control <= OVX
	s__Desulfovibrio_termitidis	0.0389377	0.116813064	35.06	26.133333	0.32	0.0666667	Control < OVX
	s__Bacteroides_uniformis	0.3228078	0.471988473	34.28	28.733333	0.96	1	Control <= OVX
	s__Acinetobacter_unclassified	0.3461249	0.471988473	33.45	31.5	0.06	0	Control <= OVX
	s__Eubacterium_cellulosolvens	0.3461249	0.471988473	33.45	31.5	0.06	0	Control <= OVX

Supplementary Table 7 | The mice's weight is strongly associated with gut microbiota species.

Species	Clincal index	Sparman correlation	p-value	Enrichment
s__Lactobacillus_vaginalis	Weight	0.3050	5.31E-07	Irregular
s__Lachnospiraceae_bacterium_8_1_57FAA	Weight	0.3321	4.11E-08	Irregular
s__Escherichia_coli	Weight	0.3916	5.84E-11	OVX
s__Lactobacillus_hominis	Weight	0.4309	3.51E-13	Irregular
s__Veillonella_unclassified	Weight	0.5892	1.09E-25	OVX
s__Candidatus_Arthromitus_unclassified	Weight	-0.3221	1.09E-07	Irregular

Supplementary Table 8 | The results of the GMM classification.

	GMM Modules	p_value	rm_OVX	rm_Control	or_OVX	or_Control	Enrichment	Q-Value(BH correction)
	mucin degradation	0.0008868	28.72	47.26666667	1	1	OVX < Control	0.05693439
	fructan degradation	0.0021045	29.04	46.2	1	1	OVX < Control	0.05693439
	glyoxylate bypass	0.0022484	36.93	19.9	1	1	Control < OVX	0.05693439
	acetyl-CoA to acetate	0.0027863	36.85	20.16666667	1	1	Control < OVX	0.05693439
	alanine degradation II	0.0035045	29.24	45.53333333	1	1	OVX < Control	0.05693439
	hydrogen metabolism	0.0038625	36.72	20.6	1	1	Control < OVX	0.05693439
	tryptophan degradation	0.0042725	29.32	45.26666667	1	1	OVX < Control	0.05693439
	xylose degradation	0.0044654	29.34	45.2	1	1	OVX < Control	0.05693439
	lactate consumption I	0.007232	29.54	44.53333333	1	1	OVX < Control	0.08196266
	formate conversion	0.0135623	36.18	22.4	1	1	Control < OVX	0.13833589
	NADH:ferredoxin oxidoreductase	0.017943	36.05	22.83333333	1	1	Control < OVX	0.14658707
	glycine degradation	0.0190664	36.02	22.93333333	1	1	Control < OVX	0.14658707
	pentose phosphate pathway (non-oxidativ	0.0210968	30.03	42.9	1	1	OVX < Control	0.14658707
	triacylglycerol degradation	0.0224849	35.94	23.2	1	1	Control < OVX	0.14658707
	Entner-Doudoroff pathway	0.0234665	35.92	23.26666667	1	1	Control < OVX	0.14658707
	glycerol degradation III	0.0239246	30.09	42.7	1	1	OVX < Control	0.14658707
	putrescine degradation	0.0244312	30.1	42.66666667	1	1	OVX < Control	0.14658707
	propionate production II	0.0292584	30.19	42.36666667	1	1	OVX < Control	0.16579749
	aspartate degradation I	0.034804	35.72	23.93333333	1	1	Control < OVX	0.1868424
	homoacetogenesis	0.0405325	30.36	41.8	1	1	OVX < Control	0.19581138
	tyrosine degradation I	0.0436269	35.6	24.33333333	1	1	Control < OVX	0.19581138
	phenylalanine degradation	0.0460302	35.57	24.43333333	1	1	Control < OVX	0.19581138
	lactate consumption II	0.0478686	30.45	41.5	1	1	OVX < Control	0.19581138
	allose degradation	0.0479808	35.55	24.5	1	1	Control < OVX	0.19581138
	methanogenesis - methyl-coM	0.047993	35.55	24.5	1	1	Control < OVX	0.19581138
	glutamate degradation I	0.0534212	30.51	41.3	0.9	0.9333333	OVX <= Control	0.20928076
	sulfate reduction (dissimilatory)	0.0574802	35.45	24.83333333	1	1	Control <= OVX	0.20928076
	nitrate reduction (dissimilatory)	0.0595193	30.57	41.1	1	1	OVX <= Control	0.20928076
	threonine degradation II	0.0605993	35.42	24.93333333	1	1	Control <= OVX	0.20928076
	glycerol degradation I	0.0615532	30.59	41.03333333	1	1	OVX <= Control	0.20928076
	succinate conversion to propionate	0.0637359	30.61	40.96666667	0.88	0.9333333	OVX <= Control	0.20971152
	urea degradation	0.0661215	35.37	25.1	1	1	Control <= OVX	0.21076239
	alanine degradation I	0.0733125	30.69	40.7	1	1	OVX <= Control	0.21755834
	methionine degradation II	0.0745742	35.3	25.33333333	1	1	Control <= OVX	0.21755834
	anaerobic fatty acid beta-oxidation	0.07586	35.29	25.36666667	1	1	Control <= OVX	0.21755834
	arginine degradation IV	0.0771798	30.72	40.6	1	1	OVX <= Control	0.21755834
	aspartate degradation II	0.0798223	35.26	25.46666667	1	1	Control <= OVX	0.21755834
	arginine degradation I	0.0810511	35.25	25.5	1	1	Control <= OVX	0.21755834
	Bifidobacterium shunt	0.090878	30.82	40.26666667	1	1	OVX <= Control	0.23768093
	glycerol degradation II	0.0940207	30.84	40.2	1	1	OVX <= Control	0.23975267
	lysine degradation II	0.1175777	35.02	26.26666667	1	1	Control <= OVX	0.29251033
	arabinoxylan degradation	0.128996	31.04	39.53333333	1	1	OVX <= Control	0.31327603
	4-aminobutyrate degradation	0.147482	34.87	26.76666667	1	1	Control <= OVX	0.34984112
	cysteine biosynthesis/homocysteine degrac	0.1563132	34.83	26.9	1	1	Control <= OVX	0.36236242
	glutamate degradation III	0.1611021	34.81	26.96666667	1	1	Control <= OVX	0.36516468
	glycolysis (preparatory phase)	0.1703524	31.23	38.9	1	1	OVX <= Control	0.37773798
	pentose phosphate pathway (oxidative pha	0.1779915	34.74	27.2	1	1	Control <= OVX	0.38627938
	starch degradation	0.1882871	34.7	27.33333333	1	1	Control <= OVX	0.4001101
	fructose degradation	0.1961918	31.33	38.56666667	1	1	OVX <= Control	0.40839916
	propionate production III	0.2072184	34.63	27.56666667	1	1	Control <= OVX	0.42272561
	leucine degradation	0.2145539	34.6	27.66666667	1	1	Control <= OVX	0.42600977
T1	lactaldehyde degradation	0.221353	31.42	38.26666667	1	1	OVX <= Control	0.42600977
	arginine degradation III	0.221358	34.58	27.73333333	0.9	0.9333333	Control <= OVX	0.42600977
	pyruvate dehydrogenase complex	0.2427435	34.51	27.96666667	1	1	Control <= OVX	0.45851544
	lysine degradation I	0.2757316	34.41	28.3	1	1	Control <= OVX	0.51135681
	asparagine degradation	0.2826649	34.39	28.36666667	1	1	Control <= OVX	0.51485399
	succinate consumption	0.2932549	34.36	28.46666667	1	1	Control <= OVX	0.52477193
	cysteine degradation I	0.3151775	34.3	28.66666667	1	1	Control <= OVX	0.5542777
	ribose degradation	0.3302645	31.74	37.2	0.92	0.9333333	OVX <= Control	0.57096577
	pectine degradation II	0.395995	34.1	29.33333333	1	1	Control <= OVX	0.66237133
	lactose degradation	0.396124	31.9	36.66666667	1	1	OVX <= Control	0.66237133
	cysteine degradation II	0.4313688	34.02	29.6	1	1	Control <= OVX	0.70967118
	pyruvate:ferredoxin oxidoreductase	0.4408316	34	29.66666667	1	1	Control <= OVX	0.71372729
	fucose degradation	0.4687473	32.06	36.13333333	1	1	OVX <= Control	0.73583978
	maltose degradation	0.4689175	33.94	29.86666667	1	1	Control <= OVX	0.73583978
	trehalose degradation	0.5180927	33.84	30.2	1	1	Control <= OVX	0.80068865
	methanogenesis from carbon dioxide	0.5333986	33.81	30.3	1	1	Control <= OVX	0.81203969
	threonine degradation I	0.5486364	32.22	35.6	1	1	OVX <= Control	0.81904104
	proline degradation	0.5540572	32.23	35.56666667	1	1	OVX <= Control	0.81904104
	pyruvate:formate lyase	0.5857229	32.29	35.36666667	1	1	OVX <= Control	0.8534819
	sucrose degradation I	0.6072636	32.33	35.23333333	1	1	OVX <= Control	0.87240683
	rhamnose degradation	0.6235734	33.64	30.86666667	1	1	Control <= OVX	0.87932285
	glutamine degradation I	0.6293193	32.37	35.1	1	1	OVX <= Control	0.87932285
	serine degradation	0.6403923	33.61	30.96666667	1	1	Control <= OVX	0.88195582
	glutamine degradation II	0.6515583	32.41	34.96666667	1	1	OVX <= Control	0.88195582
	arginine degradation II	0.6571923	33.58	31.06666667	1	1	Control <= OVX	0.88195582
	acetate to acetyl-CoA	0.6741372	32.45	34.83333333	1	1	OVX <= Control	0.88195582
	lactose and galactose degradation	0.6855777	33.53	31.23333333	1	1	Control <= OVX	0.88195582
	arginine degradation V	0.7025489	33.5	31.33333333	1	1	Control <= OVX	0.88195582
	propionate production I	0.7028401	33.5	31.33333333	1	1	Control <= OVX	0.88195582
	galactose degradation	0.7143423	33.48	31.4	1	1	Control <= OVX	0.88195582
	valine degradation I	0.7200943	33.47	31.43333333	1	1	Control <= OVX	0.88195582
	arabinose degradation	0.725937	33.46	31.46666667	1	1	Control <= OVX	0.88195582
	mannose degradation	0.7318914	33.45	31.5	1	1	Control <= OVX	0.88195582
	glycolysis (pay-off phase)	0.7436044	32.57	34.43333333	1	1	OVX <= Control	0.88195582
	ethanol production II	0.7436098	32.57	34.43333333	1	1	OVX <= Control	0.88195582
	tyrosine degradation II	0.7554788	33.41	31.63333333	1	1	Control <= OVX	0.88300086
	methionine degradation I	0.7733126	32.62	34.26666667	1	1	OVX <= Control	0.88300086
	butyrate production I	0.7791043	32.63	34.23333333	1	1	OVX <= Control	0.88300086
	sucrose degradation II	0.7791184	32.63	34.23333333	1	1	OVX <= Control	0.88300086
	acetyl-CoA to crotonyl-CoA	0.8334691	32.72	33.93333333	1	1	OVX <= Control	0.92788567
	galacturonate degradation I	0.8394805	33.27	32.1	1	1	Control <= OVX	0.92788567
	ethanol production I	0.8517703	33.25	32.16666667	1	1	Control <= OVX	0.92788567
	histidine degradation	0.8578486	32.76	33.8	1	1	OVX <= Control	0.92788567
	methanol conversion	0.8701058	33.22	32.26666667	1	1	Control <= OVX	0.92788567
	lactate production	0.8823921	33.2	32.33333333	1	1	Control <= OVX	0.92788567
	glutamate degradation II	0.8824011	33.2	32.33333333	1	1	Control <= OVX	0.92788567
	succinate production	0.8947036	33.18	32.4	1	1	Control <= OVX	0.93122206
	isoleucine degradation	0.9440859	33.1	32.66666667	1	1	Control <= OVX	0.96302484
	melibiose degradation	0.944142	32.9	33.33333333	1	1	OVX <= Control	0.96302484
	butyrate production II	0.9627151	32.93	33.23333333	1	1	OVX <= Control	0.97224692
	pectin degradation I	0.9813671	33.04	32.86666667	1	1	Control <= OVX	0.98136713
	galacturonate degradation II	NaN	33	33	0	0	OVX <=> Control	NaN
	aspartate degradation II	0.0002849	37.67	17.43333333	1	1	Control < OVX	0.01388852
	arabinoxylan degradation	0.0003968	37.56	17.8	1	1	Control < OVX	0.01388852
	Entner-Doudoroff pathway	0.0004085	28.45	48.16666667	1	1	OVX < Control	0.01388852
	butyrate production I	0.0014055	28.89	46.7	1	1	OVX < Control	0.03584002
	threonine degradation II	0.0032429	36.79	20.36666667	1	1	Control < OVX	0.06615512
	ethanol production I	0.0042632	36.68	20.73333333	1	1	Control < OVX	0.07247518
	ethanol production II	0.0064194	36.51	21.3	1	1	Control < OVX	0.09354034
	pectine degradation II	0.0104066	36.3	22	1	1	Control < OVX	0.1246192
	starch degradation	0.0124551	36.22	22.26666667	1	1	Control < OVX	0.1246192
	pectin degradation I	0.013593	36.18	22.4	1	1	Control < OVX	0.1246192
	lactate consumption I	0.0161465	29.9	43.33333333	1	1	OVX < Control	0.1246192
	arabinose degradation	0.0175505	36.06	22.8	1	1	Control < OVX	0.1246192
	succinate consumption	0.0183099	36.04	22.86666667	1	1	Control < OVX	0.1246192
	glycolysis (pay-off phase)	0.0186796	29.97	43.1	1	1	OVX < Control	0.1246192
	cysteine degradation II	0.019872	30	43	1	1	OVX < Control	0.1246192
	acetate to acetyl-CoA	0.0199058	30	43	1	1	OVX < Control	0.1246192
	mucin degradation	0.0207699	35.98	23.06666667	1	1	Control < OVX	0.1246192
	fructan degradation	0.027584	35.84	23.53333333	1	1	Control < OVX	0.14451348
	formate conversion	0.028653	35.82	23.6	1	1	Control < OVX	0.14451348
	pyruvate dehydrogenase complex	0.0292213	30.19	42.36666667	1	1	OVX < Control	0.14451348
	succinate conversion to propionate	0.0297528	30.2	42.33333333	0.88	0.9333333	OVX < Control	0.14451348
	maltose degradation	0.0355136	30.29	42.03333333	1	1	OVX < Control	0.16465418
	galactose degradation	0.0412858	30.37	41.76666667	1	1	OVX < Control	0.18309343
	arginine degradation III	0.0443012	30.42	41.6	0.76	0.8666667	OVX < Control	0.1882799
	NADH:ferredoxin oxidoreductase	0.0638781	35.39	25.03333333	1	1	Control <= OVX	0.2606225
	histidine degradation	0.0696195	30.66	40.8	1	1	OVX <= Control	0.27312261
	glycine degradation	0.0824103	30.76	40.46666667	1	1	OVX <= Control	0.31083651
	sulfate reduction (dissimilatory)	0.0853277	35.22	25.6	1	1	Control <= OVX	0.31083651
	methanogenesis - methyl-coM	0.0988538	30.87	40.1	1	1	OVX <= Control	0.34684603
	allose degradation	0.1020135	30.89	40.03333333	1	1	OVX <= Control	0.34684603
	tyrosine degradation II	0.1070417	30.92	39.93333333	1	1	OVX <= Control	0.35220163
	lactose degradation	0.1213305	35	26.33333333	1	1	Control <= OVX	0.38043396
	lactaldehyde degradation	0.1230816	34.99	26.36666667	1	1	Control <= OVX	0.38043396
	acetyl-CoA to acetate	0.1289166	34.96	26.46666667	1	1	Control <= OVX	0.3867497
	acetyl-CoA to crotonyl-CoA	0.1497019	31.14	39.2	1	1	OVX <= Control	0.41938299
	nitrate reduction (dissimilatory)	0.1519513	34.85	26.83333333	1	1	Control <= OVX	0.41938299
	arginine degradation II	0.1541855	34.84	26.86666667	1	1	Control <= OVX	0.41938299
	butyrate production II	0.1562407	31.17	39.1	1	1	OVX <= Control	0.41938299
	melibiose degradation	0.1682208	34.78	27.06666667	1	1	Control <= OVX	0.43996222
	triacylglycerol degradation	0.177816	34.74	27.2	1	1	Control <= OVX	0.45343081
	tyrosine degradation I	0.1828155	31.28	38.73333333	1	1	OVX <= Control	0.45480934
	isoleucine degradation	0.1934327	31.32	38.6	1	1	OVX <= Control	0.46976508
	aspartate degradation I	0.207144	31.37	38.43333333	1	1	OVX <= Control	0.49136475
	leucine degradation	0.2207055	31.42	38.26666667	1	1	OVX <= Control	0.50424705
	hydrogen metabolism	0.2245221	34.57	27.76666667	1	1	Control <= OVX	0.50424705
	methionine degradation II	0.2274055	34.56	27.8	1	1	Control <= OVX	0.50424705
	trehalose degradation	0.2334648	34.54	27.86666667	1	1	Control <= OVX	0.50666832
	asparagine degradation	0.2492271	34.49	28.03333333	1	1	Control <= OVX	0.52960752
	xylose degradation	0.2655003	34.44	28.2	1	1	Control <= OVX	0.55267415
	serine degradation	0.2826176	31.61	37.63333333	1	1	OVX <= Control	0.56104957
	glutamate degradation III	0.2826544	34.39	28.36666667	1	1	Control <= OVX	0.56104957
T2	glycolysis (preparatory phase)	0.2860253	34.38	28.4	1	1	Control <= OVX	0.56104957
	glutamate degradation I	0.3036586	31.67	37.43333333	0.88	0.8666667	OVX <= Control	0.5843995
	pentose phosphate pathway (non-oxidativ	0.3303491	31.74	37.2	1	1	OVX <= Control	0.62399279
	propionate production III	0.350154	31.79	37.03333333	1	1	OVX <= Control	0.6445311
	ribose degradation	0.3571102	31.81	36.96666667	0.78	1	OVX <= Control	0.6445311
	tryptophan degradation	0.3664738	31.83	36.9	1	1	OVX <= Control	0.6445311
	cysteine degradation I	0.3747114	34.15	29.16666667	1	1	Control <= OVX	0.6445311
	sucrose degradation I	0.3830707	34.13	29.23333333	1	1	Control <= OVX	0.6445311
	Bifidobacterium shunt	0.3871458	31.88	36.73333333	1	1	OVX <= Control	0.6445311
	fructose degradation	0.3874054	31.88	36.73333333	1	1	OVX <= Control	0.6445311
	pyruvate:ferredoxin oxidoreductase	0.3917738	34.11	29.3	1	1	Control <= OVX	0.6445311
	arginine degradation V	0.4001834	31.91	36.63333333	1	1	OVX <= Control	0.64513679
	propionate production I	0.4047917	34.08	29.4	1	1	Control <= OVX	0.64513679
	glyoxylate bypass	0.4177659	34.05	29.5	1	1	Control <= OVX	0.65342786
	lactose and galactose degradation	0.4316659	34.02	29.6	1	1	Control <= OVX	0.65342786
	lysine degradation II	0.4500438	32.02	36.26666667	1	1	OVX <= Control	0.65342786
	glutamate degradation II	0.450118	32.02	36.26666667	1	1	OVX <= Control	0.65342786
	succinate production	0.4501378	33.98	29.73333333	1	1	Control <= OVX	0.65342786
	pyruvate:formate lyase	0.4547927	33.97	29.76666667	1	1	Control <= OVX	0.65342786
	proline degradation	0.454837	33.97	29.76666667	1	1	Control <= OVX	0.65342786
	mannose degradation	0.4931948	33.89	30.03333333	1	1	Control <= OVX	0.6961356
	arginine degradation IV	0.4982147	32.12	35.93333333	1	1	OVX <= Control	0.6961356
	arginine degradation I	0.5230199	33.83	30.23333333	1	1	Control <= OVX	0.72091935
	pentose phosphate pathway (oxidative pha	0.5644442	32.25	35.5	1	1	OVX <= Control	0.75754927
	threonine degradation I	0.5644485	33.75	30.5	1	1	Control <= OVX	0.75754927
	glycerol degradation I	0.5803254	32.28	35.4	1	1	OVX <= Control	0.76874273
	methanol conversion	0.6073068	33.67	30.76666667	1	1	Control <= OVX	0.79417042
	valine degradation I	0.6180581	32.35	35.16666667	1	1	OVX <= Control	0.797999
	cysteine biosynthesis/homocysteine degrac	0.679837	33.54	31.2	1	1	Control <= OVX	0.86331189
	urea degradation	0.6855712	32.47	34.76666667	1	1	OVX <= Control	0.86331189
	lactate consumption II	0.7084512	33.49	31.36666667	1	1	Control <= OVX	0.87123144
	glycerol degradation III	0.7143632	33.48	31.4	1	1	Control <= OVX	0.87123144
	phenylalanine degradation	0.7201002	32.53	34.56666667	1	1	OVX <= Control	0.87123144
	lactate production	0.7260262	33.46	31.46666667	1	1	Control <= OVX	0.87123144
	alanine degradation I	0.7613659	32.6	34.33333333	1	1	OVX <= Control	0.89268036
	anaerobic fatty acid beta-oxidation	0.7614038	33.4	31.66666667	1	1	Control <= OVX	0.89268036
	lysine degradation I	0.7792619	32.63	34.23333333	1	1	OVX <= Control	0.89990509
	glycerol degradation II	0.7852113	33.36	31.8	1	1	Control <= OVX	0.89990509
	putrescine degradation	0.8213384	33.3	32	1	1	Control <= OVX	0.93085015
	fucose degradation	0.8578455	33.24	32.2	0.98	1	Control <= OVX	0.95429879
	methanogenesis from carbon dioxide	0.8640013	33.23	32.23333333	0.98	1	Control <= OVX	0.95429879
	sucrose degradation II	0.870096	32.78	33.73333333	1	1	OVX <= Control	0.95429879
	4-aminobutyrate degradation	0.8823449	32.8	33.66666667	1	1	OVX <= Control	0.95743809
	homoacetogenesis	0.9070041	32.84	33.53333333	1	1	OVX <= Control	0.97383596
	propionate production II	0.9317485	32.88	33.4	1	1	OVX <= Control	0.98831816
	glutamine degradation II	0.9503291	32.91	33.3	1	1	OVX <= Control	0.98831816
	methionine degradation I	0.9565409	32.92	33.26666667	1	1	OVX <= Control	0.98831816
	galacturonate degradation I	0.9627281	33.07	32.76666667	1	1	Control <= OVX	0.98831816
	rhamnose degradation	0.9689394	33.06	32.8	0.98	1	Control <= OVX	0.98831816
	alanine degradation II	0.9813659	32.96	33.13333333	1	1	OVX <= Control	0.9910824
	glutamine degradation I	1	32.99	33.03333333	1	1	OVX <=> Control	1
	galacturonate degradation II	NaN	33	33	0	0	OVX <=> Control	NaN
	pectin degradation I	5.70E-05	38.18	15.73333333	1	1	Control < OVX	0.002907
	arginine degradation III	4.88E-05	27.79	50.36666667	0.78	0.9333333	OVX < Control	0.002907
	arabinoxylan degradation	0.0001025	38	16.33333333	1	1	Control < OVX	0.003369647
	fructan degradation	0.0001321	37.92	16.6	1	1	Control < OVX	0.003369647
	mucin degradation	0.0002311	37.74	17.2	1	1	Control < OVX	0.004715236
	butyrate production I	0.0003387	28.39	48.36666667	1	1	OVX < Control	0.005758512
	pyruvate dehydrogenase complex	0.0005149	28.53	47.9	1	1	OVX < Control	0.007503353
	starch degradation	0.0009376	37.26	18.8	1	1	Control < OVX	0.010625771
	sulfate reduction (dissimilatory)	0.0008613	37.29	18.7	1	1	Control < OVX	0.010625771
	galactose degradation	0.0012995	28.86	46.8	1	1	OVX < Control	0.0132554
	glutamine degradation II	0.0016132	37.06	19.46666667	1	1	Control < OVX	0.014958912
	Bifidobacterium shunt	0.0018837	29	46.33333333	1	1	OVX < Control	0.016011671
	triacylglycerol degradation	0.0021512	29.05	46.16666667	1	1	OVX < Control	0.016878419
	allose degradation	0.0030127	29.18	45.73333333	1	1	OVX < Control	0.021949781
	glycerol degradation I	0.0038617	29.28	45.4	1	1	OVX < Control	0.02461849
	4-aminobutyrate degradation	0.0036777	36.74	20.53333333	1	1	Control < OVX	0.02461849
	acetate to acetyl-CoA	0.0048183	29.37	45.1	1	1	OVX < Control	0.028909848
	lactose degradation	0.0054486	36.58	21.06666667	1	1	Control < OVX	0.030875428
	glycine degradation	0.0068781	29.52	44.6	1	1	OVX < Control	0.036924349
	ethanol production I	0.0074016	36.45	21.5	1	1	Control < OVX	0.037748344
	maltose degradation	0.0079254	29.58	44.4	1	1	OVX < Control	0.038494664
	aspartate degradation II	0.0084815	36.39	21.7	1	1	Control < OVX	0.039323318
	pectine degradation II	0.0113691	36.26	22.13333333	1	1	Control < OVX	0.050419403
	glycerol degradation II	0.0129873	29.8	43.66666667	1	1	OVX < Control	0.055196012
	serine degradation	0.0168404	29.92	43.26666667	1	1	OVX < Control	0.068708693
	methanogenesis - methyl-coM	0.0179468	29.95	43.16666667	0.98	1	OVX < Control	0.070406587
	butyrate production II	0.0186641	29.97	43.1	1	1	OVX < Control	0.070508939
	tyrosine degradation II	0.0211941	30.03	42.9	1	1	OVX < Control	0.077207246
	alanine degradation II	0.0329004	35.75	23.83333333	1	1	Control < OVX	0.115718571
	Entner-Doudoroff pathway	0.0348735	30.28	42.06666667	1	1	OVX < Control	0.118569859
	alanine degradation I	0.0413391	35.63	24.23333333	1	1	Control < OVX	0.13028133
	lysine degradation I	0.0421498	30.38	41.73333333	0.98	1	OVX < Control	0.13028133
	glycolysis (pay-off phase)	0.0397347	30.35	41.83333333	1	1	OVX < Control	0.13028133
	pentose phosphate pathway (non-oxidativ	0.0514974	30.49	41.36666667	1	1	OVX <= Control	0.154492194
	methionine degradation I	0.0721243	35.32	25.26666667	1	1	Control <= OVX	0.193475551
	cysteine biosynthesis/homocysteine degrac	0.0732402	30.69	40.7	1	1	OVX <= Control	0.193475551
	cysteine degradation II	0.0757543	30.71	40.63333333	1	1	OVX <= Control	0.193475551
	lysine degradation II	0.0745615	30.7	40.66666667	1	1	OVX <= Control	0.193475551
	pyruvate:ferredoxin oxidoreductase	0.0758728	35.29	25.36666667	1	1	Control <= OVX	0.193475551
	propionate production II	0.0746059	35.3	25.33333333	1	1	Control <= OVX	0.193475551
	threonine degradation II	0.0811209	35.25	25.5	1	1	Control <= OVX	0.201813063
	threonine degradation I	0.085273	35.22	25.6	1	1	Control <= OVX	0.207091671
	leucine degradation	0.099578	30.88	40.06666667	1	1	OVX <= Control	0.236208182
	rhamnose degradation	0.1068154	30.92	39.93333333	1	1	OVX <= Control	0.247617481
	ethanol production II	0.1308647	34.95	26.5	1	1	Control <= OVX	0.296626721
	glutamate degradation II	0.154226	31.16	39.13333333	1	1	OVX <= Control	0.33259857
	glutamine degradation I	0.156517	31.17	39.1	1	1	OVX <= Control	0.33259857
	pyruvate:formate lyase	0.1564989	34.83	26.9	1	1	Control <= OVX	0.33259857
	lactose and galactose degradation	0.1634371	34.8	27	1	1	Control <= OVX	0.340215954
	asparagine degradation	0.1730708	31.24	38.86666667	1	1	OVX <= Control	0.346141522
	acetyl-CoA to acetate	0.1729487	31.24	38.86666667	1	1	OVX <= Control	0.346141522
T3	arginine degradation II	0.1830788	34.72	27.26666667	1	1	Control <= OVX	0.359116104
	glycolysis (preparatory phase)	0.2070546	31.37	38.43333333	1	1	OVX <= Control	0.391103084
	lactaldehyde degradation	0.2042994	34.64	27.53333333	1	1	Control <= OVX	0.391103084
	melibiose degradation	0.2216106	34.58	27.73333333	1	1	Control <= OVX	0.410986873
	lactate production	0.2621515	31.55	37.83333333	1	1	OVX <= Control	0.477490307
	urea degradation	0.3114038	31.69	37.36666667	1	1	OVX <= Control	0.557248816
	methanogenesis from carbon dioxide	0.3265743	31.73	37.23333333	0.94	1	OVX <= Control	0.574320396
	arginine degradation I	0.3620733	31.82	36.93333333	1	1	OVX <= Control	0.625957234
	arabinose degradation	0.3831227	34.13	29.23333333	1	1	Control <= OVX	0.651308563
	histidine degradation	0.4002658	34.09	29.36666667	1	1	Control <= OVX	0.669296955
	methanol conversion	0.4453715	32.01	36.3	1	1	OVX <= Control	0.721230638
	succinate production	0.445466	33.99	29.7	1	1	Control <= OVX	0.721230638
	fucose degradation	0.468684	32.06	36.13333333	1	1	OVX <= Control	0.735740608
	homoacetogenesis	0.4688543	32.06	36.13333333	1	1	OVX <= Control	0.735740608
	glutamate degradation III	0.5592706	32.24	35.53333333	1	1	OVX <= Control	0.837416682
	anaerobic fatty acid beta-oxidation	0.5911177	32.3	35.33333333	1	1	OVX <= Control	0.837416682
	glycerol degradation III	0.5856942	33.71	30.63333333	1	1	Control <= OVX	0.837416682
	glyoxylate bypass	0.5800726	33.72	30.6	1	1	Control <= OVX	0.837416682
	pentose phosphate pathway (oxidative pha	0.5486407	32.22	35.6	1	1	OVX <= Control	0.837416682
	lactate consumption I	0.5804371	33.72	30.6	1	1	Control <= OVX	0.837416682
	succinate conversion to propionate	0.5591761	33.76	30.46666667	0.92	0.9333333	Control <= OVX	0.837416682
	xylose degradation	0.6018496	32.32	35.26666667	1	1	OVX <= Control	0.840940543
	putrescine degradation	0.6292594	33.63	30.9	1	1	Control <= OVX	0.867357485
	fructose degradation	0.6515298	32.41	34.96666667	1	1	OVX <= Control	0.886080546
	valine degradation I	0.6682138	33.56	31.13333333	0.98	1	Control <= OVX	0.896813211
	ribose degradation	0.684663	33.53	31.23333333	0.76	1	Control <= OVX	0.906956184
	sucrose degradation I	0.7377274	33.44	31.53333333	1	1	Control <= OVX	0.913893553
	trehalose degradation	0.7436585	33.43	31.56666667	1	1	Control <= OVX	0.913893553
	phenylalanine degradation	0.7084055	33.49	31.36666667	1	1	Control <= OVX	0.913893553
	aspartate degradation I	0.7085516	32.51	34.63333333	1	1	OVX <= Control	0.913893553
	proline degradation	0.7260837	32.54	34.53333333	1	1	OVX <= Control	0.913893553
	succinate consumption	0.7260434	33.46	31.46666667	1	1	Control <= OVX	0.913893553
	mannose degradation	0.7792478	32.63	34.23333333	1	1	OVX <= Control	0.924224114
	tryptophan degradation	0.7733005	33.38	31.73333333	1	1	Control <= OVX	0.924224114
	acetyl-CoA to crotonyl-CoA	0.761371	33.4	31.66666667	1	1	Control <= OVX	0.924224114
	methionine degradation II	0.7911616	32.65	34.16666667	1	1	OVX <= Control	0.927568778
	galacturonate degradation I	0.8394978	32.73	33.9	1	1	OVX <= Control	0.934246056
	isoleucine degradation	0.8639631	32.77	33.76666667	1	1	OVX <= Control	0.934246056
	cysteine degradation I	0.8517639	32.75	33.83333333	1	1	OVX <= Control	0.934246056
	arginine degradation IV	0.8701311	32.78	33.73333333	1	1	OVX <= Control	0.934246056
	NADH:ferredoxin oxidoreductase	0.8274282	32.71	33.96666667	1	1	OVX <= Control	0.934246056
	propionate production I	0.8274375	32.71	33.96666667	0.96	1	OVX <= Control	0.934246056
	propionate production III	0.8640013	32.77	33.76666667	1	1	OVX <= Control	0.934246056
	hydrogen metabolism	0.8517751	33.25	32.16666667	1	1	Control <= OVX	0.934246056
	formate conversion	0.8885187	32.81	33.63333333	1	1	OVX <= Control	0.944051115
	tyrosine degradation I	0.9441072	32.9	33.33333333	1	1	OVX <= Control	0.96298938
	glutamate degradation I	0.9317232	32.88	33.4	0.9	0.9333333	OVX <= Control	0.96298938
	arginine degradation V	0.9255367	32.87	33.43333333	1	1	OVX <= Control	0.96298938
	lactate consumption II	0.9378933	32.89	33.36666667	1	1	OVX <= Control	0.96298938
	sucrose degradation II	0.9813508	33.04	32.86666667	1	1	Control <= OVX	0.981365089
	nitrate reduction (dissimilatory)	0.9813651	32.96	33.13333333	1	1	OVX <= Control	0.981365089
	galacturonate degradation II	NaN	33	33	0	0	OVX <=> Control	NaN
	glycolysis (pay-off phase)	0.0023217	29.08	46.06666667	1	1	OVX < Control	0.09431546
	glutamate degradation III	0.0035034	36.76	20.46666667	1	1	Control < OVX	0.09431546
	butyrate production I	0.0043588	29.33	45.23333333	1	1	OVX < Control	0.09431546
	aspartate degradation II	0.0043776	36.67	20.76666667	1	1	Control < OVX	0.09431546
	lactate consumption I	0.0048262	29.37	45.1	1	1	OVX < Control	0.09431546
	glycine degradation	0.005548	29.43	44.9	1	1	OVX < Control	0.09431546
	Entner-Doudoroff pathway	0.0072307	29.54	44.53333333	1	1	OVX < Control	0.10536205
	acetyl-CoA to acetate	0.0097368	36.33	21.9	1	1	Control < OVX	0.12102816
	hydrogen metabolism	0.0116505	36.25	22.16666667	1	1	Control < OVX	0.12102816
	arabinose degradation	0.0118655	36.24	22.2	1	1	Control < OVX	0.12102816
	sulfate reduction (dissimilatory)	0.0292654	35.81	23.63333333	1	1	Control < OVX	0.27137048
	allose degradation	0.0525436	35.5	24.66666667	1	1	Control <= OVX	0.42644692
	rhamnose degradation	0.0543511	35.48	24.73333333	0.98	1	Control <= OVX	0.42644692
	leucine degradation	0.0604248	35.42	24.93333333	1	1	Control <= OVX	0.44023819
	arginine degradation V	0.0820749	35.24	25.53333333	1	1	Control <= OVX	0.49736493
	formate conversion	0.0852218	35.22	25.6	1	1	Control <= OVX	0.49736493
	sucrose degradation II	0.0910477	35.18	25.73333333	1	1	Control <= OVX	0.49736493
	valine degradation I	0.0954548	35.15	25.83333333	1	1	Control <= OVX	0.49736493
	asparagine degradation	0.1004404	30.88	40.06666667	1	1	OVX <= Control	0.49736493
	pentose phosphate pathway (oxidative pha	0.1086046	30.93	39.9	1	1	OVX <= Control	0.49736493
	acetate to acetyl-CoA	0.1193013	35.01	26.3	1	1	Control <= OVX	0.49736493
	acetyl-CoA to crotonyl-CoA	0.1193942	35.01	26.3	1	1	Control <= OVX	0.49736493
	succinate consumption	0.1231348	34.99	26.36666667	1	1	Control <= OVX	0.49736493
	serine degradation	0.1309195	31.05	39.5	1	1	OVX <= Control	0.49736493
	mannose degradation	0.1348644	31.07	39.43333333	1	1	OVX <= Control	0.49736493
	fucose degradation	0.1348942	34.93	26.56666667	0.98	1	Control <= OVX	0.49736493
	ethanol production I	0.1349539	34.93	26.56666667	1	1	Control <= OVX	0.49736493
	fructose degradation	0.1411191	31.1	39.33333333	1	1	OVX <= Control	0.49736493
	tryptophan degradation	0.1454322	31.12	39.26666667	1	1	OVX <= Control	0.49736493
	cysteine degradation I	0.1497509	31.14	39.2	1	1	OVX <= Control	0.49736493
	triacylglycerol degradation	0.1540909	34.84	26.86666667	1	1	Control <= OVX	0.49736493
	isoleucine degradation	0.1563766	34.83	26.9	1	1	Control <= OVX	0.49736493
	methanol conversion	0.1633911	34.8	27	1	1	Control <= OVX	0.49736493
	lysine degradation I	0.1657883	31.21	38.96666667	1	1	OVX <= Control	0.49736493
	propionate production I	0.1755252	34.75	27.16666667	1	0.9333333	Control <= OVX	0.51153063
	galacturonate degradation I	0.2042401	31.36	38.46666667	1	1	OVX <= Control	0.5637818
	glycerol degradation III	0.207144	31.37	38.43333333	1	1	OVX <= Control	0.5637818
	arginine degradation I	0.2100364	34.62	27.6	1	1	Control <= OVX	0.5637818
	arginine degradation III	0.2204573	31.42	38.26666667	0.84	0.8	OVX <= Control	0.57658052
	propionate production III	0.2365908	31.47	38.1	1	1	OVX <= Control	0.60330646
	pyruvate:formate lyase	0.2428774	34.51	27.96666667	1	1	Control <= OVX	0.6042315
	histidine degradation	0.2586964	31.54	37.86666667	1	1	OVX <= Control	0.61041604
	glycolysis (preparatory phase)	0.265516	34.44	28.2	1	1	Control <= OVX	0.61041604
	nitrate reduction (dissimilatory)	0.2689148	31.57	37.76666667	1	1	OVX <= Control	0.61041604
	mucin degradation	0.2792021	31.6	37.66666667	1	1	OVX <= Control	0.61041604
	butyrate production II	0.2858672	31.62	37.6	1	1	OVX <= Control	0.61041604
	4-aminobutyrate degradation	0.2859304	31.62	37.6	1	1	OVX <= Control	0.61041604
	alanine degradation I	0.2931283	31.64	37.53333333	1	1	OVX <= Control	0.61041604
	anaerobic fatty acid beta-oxidation	0.2932391	34.36	28.46666667	1	1	Control <= OVX	0.61041604
	galactose degradation	0.304013	31.67	37.43333333	1	1	OVX <= Control	0.62018651
	NADH:ferredoxin oxidoreductase	0.3114989	31.69	37.36666667	1	1	OVX <= Control	0.62299788
T4	threonine degradation II	0.3189277	34.29	28.7	1	1	Control <= OVX	0.625589
	xylose degradation	0.3265532	31.73	37.23333333	1	1	OVX <= Control	0.62846086
	glycerol degradation I	0.3541205	34.2	29	1	1	Control <= OVX	0.66889437
	ethanol production II	0.3621626	34.18	29.06666667	1	1	Control <= OVX	0.67164694
	methionine degradation II	0.3915823	31.89	36.7	1	1	OVX <= Control	0.69189273
	melibiose degradation	0.3961137	34.1	29.33333333	1	1	Control <= OVX	0.69189273
	proline degradation	0.4181274	34.05	29.5	1	1	Control <= OVX	0.69189273
	lactose degradation	0.4317012	34.02	29.6	1	1	Control <= OVX	0.69189273
	lysine degradation II	0.4361245	34.01	29.63333333	1	1	Control <= OVX	0.69189273
	succinate conversion to propionate	0.4362248	31.99	36.36666667	0.94	1	OVX <= Control	0.69189273
	arabinoxylan degradation	0.4408765	34	29.66666667	1	1	Control <= OVX	0.69189273
	lactate production	0.4592443	32.04	36.2	1	1	OVX <= Control	0.69189273
	pyruvate:ferredoxin oxidoreductase	0.4595389	33.96	29.8	1	1	Control <= OVX	0.69189273
	propionate production II	0.4595389	32.04	36.2	1	1	OVX <= Control	0.69189273
	maltose degradation	0.4641167	32.05	36.16666667	1	1	OVX <= Control	0.69189273
	glyoxylate bypass	0.4641558	33.95	29.83333333	1	1	Control <= OVX	0.69189273
	methanogenesis - methyl-coM	0.4690487	32.06	36.13333333	1	1	OVX <= Control	0.69189273
	glutamine degradation II	0.4738175	32.07	36.1	1	1	OVX <= Control	0.69189273
	pectin degradation I	0.4786758	33.92	29.93333333	1	1	Control <= OVX	0.69189273
	arginine degradation II	0.4882855	33.9	30	1	1	Control <= OVX	0.69189273
	methionine degradation I	0.4883949	32.1	36	1	1	OVX <= Control	0.69189273
	fructan degradation	0.4982194	32.12	35.93333333	1	1	OVX <= Control	0.69614215
	tyrosine degradation II	0.5232062	32.17	35.76666667	1	1	OVX <= Control	0.72117608
	starch degradation	0.5593049	32.24	35.53333333	1	1	OVX <= Control	0.73567882
	cysteine degradation II	0.5643803	32.25	35.5	1	1	OVX <= Control	0.73567882
	lactaldehyde degradation	0.5644059	32.25	35.5	1	1	OVX <= Control	0.73567882
	pectine degradation II	0.5696344	32.26	35.46666667	1	1	OVX <= Control	0.73567882
	glutamate degradation II	0.5697905	32.26	35.46666667	1	1	OVX <= Control	0.73567882
	glutamine degradation I	0.5911501	32.3	35.33333333	1	1	OVX <= Control	0.75371637
	sucrose degradation I	0.612692	33.66	30.8	1	1	Control <= OVX	0.77153808
	homoacetogenesis	0.6291581	32.37	35.1	1	1	OVX <= Control	0.78261133
	glutamate degradation I	0.6456945	32.4	35	0.9	0.8	OVX <= Control	0.79350409
	pentose phosphate pathway (non-oxidativ	0.685454	32.47	34.76666667	1	1	OVX <= Control	0.82673456
	pyruvate dehydrogenase complex	0.6912492	32.48	34.73333333	1	1	OVX <= Control	0.82673456
	threonine degradation I	0.6970507	33.51	31.3	1	1	Control <= OVX	0.82673456
	glycerol degradation II	0.7201735	33.47	31.43333333	1	1	Control <= OVX	0.83480087
	lactose and galactose degradation	0.7202204	32.53	34.56666667	1	1	OVX <= Control	0.83480087
	ribose degradation	0.7314507	33.45	31.5	0.86	0.8	Control <= OVX	0.83829182
	trehalose degradation	0.7436639	32.57	34.43333333	1	1	OVX <= Control	0.83997929
	Bifidobacterium shunt	0.7493933	32.58	34.4	1	1	OVX <= Control	0.83997929
	phenylalanine degradation	0.7732113	33.38	31.73333333	1	1	Control <= OVX	0.85725605
	arginine degradation IV	0.8032598	32.67	34.1	1	1	OVX <= Control	0.88099462
	urea degradation	0.8885393	33.19	32.36666667	1	1	Control <= OVX	0.96415964
	aspartate degradation I	0.9131945	32.85	33.5	1	1	OVX <= Control	0.97325932
	succinate production	0.9193843	32.86	33.46666667	1	1	OVX <= Control	0.97325932
	cysteine biosynthesis/homocysteine degrac	0.9255505	33.13	32.56666667	1	1	Control <= OVX	0.97325932
	methanogenesis from carbon dioxide	0.9565371	33.08	32.73333333	0.98	1	Control <= OVX	0.97514815
	alanine degradation II	0.962742	32.93	33.23333333	1	1	OVX <= Control	0.97514815
	lactate consumption II	0.9689316	33.06	32.8	1	1	Control <= OVX	0.97514815
	putrescine degradation	0.9689451	32.94	33.2	1	1	OVX <= Control	0.97514815
	tyrosine degradation I	0.9751481	32.95	33.16666667	1	1	OVX <= Control	0.97514815
	galacturonate degradation II	NaN	33	33	0	0	OVX <=> Control	NaN
